# Hemilability Modulation via Phosphane-Triazole Ligand Design: Impact on Catalytic Formic Acid Dehydrogenation

**DOI:** 10.1021/acs.inorgchem.5c03962

**Published:** 2025-12-11

**Authors:** Susana García-Abellán, Andrea Pérez-García, Daniel Barrena-Espés, Miguel A. Casado, Julen Munarriz, Vincenzo Passarelli, Manuel Iglesias

**Affiliations:** † Instituto de Síntesis Química y Catálisis Homogénea (ISQCH), CSIC-Universidad de Zaragoza, C/Pedro Cerbuna 12, Zaragoza 50009, Spain; ‡ Departamento de Química Física and Instituto de Biocomputación y Física de Sistemas Complejos (BIFI), 16765 Universidad de Zaragoza, Zaragoza 50009, Spain; § Departamento de Química Física y Analítica, 16763 Universidad de Oviedo, Oviedo 33006, Spain

## Abstract

Two novel P–N ligands, 1-[2-(diphenylphosphanyl)­ethyl]-1*H*-benzo-1,2,3-triazole (**1**) and its N2-isomer (**2**), were synthesized. Reaction of **1** and **2** with [Ir­(μ-Cl)­(cod)]_2_ and [Rh­(μ-Cl)­(cod)]_2_ in a 2:1 molar ratio followed by AgBF_4_ led to the formation of square-planar κ^2^-*P*,*N* complexes, **Ir-1**, **Ir-2**, **Rh-1** and **Rh-2**. Density functional theory studies provided insights into the electronic structure and bonding of the complexes. Complex **Ir-3** was also prepared for comparison, employing ligand **3**, 1-[2-(diphenylphosphanyl)­methyl]-1*H*-benzo-1,2,3-triazole. Variable-temperature NMR studies on [IrCl­(cod)­(P–N)] complexes revealed fluxional behavior attributed to ligand hemilability. Activation Gibbs free energies (Δ*G* ^‡^) for the isomerization equilibrium of [IrCl­(cod)­(PN)] complexes featuring ligands **1**, **2** and **3** are 10.24, 10.60, and 8.87 kcal·mol^–1^, respectively. This enabled us to propose a coordination-ability scale that follows the trend **3** > **1** > **2**. The relative activities of the iridium complexes were evaluated in the dehydrogenation of formic acid. Under optimized conditions, in an HCOOH/Et_3_N mixture, the initial TOFs are 186, 828, and 948 h^–1^ for **Ir-1**, **Ir-2**, and **Ir-3**, respectively. This indicates that **Ir-3**, bearing the most strongly coordinating ligand, exhibits the highest catalytic activity, reaching a TON value of 444 after 7 h. This study demonstrates the tunability of the hemilability of benzo-1,2,3-triazole-based P–N ligands and their potential for modulating catalytic activity.

## Graphical abstract

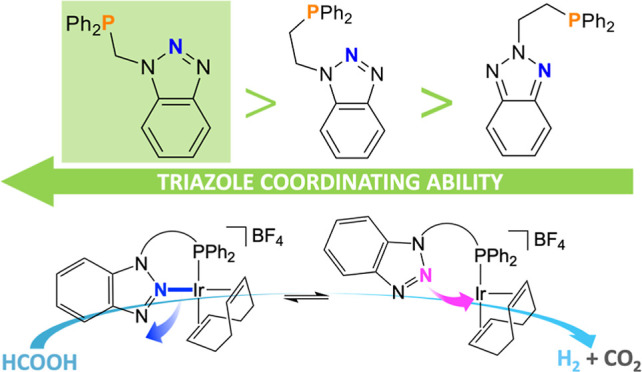

## Introduction

In recent decades, the search for carbon-neutral energy systems has intensified in response to growing environmental concerns and the finite nature of fossil resources.^1^ Among the various alternatives, hydrogen has emerged as a key candidate for a sustainable energy transition, providing clean energy through electrochemical reactions in fuel cells where water is the only byproduct. However, challenges related to the storage and transport of hydrogen gas have led to interest in liquid organic hydrogen carriers (LOHCs).^1–7^ Among these, formic acid (FA) warrants particular attention due to its multiple advantages: it can be produced via CO_2_ hydrogenation, contributing to a carbon-neutral hydrogen storage cycle, and its dehydrogenation provides high-purity hydrogen suitable for fuel cell applications.^1^ In addition, FA offers higher energy density and lower toxicity compared to other LOHCs such as ammonia or methanol.

The minimization of the solvent volume in the FA reaction mixture is advantageous, as it enhances the hydrogen content of the system.^8^ In this context, several literature examples of catalysts that operate under solventless conditions have been reported.^9–13^ HCOOH/Et_3_N mixtures have attracted substantial research interest, with many efficient catalysts reported.^6,9,14–18^ However, examples of systems operating in the neat mixture are still scarce,^19–23^ with the most active catalyst to date being [RuCl_2_(dmso)_4_].^23^

Since the proposal of FA as a LOHC by Beller^6^ and Laurenczy^24^ in 2008, the catalytic dehydrogenation of FA has attracted considerable interest. This reaction has seen notable enhancements triggered by ligand design. In particular, the development of specific ligands, such as *N*-heterocyclic carbenes, phosphane-based systems, and nitrogen donors, has been essential to the design of catalysts with exceptional stability and activity under mild conditions.^9–11,13,14,22,25–27^ Ligand properties—such as donor strength, steric bulk, geometry, and denticity—along with metal–ligand bond strength, govern metal complex reactivity.^28^ Thus, the detailed understanding of these properties is key to improve catalytic performance.^29^

Triazoles represent an intriguing class of ligands due to their highly versatile and modifiable architecture. The azide–alkyne Huisgen cycloaddition, a prominent example of “click chemistry,” is a prolific method for the synthesis of 1,2,3-triazoles.^30^ This synthetic methodology has significantly enhanced the availability of this class of compounds, allowing them to become ubiquitous motifs in chemical and pharmaceutical research.^31,32^ The use of 1,2,3-triazoles as coordinating moieties in polydentate ligands has experienced a great growth in recent years, which can be attributed to the success of “click” reactions, but also to the unique electronic properties of these molecules.^33–35^

While numerous examples of triazole-based ligands already exist, their inherent structural flexibility allows for a wide range of modifications, making them ideal candidates for the design of tailored catalysts. Different types of bidentate triazole ligands are depicted in Figure 1. C4-functionalized 1,2,3-triazoles that coordinate by the N3 and the donor group at the C4 moiety in a chelating fashion are the most widespread structures for this type of ligands (**A**).^33–46^ On the other hand, N1-functionalized 1,2,3-triazole ligands are less frequent. Coordination by the functional group at N1 in this type of ligands usually imposes chelation by the N2 (**B**),^36,42,43,47–52^ although the more stable coordination by the N3 is sometimes able to overcome the chelate effect.^42,49^ Finally, examples of N2-substituted 1,2,3-triazole polydentate ligands are hitherto unknown (**C**).

**Figure 1:**
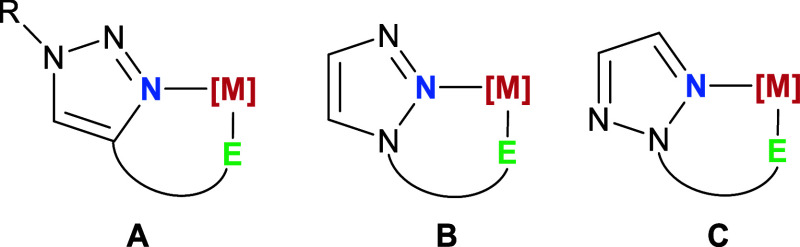
Schematic representation of the three different types of bidentate 1,2,3-triazole ligands coordinated to a metal center ([M]) by a generic donor group (E): (A) C4-functionalized 1,2,3-triazole; (B) N1-functionalized 1,2,3-triazole; and (C) N2-substituted 1,2,3-triazole.

**Figure 2:**
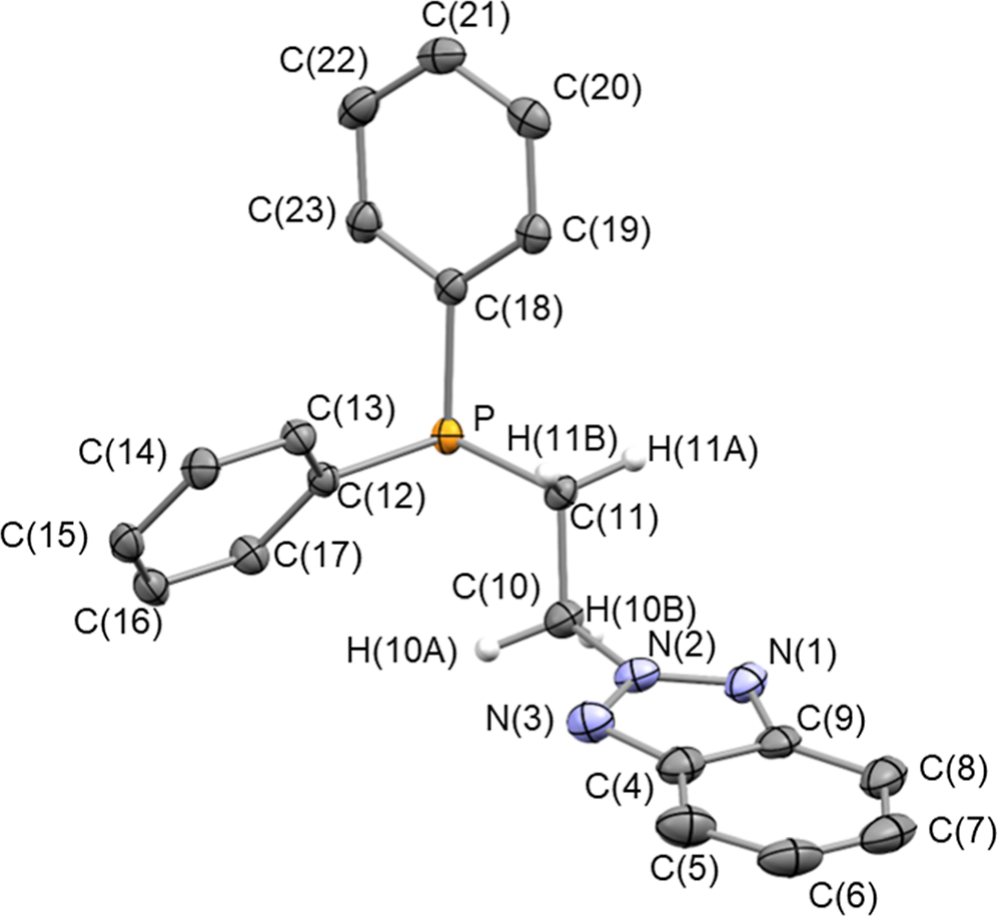
ORTEP view of **2** with 50% probability ellipsoids. For clarity, most hydrogen atoms have been omitted. Selected bond lengths (Å) and angles (deg): P–C(11) 1.8516(15), N(1)–N(2) 1.320(2), N(2)–N(3) 1.335(2), N(2)–C(10) 1.459(2), C(10)–C(11) 1.526(2), N(2)–N(1)–C(9) 101.72(14), N(1)–N(2)–N(3) 119.03(14), N(1)–N(2)–C(10) 122.47(14), N(3)–N(2)–C(10) 118.48(14), N(1)–C(9)–C(8) 130.33(18), N(1)–C(9)–C(4) 107.89(15), N(2)–N(3)–C(4) 101.76(15), N(3)–C(4)–C(9) 109.59(16), N(3)–C(4)–C(5) 128.82(19).

**Figure 3:**
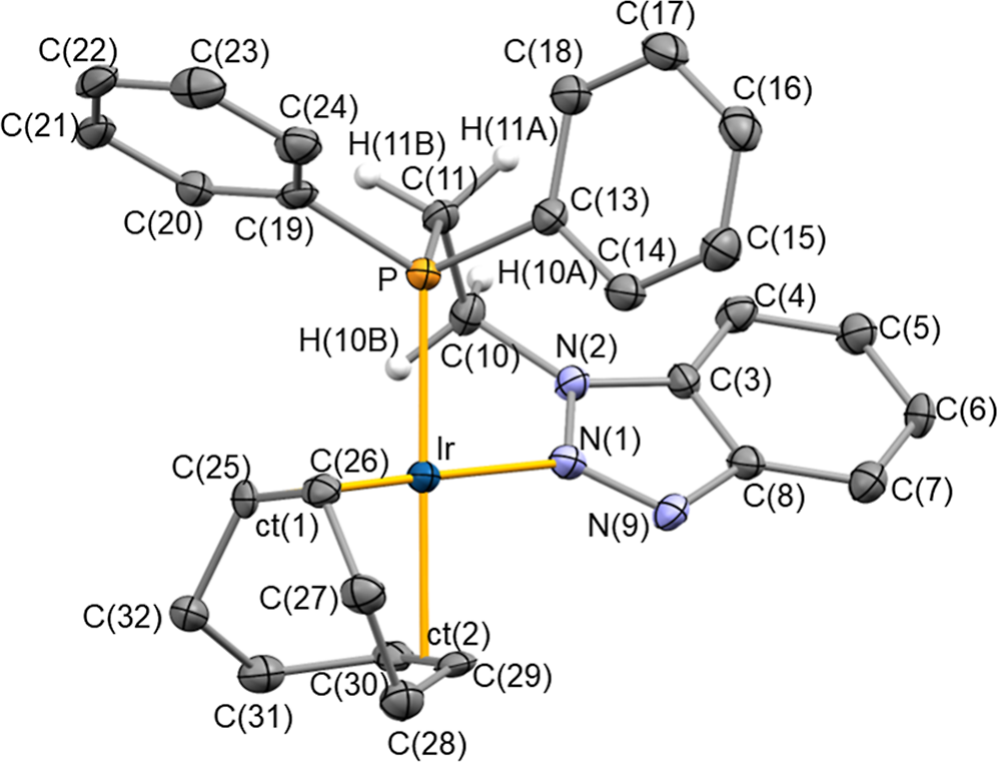
ORTEP view of **Ir-1** with 50% probability ellipsoids. For clarity, the BF_4_^–^ counterion and most hydrogen atoms have been omitted. Selected bond lengths (Å) and angles (deg): Ir–P 2.3011(11), Ir–N(1) 2.073(3), Ir-ct(1) 2.0177(2), Ir-ct(2) 2.0858(3), C(25)–C(26) 1.409(6), C(29)–C(30) 1.381(6), N(1)–N(9) 1.316(5), N(1)–N(2) 1.358(5); N(2)–C(10)–C(11) 112.5(3), N(9)–N(1)–N(2) 110.8(3), N(9)–N(1)–Ir 125.6(3), N(2)–N(1)–Ir 122.8(3), N(1)–N(2)–C(10) 120.7(3), C(11)–P–Ir 109.09(14), N(1)–Ir–P 86.25(10), C(10)–C(11)–P 112.1(3), ct(1)-Ir-ct(2) 86.818(8), ct(1)-Ir–N(1) 178.83(10). ct(1) and ct(2) are the centroids of C(25) and C(26), and C(29) and C­(30), respectively.

**A**- and **B**-type structures—usually referred to as “regular” and “inverse” coordination,^35^ respectively—have been studied in detail by several groups, concluding that **A**-type structures lead to a more stable coordination due to the higher electron density at the N3 and the stronger π back-donation.^40–43^ Based on this rationale, **C**-type complexes are anticipated to exhibit stronger M–N bonds in comparison to **B**-type complexes.

**B**-type complexes have shown interesting catalytic activities in several processes, although examples of this class of catalysts are still scarce. Shi and co-workers have reported the dehydration of propargyl alcohols catalyzed by an Fe­(III) complex that features an inverse benzo-1,2,3-triazole ligand. Notably, the use of related ligands based on pyridine, 2-imidazole, 1,2,4-triazole or tetrazole scaffolds resulted in inactive catalysts.^47^ Shi and Wang described a remarkably active and selective Au­(III) catalyst for the sequential Meyer-Schuster rearrangement of propargyl alcohols followed by allene halogenation. This catalyst features a 1,2,3-triazole ligand functionalized with a pyridine group at the N1 position, which effectively stabilizes the Au­(III) cation and enhances its activity due to the electron-deficient nature of the ligand.^51^ Another interesting example of triazole-based ancillary ligands in catalysis is found in the amino-triazoles reported by Leitner and co-workers. These ligands allow for the preparation of Mn­(I) complexes that efficiently catalyze the transfer hydrogenation of ketones.^48^

P–N ligands are versatile frameworks in Ir and Rh catalysis, offering a balance between electronic tunability (via the P donor) and geometric control (via the N donor). Prominent examples include the asymmetric hydrogenation catalysts reported by Pfaltz et al.^53,54^ as well as axially chiral P–N ligands.^55^

In the present study, we report on the synthesis of two novel hemilabile P–N bidentate ligands based on a benzo-1,2,3-triazole-derived structure. The coordination chemistry of these ligands was explored with Ir­(I) and Rh­(I) complexes, which has enabled the synthesis of a battery of **B**- and **C**-type complexes—the latter being the first examples described so far in the literature. The structure and bonding of the resulting complexes were evaluated using both computational and experimental studies. Finally, the catalytic activity of the Ir complexes in FAD was investigated under various reaction conditions, revealing strong structure–activity relationships.

## Results and Discussion

### Synthesis of P–N Ligands

The new P–N ligands 1-[2-(diphenylphosphanyl)­ethyl]-1*H*-benzo-1,2,3-triazole (**1**) and 2-[2-(diphenylphosphanyl)­ethyl]-1*H*-benzo-1,2,3-triazole (**2**) (Scheme 1) were synthesized from benzo-1,2,3-triazole. In a first step, alkylation of the starting material was carried out using 1,2-dibromoethane as the solvent in the presence of potassium carbonate and tetrabutylammonium iodide (TBAI), giving a mixture of two benzo-1,2,3-triazoles substituted at N1 and N2, in a ratio of 8:2, respectively. The two isomers were separated on the basis of their different solubilities in pentane and purified by crystallization. Finally, each isomer, 1-(2-bromoethyl)-1*H*-benzo-1,2,3-triazole and 2-(2-bromoethyl)-1*H*-benzo-1,2,3-triazole, was separately reacted with KPPh_2_ in tetrahydrofuran (THF), yielding the new P–N ligands **1** and **2**, respectively (Scheme 1).

**Scheme 1:**
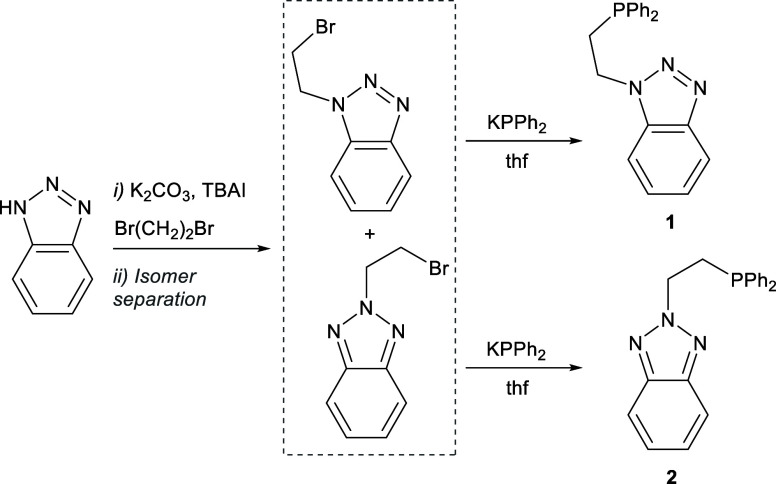
Synthetic Route for the Preparation of **1** and **2**

The bidentate coordination of ligand **1** leads to the formation of a **B**-type complex (Figure 1). An examination of the resonance structures of ligand **1** (Scheme 2) indicates that structure **I**, which lacks formal charge separation, most accurately represents the actual electronic structure. Note that the presence of a negative charge on N2 implies the partial dearomatization of the benzene ring (**III**; Scheme 2).

**Scheme 2:**
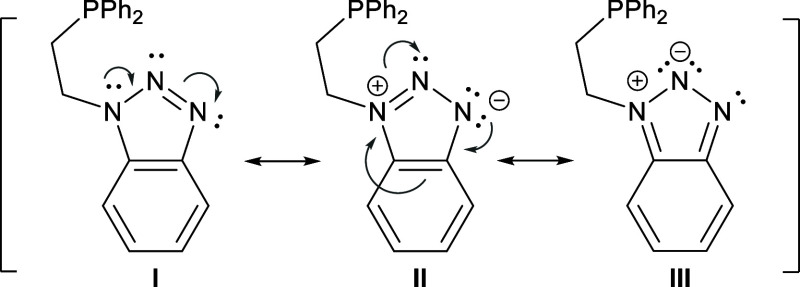
Resonance Structures of **1**

In the case of **2**, a **C**-type ligand is formed upon coordination (Figure 1). Its electronic structure can be represented by resonance forms **IV**, **V**, and **VI**, with **V** and **VI** being equivalent (Scheme 3). Note that a resonance structure without formal charge separation implies the partial dearomatization of the ring, whereas maintaining ring aromatization requires charge separation, placing a negative charge on N1.

**Scheme 3:**
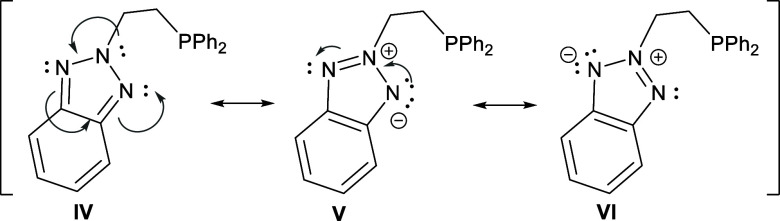
Resonance Structures of **2**

Since ligand **2** represents the first example of a **C**-type ligand to our knowledge, attempts were made to determine its crystal structure using single-crystal X-ray diffraction. Suitable crystals were obtained by slow diffusion of hexane into a solution of **2** in dichloromethane (Figure 2). In view of the observed bond lengths of N(1)–N(2) [1.320(2) Å], N(2)–N(3) [1.335(2) Å], and N(2)–C(10) [1.459(2) Å], and the planarity of the nitrogen atom N2 [Σ°_*N*(2)_ = 360.0(2)°], resonance structures **V** and **VI** should be the most relevant. Indeed, charge separation in **V** and **VI** preserves the aromaticity of the fused C_6_-ring and results in an sp^2^-hybridized N2 atom.

### Synthesis and Characterization of **Ir­(I)** Complexes

**1** was reacted with 0.5 equiv of [Ir­(μ-Cl)­(cod)]_2_ (cod = 1,5-cyclooctadiene) in dichloromethane at room temperature, resulting in the coordination of the phosphane group to the metal center. Subsequently, one equivalent of AgBF_4_ was added, yielding **Ir-1** (Scheme 4).

**Scheme 4:**
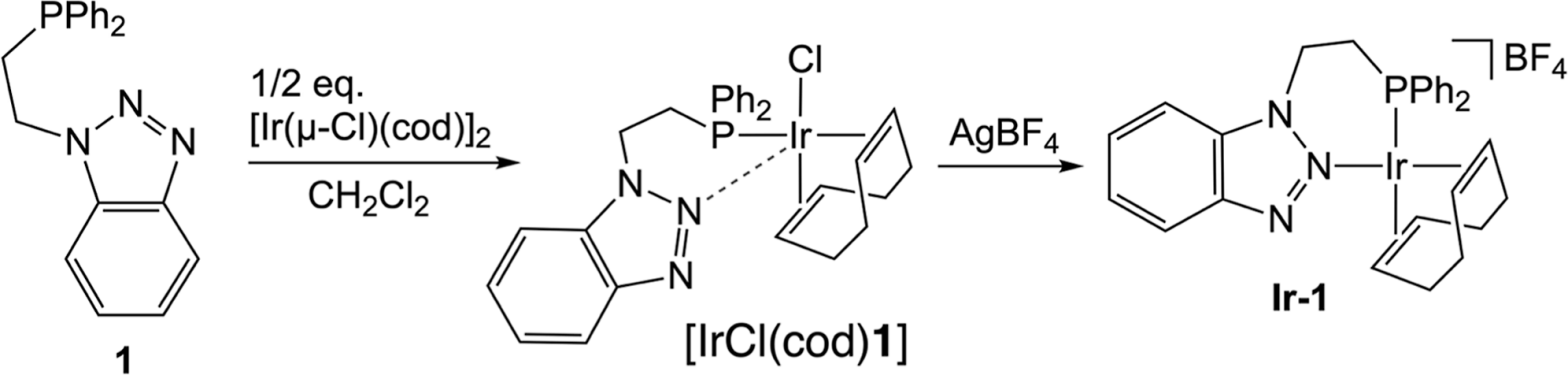
Synthesis of Complex **Ir-1**

In the ^31^P­{^1^H} NMR spectrum, after the addition of the Ir complex to **1**, a significant change is observed in the signal corresponding to the phosphorus nucleus, shifting from δ −21.4 in **1** to 11.6 ppm, indicating the coordination of the phosphane to yield the intermediate [IrCl­(cod)**1**] (Figure S50). The ^1^H NMR spectrum shows a clear shift for the two multiplets of the methylene protons from δ 4.79–4.67 and 2.83–2.72 ppm for the NCH_2_ and CH_2_P protons of **1**, respectively, to δ 5.46–5.33 and 3.36–3.18 ppm for the respective protons of [IrCl­(cod)**1**]. It is worth noting that, in this spectrum, signals corresponding to the aliphatic protons of cod are observed, while those of the olefinic protons are absent, likely due to a fluxional process that will be discussed later.

Regarding **Ir-1**, the most representative peaks in the ^1^H NMR spectrum are those corresponding to the methylene protons, which indicate the bidentate coordination of **1**. The signals of the CH_2_P protons shift to δ 3.02–2.92 ppm, while the NCH_2_ protons appear at similar chemical shifts but are resolved into two multiplets, each integrating to 1H, at δ 5.45–5.39 and 5.38–5.34 ppm, reflecting their diastereotopic nature. This arises from the conformation of the six-membered chelate ring formed upon κ^2^-*P*,*N* coordination, which positions the methylene groups outside the coordination plane, placing the geminal NCH_2_ protons in distinct chemical environments. Furthermore, triazole coordination restricts the free rotation of the *P*-substituents, rendering the aromatic protons of the phosphane phenyl groups nonequivalent. The ^31^P­{^1^H} NMR spectrum of **Ir-1** shows a singlet at δ 7.8 ppm, slightly downfield shifted compared to [IrCl­(cod)**1**].

Slow diffusion of hexane into a dichloromethane solution of complex **Ir-1** afforded crystals suitable for single-crystal X-ray diffraction analysis (Figure 3). **Ir-1** features a distorted square planar geometry, and contains **1** κ^2^ *P*,*N*-coordinated to the iridium center [Ir–P, 2.3011(11) Å; Ir–N(1), 2.073(3) Å], with a bite angle N(1)–Ir–P of 86.25(10)°. The remaining two coordination sites are occupied by the cod ligand [Ir-ct(1) 2.0177(2), Ir-ct(2) 2.0858(3) Å]. As a consequence of the trans influence of the phosphane group, higher than that of the triazole moiety, an elongated Ir-ct(2) distance [2.0858(3) Å] is observed when compared with the distance Ir-ct(1) [2.0177(2) Å]. Accordingly, the bond length C(29)–C(30) [1.381(6) Å] of the olefin group *trans* to the phosphane moiety is shorter than C(25)–C(26) [1.409(6) Å] trans to N(1), indicating a reduced π-back-donation to the olefin bond C(29)–C(30). The N(1)–N(2) bond length of 1.358(5) Å falls within the expected range for an aromatic N–N bond, while the N(1)–N(9) bond length of 1.316(5) Å is notably shorter, suggesting a double bond character, consistent with the neutral resonance structure **I**. The planarity of N(1) [Σ°_N(1)_ = 359.2(5)°] suggests an sp^2^ hybridization of N(1). Additionally, the metalacycle Ir–N(1)–N(2)–C(10)–C(11)–P adopts a boat conformation, with the Ir and C(10) atoms occupying the out-of-plane positions. The triazole ring is located out of the coordination plane [N(9)–N(1)–Ir–P 44.17(30)°], thus minimizing the ring strain of the metalacycle. Furthermore, the pitch and yaw angles of the triazole—these being the angles of inclination out-of-plane and in-plane, respectively, of the triazole ring with respect to the Ir–N bond—indicate a slightly deviated arrangement of the triazole ring with respect to the N(1)-Ir bond [yaw, ψ 1.5°; pitch, θ 9.0°].^56,57^

**Ir-2** was obtained via [IrCl­(cod)**2**] following a synthetic procedure analogous to that described above for **Ir-1** (see Scheme 5).

**Scheme 5:**
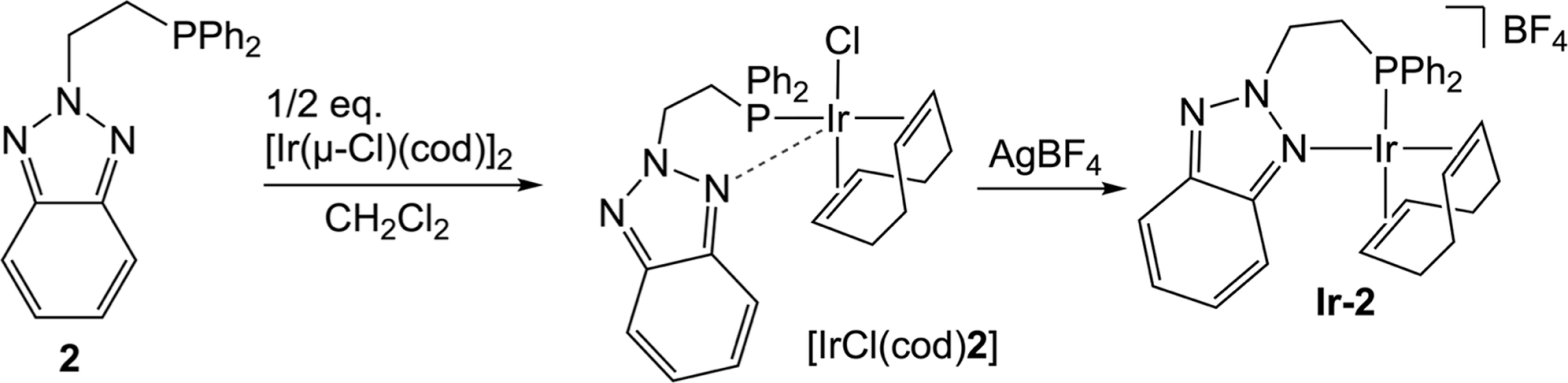
Synthesis of Complex **Ir-2**

The coordination of the phosphane to the metal center was confirmed by the shift of the phosphorus nucleus signal in the ^31^P­{^1^H} NMR spectrum, from δ −21.0 ppm in **2** to 11.4 ppm in [IrCl­(cod)**L2**]. Chloride abstraction with AgBF_4_ resulted in a downfield shift of the phosphane signal to δ 16.3 ppm for **Ir-2** in the ^31^P­{^1^H} NMR spectrum. A similar pattern to that of **Ir-1** was observed in the ^1^H NMR spectrum of **Ir-2**, which indicates the bidentate coordination of **2** in this case as well. The signals of the CH_2_P protons appear as a doublet of triplets at δ 3.07 ppm (^2^ *J*_P–H_ = 6.9 Hz, ^3^ *J*_H–H_ = 5.0 Hz), while those corresponding to the NCH_2_ protons split into two doublets of doublets at δ 5.77 ppm (^2^ *J*_H–H_ = 6.9 Hz, ^3^ *J*_H–H_ = 5.0 Hz) and 5.72 ppm (^2^ *J*_H–H_ = 7.0 Hz, ^3^ *J*_H–H_ = 5.0 Hz), each integrating 1H.

For comparison, we also prepared the related complex **Ir-3** from the analogous P–N ligand **3**, previously reported by us.^52^ This resulted in the formation of a 5-membered chelate ring—unlike the six-membered rings formed upon coordination of ligands **1** or **2**. The synthetic protocol is analogous to that described above with ligands **1** and **2** (Scheme 6).

**Scheme 6:**
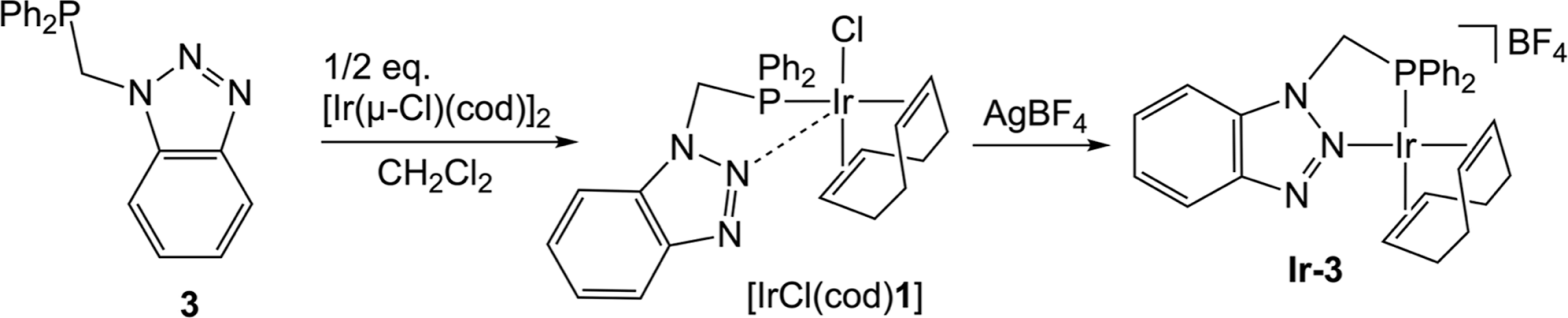
Synthesis of Complex **Ir-3**

The most representative signal in the ^1^H NMR spectrum is a doublet at δ 5.34 ppm, with a ^2^ *J*_H–P_ coupling constant of 5.33 Hz, attributed to the methylene protons. The observation of a single resonance for this group indicates that the two protons are chemically equivalent, consistent with a symmetric square planar geometry around the metal center. The ^31^P­{^1^H} NMR spectrum shows a singlet at δ 38.2 ppm.

### Synthesis and Characterization of **Rh­(I)** Complexes

The Rh­(I) complexes **Rh-1** ([Rh­(cod)­(κ^2^-*P*,*N*-**1**]) and **Rh-2** ([Rh­(cod)­(κ^2^-*P*,*N*-**2**]) were prepared analogously to their Ir counterparts from reactions of [{Rh­(μ-Cl)­(cod)}_2_] with **1** and **2** in a 1:2 molar ratio, respectively. The ^1^H NMR spectra of both Rh complexes indicate the bidentate coordination of **1** and **2** as evidenced by the appearance of diastereotopic protons in the NCH_2_ group. For **Rh-1**, these signals appear as multiplets at δ 5.43–5.38 and 5.37–5.33 ppm, each integrating to 1H. For **Rh-2**, they emerge at δ 5.96–5.91 and 5.91–5.51 ppm, also integrating for 1H each. The ^31^P­{^1^H} NMR spectra of **Rh-1** and **Rh-2** show one doublet each, at δ 22.3 and 29.0 ppm, with coupling constants P–Rh of 147.7 and 143.9 Hz, respectively.

Single crystals of **Rh-1** suitable for single-crystal X-ray diffraction analysis were obtained from slow evaporation of a concentrated solution **Rh-1** in dichloromethane. The crystallographic data obtained for **Rh-1** are similar to those already described for **Ir-1**. **Rh-1** presents a slightly distorted square-planar geometry, with the ligands cod and **1** coordinating in a bidentate manner (Figure 4). The metallacycle Rh–N(1)–N(2)–C(10)–C(11)–P adopts a boat conformation with the Rh and C(10) atoms placed at out-of-plane positions. Also, **1** exhibits a κ^2^ *P*,*N* coordination with an N(1)–Rh–P bite angle of 85.85(4)° and bond lengths N(1)-Rh and Rh–P of 2.0832(13) and 2.2951(4) Å, respectively. The benzo-1,2,3-triazole ring is out of the coordination plane, with a N(9)–N(1)–Rh–P torsion angle of 44.25(11)°. As for the Rh-ct(1), Rh-ct(2), C(25)–C(26), and C(29)–C(30) distances, similar to **Ir-1**, in **Rh-1** the trans influence of the phosphane group makes the distance Rh-ct(2) longer than Rh-ct(1) and the olefin bond length C(25)–C(26) shorter than C(29)–C(30). Interestingly, the bond lengths N(1)–N(2) [1.3560(17) Å] and N(1)–N(9) [1.3101(18) Å], along with the planarity of the nitrogen atom N(1) [Σ°_N(1)_ = 359.13(19)°], indicate a greater contribution from the neutral resonance structure **I** and an sp^2^ hybridization of N(1). The triazole ring shows pitch and yaw angles of θ 8.9° and ψ 1.5°, respectively, similar to those described for **Ir-1**.

**Figure 4:**
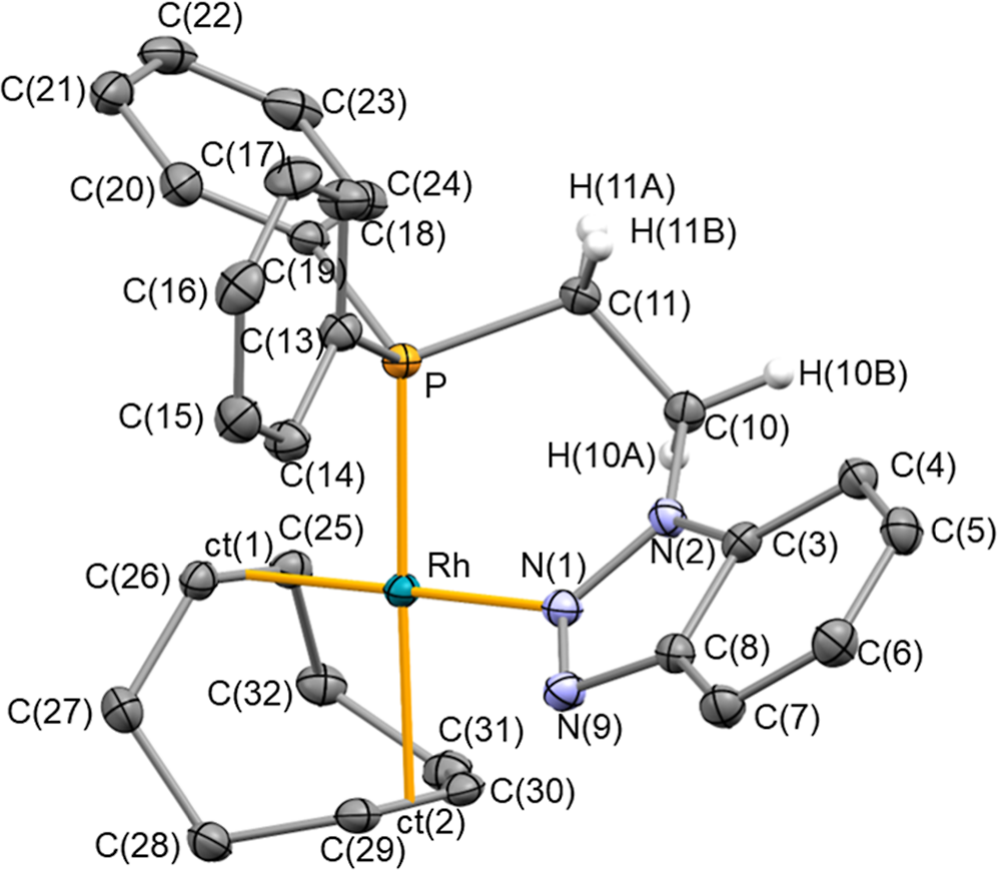
ORTEP view of **Rh-1** with 50% probability ellipsoids. BF4^–^ counterions and nonrelevant hydrogen atoms have been omitted. Selected bond lengths (Å) and angles (deg): N(1)–N(9) 1.3101(18), N(1)–N(2) 1.3560(17), N(1)-Rh 2.0832(13), Rh–P 2.2951(4), Rh-ct(1) 2.02983(13), Rh-ct(2) 2.11826(15), C(25)–C(26) 1.396(2), C(29)–C(30) 1.377(2); N(9)–N(1)–N(2) 110.61(12), N(9)–N(1)–Rh 125.73(10), N(2)–N(1)–Rh 122.79(9), N(1)–N(2)–C(3) 108.95(12), N(1)–N(2)–C(10) 120.61(12), C(3)–N(2)–C(10) 130.41(13), C(11)–P-Rh 109.31(5), N(1)–Rh–P 85.85(4), C(10)–C(11)–P 111.85(10), N(2)–C(10)–C(11) 111.86­(12), ct(1)-Rh-ct(2) 87.149(5), ct(1)-Rh–N(1) 179.17(4). ct(1) and ct(2) are the centroids of C(25) and C(26), and C(29) and C(30), respectively.

**Scheme 7:**
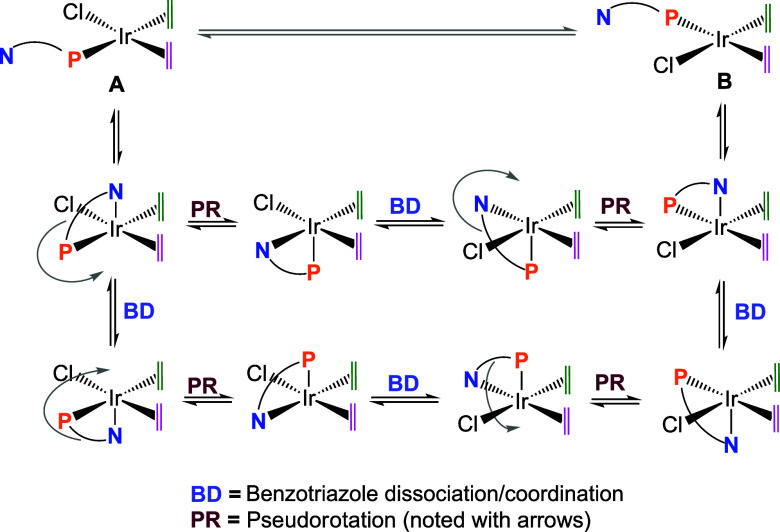
Proposed Isomerization Equilibrium for [IrCl­(cod)­(PN)]^a^

a Ligand structures and cod have been simplified for clarity.

The crystal structure of **Rh-2** also confirms the proposed κ^2^ *P*,*N* coordination of **2** to the rhodium center (Figure 5). **Rh-2** presents a distorted square-planar geometry, with **2** presenting an N(1)–Rh–P bite angle of 85.20(5)°, and Rh–P and Rh–N(1) bond distances of 2.2837(7) and 2.1166(19) Å, respectively. The two cod olefin groups show Rh-ct(1), Rh-ct(2), C(25)–C(26), and C(29)–C(30) bond distances of 2.0206(3), 2.1212(3), 1.389(3), and 1.367(4) Å, respectively, where ct(2) is the centroid of C(29) and C(30), which is the olefin group *trans* to the phosphane moiety. The bond lengths N(1)–N(2) and N(2)–N(3) of 1.340(3) and 1.316(3) Å suggest a greater contribution from the equivalent resonance structures **V** and **VI**, where charge separation maintains the C_6_-ring’s aromaticity. Furthermore, the planarity of the nitrogen atom N(1) [Σ°_N(1)_ = 360.0(3)°] suggest an sp^2^ hybridization for N(1). The conformation adopted by the chelate ring Rh–N(1)–N(2)–C(10)–C(11)–P is boat-like, with the Rh and C(10) atoms at the out-of-plane positions. The triazole ring is out of the coordination plane, adopting the most favorable disposition to minimize chelate ring tensions [C(9)–N(1)–Rh–P −53.35(16)°]. The different linkage in **2** relative to **1** likely accounts for the markedly different pitch (θ = 0.9°) and yaw (ψ = 7.5°) angles observed in **Rh-2** compared with **Rh-1** (vide infra).

**Figure 5:**
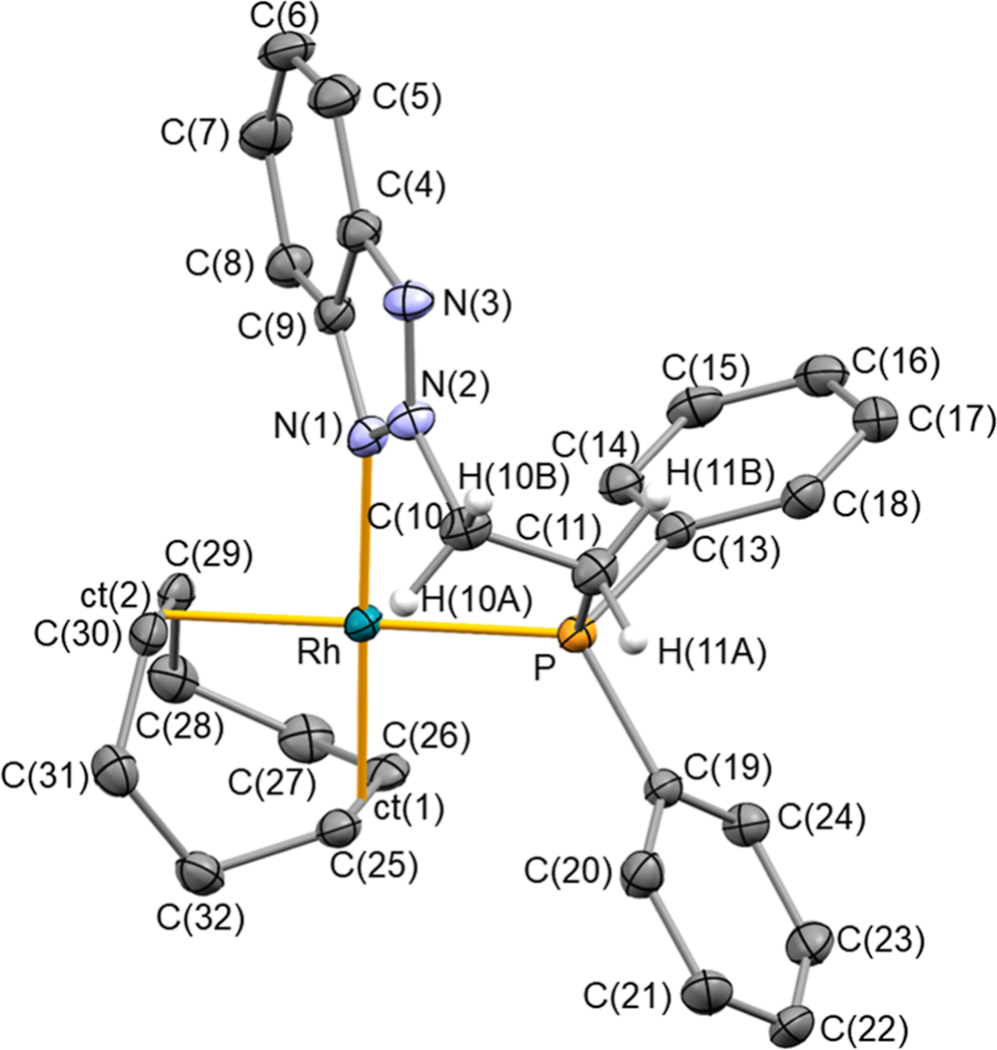
ORTEP view of **Rh-2** in **Rh-2**·2C_4_H_10_O with 50% probability ellipsoids. BF_4_^–^ counterions and most hydrogen atoms have been omitted for clarity. Selected bond lengths (Å) and angles (deg): Rh–P 2.2837(7), Rh–N(1) 2.1166(19), Rh-ct(1) 2.0206(3), Rh-ct(2) 2.1212(3), C(25)–C(26) 1.389(3), C(29)–C(30) 1.367(4), N(2)–N(3) 1.316(3), N(1)–N(2) 1.340(3); N(1)–Rh–P 85.20(5), ct(1)–Rh–N(1) 178.80(6), ct(1)-Rh-ct(2) 86.542(7), N(2)–N(1)–C(9) 103.75(18), C(9)–N(1)–Rh 135.62(16), N(2)–N(1)–Rh 120.63(14), C(11)–P-Rh 108.59(8), N(3)–N(2)–N(1) 116.35(19), N(3)–N(2)–C(10) 122.10(19), N(1)–N(2)–C(10) 121.43(19), N(2)–C(10)–C(11) 110.64­(19), C(10)–C(11)–P(12) 112.04(17). ct(1) and ct(2) are the centroids of C(25) and C(26), and C(29) and C(30), respectively.

The crystal structures of **Rh-1** and **Rh-2**—analogous to each other except for the P–N ligand—allow for the evaluation of the differences derived from the coordination of ligands **1** and **2**. The Rh–N bond lengths of **Rh-1** and **Rh-2** are 2.0832(13), and 2.1160(19) Å, respectively, which contrasts with the expected higher Lewis basicity of N1 in **2** (**C**-type structure), compared to N2 in **1** (**B**-type structure). The shorter Rh–N bond length for **Rh-1** could be explained by considering the orientation of the benzo-1,2,3-triazole ring in both complexes. On one hand, in the case of **Rh-1**, the ring is relatively far from the metal center, minimizing steric repulsions with the cod ligand. On the other hand, for **Rh-2**, coordination through N(1) brings the ring closer to the metal center and, consequently, to the olefin group *cis* to the triazole (Figure 5). On these grounds, the orientation of the benzo-1,2,3-triazole ring in **Rh-2** prevents a closer approach of the N(1) atom to the metal center due to steric hindrance, thus bringing about an elongated Rh–N bond length. Accordingly, the increased yaw angle of the triazole in **Rh-2** should allow the benzo-1,2,3-triazole to move away from the metal center and the cod ligand.

### Fluxional Behavior of [IrCl­(cod)­(PN)] Complexes as a Probe for Assessing Ligand Hemilability

The intermediate complex [IrCl­(cod)**1**] was studied using variable-temperature ^1^H NMR experiments (Figure S50), since the absence of signals corresponding to the cod olefin protons in the ^1^H NMR spectrum of this complex at room temperature suggested a fluxional behavior. At temperatures below 233 K, two broad multiplets centered at δ 5.13 and 2.63 ppm emerge, integrating 2H each, which correspond to the olefinic protons of the cod ligand. As the temperature decreases, the chemical shifts of the methylene proton signals undergo slight variations. However, the most notable change is observed in one of the signals attributed to the aliphatic protons of the cod ligand centered at δ 1.77 ppm, which originally integrates for 4H. This signal splits into two distinct peaks at δ 1.90 and 1.60 ppm, each integrating to 2H.

This fluxional behavior suggests that coordination of the triazole to the metal center initiates an equilibrium in which the phosphane can exchange positions by a sequence of coordination-dissociation and pseudorotation steps, ultimately swapping the olefin *trans* to it (**A** ⇌ **B**) (Scheme 7). As a result, the olefinic protons of the cod ligand become equivalent in the fast-exchange regime of the ^1^H NMR spectrum at elevated temperatures. However, this was not observed due to the low boiling point of CD_2_Cl_2_ (Figure S50).

The activation Gibbs energy (Δ*G* ^‡^) for this fluxional process was determined by variable-temperature ^1^H NMR spectroscopy. The calculation was based on the resonances at δ 1.90 and 1.60 ppm, corresponding to the aliphatic protons of the cod ligand, which show a coalescence temperature of 253 K, yielding a Δ*G* ^‡^ of 10.24 kcal·mol^–1^ (see Section 4 of the Supporting Information).

Similarly to the [IrCl­(cod)**1**] complex, the ^1^H NMR spectrum of [IrCl­(cod)**2**] does not show signals corresponding to the olefinic protons of the cod ligand at room temperature. Low temperature ^1^H NMR reveals an analogous behavior to that described above for [IrCl­(cod)**1**], with two broad resonances appearing at δ 5.08 and 2.56 ppm at 183 K that correspond to the olefinic protons of the cod ligand (Figure S51).

The Δ*G* ^‡^ calculated for the fluxional process of [IrCl­(cod)**2**] was determined employing the resonances at δ 1.87 and 1.57 ppm, corresponding to the olefinic protons of the cod ligand, which coalesce at 243 K, resulting in Δ*G* ^‡^ = 10.60 kcal·mol^–1^.

In contrast to [IrCl­(cod)**1**] and [IrCl­(cod)**2**], [IrCl­(cod)**3**] displays a single resonance at δ 3.92 ppm, attributed to the olefinic protons of the cod ligand, which become equivalent due to the fluxional process described above (Figure S52). Lowering the temperature to 183 K was not sufficient to reach coalescence. Consequently, line shape analysis was employed to estimate the maximum activation barrier, indicating that Δ*G* ^‡^ must be below 8.87 kcal·mol^–1^.

Activation Gibbs energies (Δ*G* ^‡^) of 10.24 and 10.60 kcal·mol^–1^ were determined for [IrCl­(cod)**1**] and [IrCl­(cod)**2**], respectively; while a maximum activation Gibbs energy (Δ*G* ^‡^) of 8.87 kcal·mol^–1^ was obtained for [IrCl­(cod)**3**]. According to the proposed fluxional process, which is prompted by the coordinating ability of the triazole moiety in ligands **1**–**3**, a lower activation barrier indicates a more strongly coordinating triazole, and vice versa. Accordingly, the triazole donor strength in this type of complexes follows the order: **3** > **1** > **2**, although the difference between **1** and **2** is small, with **3** being significantly more strongly coordinating than the other two ligands.

### Computational Study of the Electronic Structure and Bonding

In order to gain insight into the nature of the bond between the N atoms in both isomeric triazoles and the metal center, computational studies based on calculations of the electron localization function (ELF) were carried out. This function provides an image of the chemical bond in real space in terms of Lewis structures. This allows for the identification of chemically meaningful regions in the real space, such as the lone pairs of nitrogen atoms in the triazole molecules, which are associated with the ELF basins.^58^ The electron population enclosed within each ELF basin can be computed by integrating the electron density gathered exclusively in it. Indeed, the Lewis basicity of the N atoms that facilitate the formation of chelate rings with the metal, N1, was evaluated attending to the population of the key ELF basins (see Figure 6). In **1**, the lone pair of N1, represented by the ELF basin V­(N1), contains 3.04 e^–^; which is slightly higher than the population of V­(N9), 2.99 e^–^. Although the difference between the population of V­(N1) and V­(N9) is small, the picture agrees with the proposed Lewis basicity based on resonance structures of Scheme 2.

**Figure 6:**
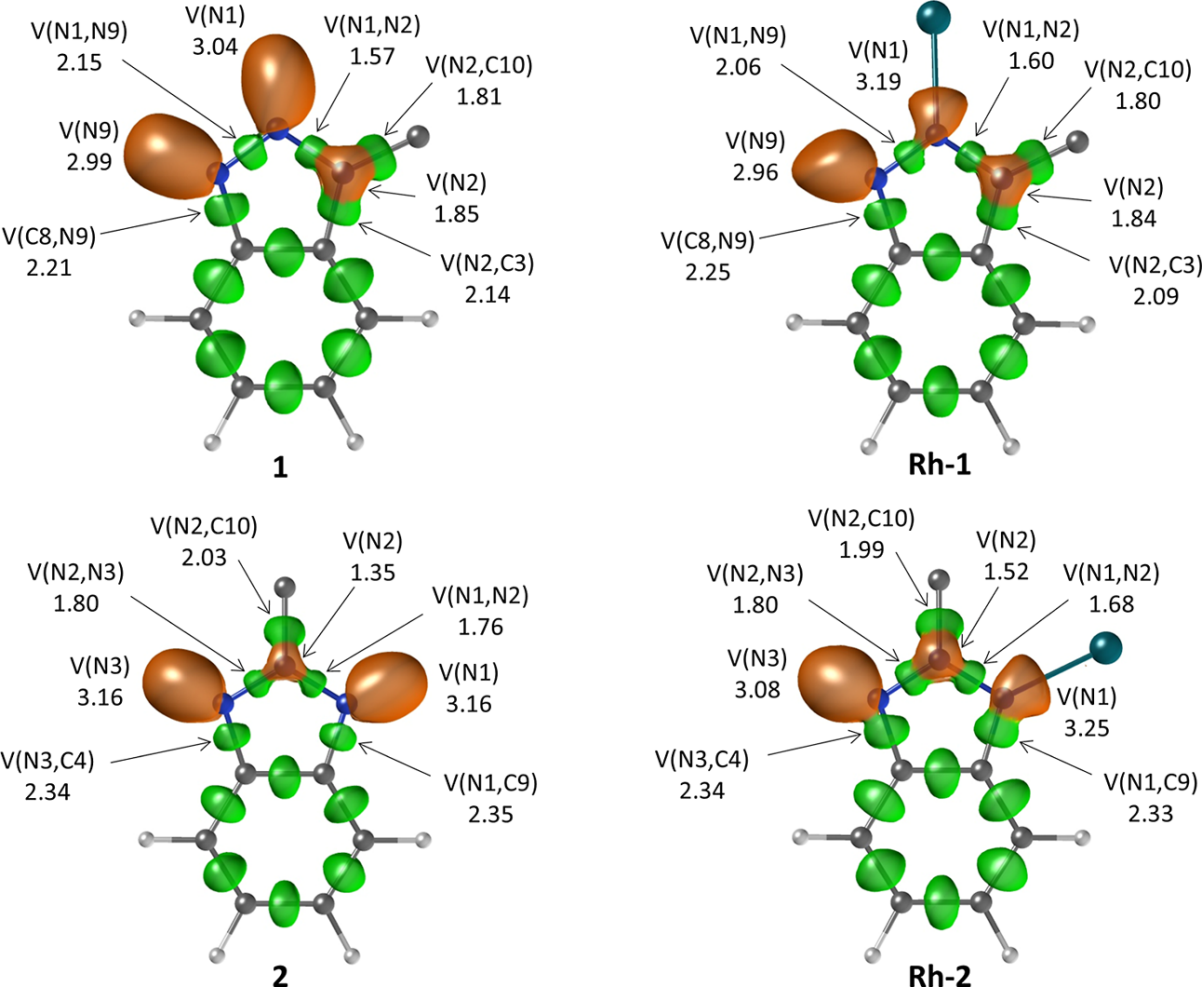
Representation of the ELF structures for **1**, **2**, **Rh-1**, and **Rh-2**. For clarity, the 2-(diphenylphosphanyl)­ethyl substituent of the triazole unit, as well as all other ligands not directly involved in the analysis, have been omitted. ELF basins are labeled according to the nuclei with which they are in contact. Thus, V­(N) basins correspond to the lone pairs on nitrogen atoms (shown in orange), while V­(N,N) and V­(N,C) basins correspond to N–N and N–C covalent bonds (shown in green). The basin populations—the electron density integrated over each ELF basin volume—are also provided (in electrons). For simplicity, basins associated with C–H bonds have been omitted.

The higher basic character of ligand **2** is revealed by the ELF analysis. Namely, the ELF population of basin V­(N1) is 3.16 e^–^, 0.12 e^–^ higher than that of the basin representing the N1 lone pair in **1**. Such differences maintain upon coordination to the metal center, as shown in Figure 6 right. In **Rh-1**, the lone pair of N1 has 3.19 e^–^, while in **Rh-2**, it bears a population of 3.25 e^–^. This analysis further supports the stronger electron-donating character of ligand **2** and suggests stronger coordination of such ligand to the Rh center in terms of covalent (electron-sharing) interactions.

Finally, it should be noted that the high electron delocalization character of ligands **1** and **2** facilitates electron transfers to the coordinated N atoms from the (partially) aromatic triazole-based ring. Although the above analysis focuses on the Rh-based complexes, similar conclusions apply to the Ir analogues, with only minimal variations in the basin populations, as shown in Tables S1 and S2.

The previous analysis was completed by the energetic picture provided by the interacting quantum atoms (IQA) energy decomposition scheme, which was applied to determine the strength of Rh–N bonds.^59^ Within this framework, the total energy of the system can be reconstructed from the individual atomic (or group) contributions. In turn, the interaction energy between two groups, *E*_int_, can be partitioned into a Coulomb, or classic, contribution (the ionic counterpart coming from the classic terms in the Hamiltonian), *V*_cl_; and an exchange–correlation interaction energy, which is related to the covalent counterpart of bonding, *V*_xc_.^60,61^ Moreover, the classical counterpart is known to be strongly influenced by the long-range nature of electrostatic interactions, which often cancel out overall but can significantly impact the interaction energy (*E*_int_) of individual pairs of atoms. In contrast, the exchange–correlation energy term, *V*_xc_, more accurately captures chemical bonding by reflecting the strength of interactions through electron pair sharing and will therefore be considered as the reference magnitude to characterize chemical bonding in this analysis.^62^

*V*_xc_ for the Rh–N interaction in **Rh-1** and **Rh-2** takes values of −86.1 and −83.2 kcal·mol^–1^, respectively. These energies, although similar, show a slightly greater strength for the Rh–N bond in **Rh-1**, contrary to the trend observed in the Lewis basicity of the N atoms involved in coordination. However, this trend aligns with crystallographic data, where the Rh–N bond distance is slightly shorter for **Rh-1**, which supports that the longer M–N bonds observed for **Rh-2** are, in broad strokes, attributable to steric effects. Moreover, the relative coordinating abilities of the triazole moieties in ligands **1** and **2** are consistent with the trend observed in the variable-temperature ^1^H NMR studies of the [IrCl­(cod)­(PN)] complexes and their corresponding Δ*G* ^‡^ values for the fluxional processes. Note that a similar effect is observed for the Ir-based complexes, where the Ir–N interaction is more favorable in **Ir-1** than in **Ir-2** by 4.4 kcal·mol^–1^, as shown in Tables S3 and S4.

### Catalytic Activity of Ir Complexes in the Dehydrogenation of FA

To evaluate how hemilability modulation in phosphane-triazole ligands influences catalytic performance, the activities of the corresponding iridium complexes (**Ir-1**, **Ir-2**, and **Ir-3**) were assessed employing the dehydrogenation of FA as model reaction. Preliminary studies with **Rh-1** showed lower activity than the related Ir complex; consequently, Rh complexes were not further investigated as catalysts for these reactions.

In this work, FADH was explored under two different sets of conditions, using 0.1 mol % of the catalyst at 80 °C: (i) in a 1:1 mixture HCOOH/H_2_O with a 30 mol % of HCOONa (Figure 7A), and (ii) in a neat 5:2 molar mixture HCOOH/Et_3_N (Figure 7B).

**Figure 7:**
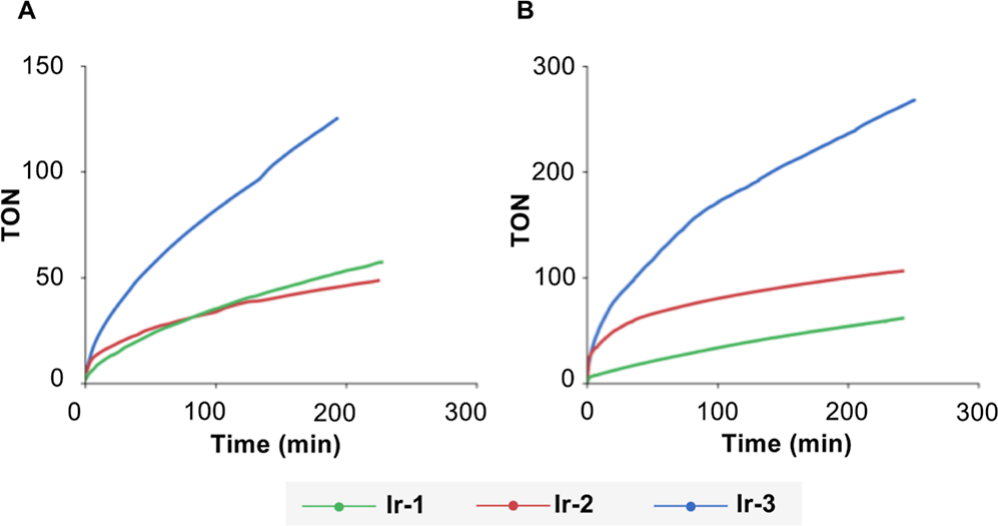
Plots TON vs time of FAD in a (**A**) 1:1 mixture HCOOH/H_2_O (volume); and (**B**) 5:2 molar mixture HCOOH/Et_3_N.

Complex **Ir-3**, featuring the most strongly coordinating ligand of the series (**3**), exhibits the highest catalytic activity, significantly outperforming **Ir-1** and **Ir-2** under both reaction conditions. Ligands **1** and **2** lead to comparable performances in HCOOH/H_2_O; however, in the HCOOH/Et_3_N mixture, **2** clearly outperforms **1**. This enhanced activity may be attributed to the greater ability of **2** to stabilize the unsaturated active species formed after cod hydrogenation, while also donating more electron density to the metal center. Note that the weaker Ir–N bond observed in **Ir-2** stems from steric repulsion between the cod olefin and the benzo-1,2,3-triazole moiety of ligand **2**. Consequently, in the absence of cod, the unsaturated active species are likely better stabilized by **2** than **1**. In fact, the most strongly coordinating ligand of the series, **3**, leads to the best overall performance.

The initial TOF values in the 1:1 mixture HCOOH/H_2_O were 310 (**Ir-3**), 292 (**Ir-2**) and 170 (**Ir-1**) h^–1^; while in the mixture HCOOH/Et_3_N they increase to 948 (**Ir-3**), 828 (**Ir-2**) and 186 (**Ir-1**) h^–1^ (Table 1). Thus, ligand **2** consistently leads to better performances than **1** at initial stages, with **3** showing the best performance. At longer reaction times, catalyst decomposition occurs for **Ir-1** and **Ir-2** more rapidly than for **Ir-3**, as suggested by the sharp decline of reaction rates as the reaction proceeds for **Ir-1** and **Ir-2**.

The reaction under optimized conditions with **Ir-3** catalyst (5:2 molar mixture of HCOOH/Et_3_N) reached a TON value of 444 after 420 min (Figure S53). Remarkably, after the initial decrease of activity described above, the slope of the reaction is maintained, showing that the catalyst remains active at long reaction times.

In literature examples, the most frequently employed reaction medium for FA dehydrogenation include: (a) aqueous solutions, (b) organic solvents, (c) neat FA, and (d) azeotropic mixtures of HCOOH and Et_3_N (typically in a 5:2 molar ratio). Among these, aqueous systems have yielded the highest TOFs, notably with the complex [IrCp*Cl­(2,2-bi-2-imidazoline)]­Cl reported by Li and co-workers, which achieved a TOF of 487,500 h^–1^ at pH 2.8,^63^ and the proton-responsive dinuclear Ir complex developed by Himeda and Fujita, operating in a 1 M HCOOH/HCOONa (1:1, pH 3.5) solution with a TOF of 228,000 h^–1^.^64^ Catalysts operating in organic solvents have also shown high activity, with examples including the Fe–PNP complex developed by Hazari and Schneider (TOF = 200,000 h^–1^),^25^ and the Ir­(III) catalyst featuring an *N*,*O*-pyridylideneamine ligand reported by Albrecht (TOF = 280,000 h^–1^).^16^

The use of solvent-free conditions has drawn growing interest due to the higher energy density such systems offer. Nevertheless, the highest TOFs reported under neat conditions remain comparatively low, typically not exceeding 10,000 h^–1^.^9,10,13,26,27^ In the case of azeotropic HCOOH/Et_3_N mixtures, more modest TOFs have been reported, such as 3630 h^–1^ at 40 °C with [RuCl_2_(dmso)_4_],^23^ and 8500 h^–1^ with the Mn complex [Mn­(CO)_2_(^*t*Bu^PNNOP)].^14^ However, it must be mentioned that, in contrast to the results we report here in Figure 7B, these systems employ organic cosolvents.

To detect possible reaction intermediates, complex **Ir-3** was treated with excess FA (10 equiv) in a Young NMR tube using CD_2_Cl_2_ as the solvent. **Pyridine** (4 equiv) was added to the reaction mixture to stabilize potential unsaturated species.

After 15 min at room temperature, two main species are formed, tentatively identified as a dihydride and a monohydride complex. The dihydride exhibits two doublets at δ −11.89 and −16.28 ppm, with H–P coupling constants of 19.3 and 12.5 Hz. The monohydride displays a doublet of doublets at δ −18.11, with an H–P and H–H coupling constants of 13.9 and 4.1 Hz, respectively. After 1 h, the dihydride species disappears, and the monohydride becomes the dominant species in solution. The ^1^H NMR data of this species is in good agreement with literature values^65^ and support the assignment of this complex as [IrH­(1-κ-4,5-η-C_8_H_13_)­(κ*-P*,*N*-**3**)­(py)]­[BF_4_], an intermediate formed during the hydrogenation of the cod ligand to cyclooctene (Figure S54).

The monohydride disappears after heating at 50 °C for 48 h, leading to the formation of cyclooctene (coe), produced by the partial hydrogenation of the cod ligand, as well as a new dihydride species, which becomes the main complex in solution. These findings are consistent with the formation of an unsaturated active species by cod hydrogenation under catalytic conditions. The ^1^H NMR presents two doublets of doublets at δ −18.28 and −20.49 ppm, with H–P coupling constants of 17.2 and 24.4 Hz, respectively, with an H–H coupling constant of 6.7 Hz (Figure S55). Moreover, two doublets of doublets that integrate 1H each can be attributed to the diastereotopic CH_2_P group of ligand **3** at δ 5.80 and 4.69 ppm, with H–P coupling constants of 10.6 and 3.4 Hz, respectively, and an H–H coupling constant of 14.3 Hz. The ^31^P­{^1^H} NMR shows a main peak at δ 28.4 ppm (Figure S56). Therefore, the NMR data suggests the formation of a dihydride species upon hydrogenation of the cod ligand. A tentative mechanism for the dehydrogenation of HCOOH and formation of the proposed hydride intermediates in this NMR experiment is depicted in Scheme 8. The catalytic cycle likely parallels those reported for related complexes that generate Ir dihydride intermediates upon hydrogenation of the cod ligand.^8,11^ The dehydrogenation mechanism would involve the protonation of one of the hydride ligands by HCOOH, thus rendering a molecule of H_2_ and a monohydride-formate complex. Subsequently, β-hydride elimination leads to the formation of CO_2_ and the regeneration of the dihydride. The observation that the dihydride complex is the major species in solution under the NMR conditions indicates that the protonation step is likely rate-limiting.

**Scheme 8:**
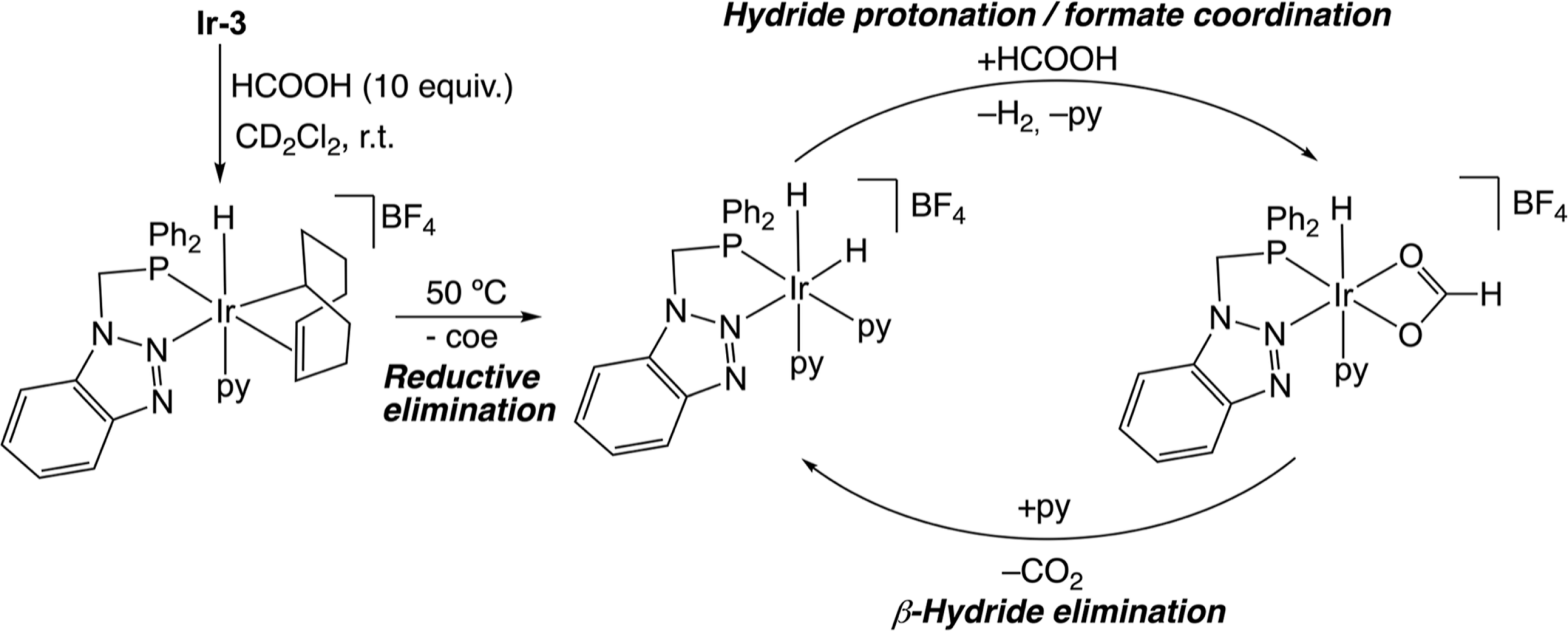
Mechanism Proposed for the Formation of the Dihydride Intermediate and HCOOH Dehydrogenation

The formation of a compound containing bridging hydrides can be detected in the ^1^H NMR spectrum as a minor product, exhibiting two doublets of doublets at δ −7.85 and −8.36 ppm, with H–P coupling constants of 24.1 and 24.5 Hz, respectively. The generation of dinuclear Ir complexes^8,66,67^ or small Ir clusters^68–71^ featuring bridging hydrides has been proposed as a deactivation pathway. Consequently, under catalytic conditions, the absence of pyridine—which stabilizes the dihydride intermediate observed in NMR experiments—likely promotes aggregation into less active dimeric species, leading to the gradual loss of catalytic activity. In fact, the ^1^H NMR spectra of samples taken over the course of the catalytic reaction (5:2 HCOOH/Et_3_N mixture, 0.1 mol % of **Ir-3**) show the formation of several resonances between δ −4 and −10 ppm, which plausibly correspond to bridging hydrides (Figure S57). A resonance at approximately δ −19 ppm is also observed and may be attributed to a monohydride species. These results suggest that, in the absence of excess pyridine (a coordinating ligand), aggregation occurs, which accounts for the loss of catalytic activity observed after the initial minutes of the reaction. Therefore, the conversion may be governed by several active species that form and transform as the reaction proceeds.

## Conclusions

In this study, we synthesized and characterized a family of hemilabile triazole-based P–N ligands. The coordination of these ligands with Ir and Rh complexes was studied by multinuclear NMR spectroscopy, single-crystal X-ray diffraction, and variable-temperature NMR studies, revealing consistent κ^2^-*P*,*N* chelation for the cationic complexes, and a fluxional behavior for the [IrCl­(cod)­(PN)] complexes triggered by the hemilabile nature of these ligands.

Notably, variable-temperature NMR data of the [IrCl­(cod)­(PN)] complexes indicate that ligand **3** presents the most strongly coordinating triazole, followed by ligands **1** and **2**. Complementary computational studies offered deeper insights into the electronic structure and bonding, revealing that although N1 in ligand **2** displays higher Lewis basicity, steric effects likely play a critical role in weakening the metal–nitrogen interaction relative to ligand **1**. This is supported by both experimental (bond lengths and activation barriers) and theoretical (ELF and IQA) results.

These findings highlight the subtle interplay between electronic and steric properties that govern the coordination behavior and dynamic properties of hemilabile ligands. The complementary nature of ligands **1**, **2**, and **3** suggests that they could be applied across a broad range of catalytic transformations by selecting the ligand that best matches the specific requirements of the catalytic system. Moreover, this study offers valuable insights for the rational design of hemilabile ligands, highlighting that the synthesis and evaluation of a single hemilabile ligand are insufficient to fully understand its catalytic impact, as even subtle modifications to electronic and steric properties can produce unexpected variations in performance.

**Table 1:** Overview of the Catalytic Reaction Results for **Ir-1**, **Ir-2** and **Ir-3**

	TOF values (h^–1^)	
catalyst (1 mol %)	5:2 molar HCOOH/Et_3_N	1:1 (v/v) HCOOH/H_2_O/30 mol % of HCOONa
**Ir-1**	186	170
**Ir-2**	828	292
**Ir-3**	948	310

## Experimental Section

All syntheses of ligands and organometallic complexes were carried out under an inert atmosphere using Schlenk techniques. The complexes were stored under an inert atmosphere in a Schlenk flask or in an MBraun drybox. Organic solvents were previously dried and distilled under argon or obtained from a solvent purification system (SPS) and collected under an inert atmosphere. All other starting materials were purchased from commercial suppliers and used without further purification. The complexes [Ir­(μ-Cl)­(cod)]_2_ ^72^ and [Rh­(μ-Cl)­(cod)]_2_ ^73^ were synthesized from [IrCl_3_] and [RhCl_3_] following procedures analogous to those described in the literature. Ligand **3** was synthesized following the procedure previously reported by us.^52^

The NMR spectra were recorded at 298 K on Bruker AVANCE 300 MHz, Bruker ARX 300 MHz, and Bruker AVANCE 400 MHz spectrometers. Chemical shifts (δ) are reported in ppm and referenced to the residual peaks of the deuterated solvents (^1^H and ^13^C). Coupling constants (*J*) are given in hertz (Hz). Spectral assignments were achieved through a combination of ^1^H–^1^H COSY, ^13^C­{^1^H} APT, ^1^H–^13^C HSQC, and ^1^H–^13^C HMBC experiments. High-resolution electrospray ionization mass spectra (HRMS) were acquired using a Bruker MicroTOF-Q quadrupole time-of-flight spectrometer.

### Safety Statement

No uncommon hazards are noted.

### Synthesis of 1-(2-Bromoethyl)-*1H*-benzo-1,2,3-triazole and 2-(2-bromoethyl)-*1H*-benzo-1,2,3-triazole

A mixture of benzotriazole (3.57 g, 30 mmol), K_2_CO_3_ (8.30 g, 60 mmol) and TBAI (3.20 g, 10 mmol) in 1,2-dibromoethane (15 mL) was heated at 80 °C for 16 h. After reaching room temperature, the mixture was diluted in dichloromethane (25 mL) and washed with water (3 × 20 mL). The organic phase, which contained the mixture of both isomers, was dried and the solvent was subsequently evaporated under reduced pressure. Pentane was then added and 1-(2-bromoethyl)-1*H*-benzo-1,2,3-triazole (*N*1), insoluble in pentane, was isolated and recrystallized from hot hexane, yielding the product as colorless crystals. The pentane solution was evaporated and 2-(2-bromoethyl)-1*H*-benzo-1,2,3-triazole (*N*2) was purified by recrystallization from a concentrated solution of *N*2 in hot pentane. The overall yield was 89%, with *N*1/*N*2 ratio of 8:2 (*N*1: 71%, 21.36 mmol, 4.8 g; *N*2: 18%, 5.34 mmol, 1.2 g). The NMR spectra and HRMS (ESI) of both isomers correspond to those described in the literature.^74^ *N* 1: 1H NMR (CDCl3, 400 MHz): δ 8.01–8.04 (m, 1H, C*H*Ar), 7.65–7.49 (m, 2H, C*H*Ar), 7.46–7.34 (m, 2H, C*H*_Ar_), 5.03 (t, ^3^ *J*_H–H_ = 6.6, 2H, C*H*_2_Br), 3.88 (t, ^3^ *J*_H–H_ = 6.6, 2H, C*H*_2_Br). *N* 2: ^1^H NMR (CDCl_3_, 400 MHz): δ 7.95–7.80 (m, 2H, C*H*_Ar_), 7.47–7.37 (m, 2H, C*H*_Ar_), 5.11 (t, ^3^ *J*_H–H_ = 6.7, 2H, NC*H*_2_), 3.98 (t, ^3^ *J*_H–H_ = 6.7, 2H, C*H*_2_Br).

### Synthesis of 1-[2-(Diphenylphosphanyl)­ethyl]-1*H*-benzo-1,2,3-triazole (**1**)

*t*BuOK (338 mg, 3.00 mmol) was added to a solution of HPPh_2_ (524 μL, 3.00 mmol) in dry THF (10 mL) at −78 °C, and the mixture was stirred for 15 min. The cooling bath was then removed, and the reaction was allowed to stir for an additional 2 h. The reddish solution was added dropwise via cannula to another solution of 1-(2-bromoethyl)-1*H*-benzo-1,2,3-triazole (678 mg, 3.00 mmol) in dry thf (10 mL). The mixture was stirred for 24 h at room temperature. The solvent was evaporated under reduced pressure, and the resulting precipitate was dissolved in dichloromethane and filtered through Celite. The filtrate was evaporated under reduced pressure, and the resulting oil washed with diethyl ether (3 × 15 mL) and dried in vacuo, yielding **1** as a white solid in 77% yield (766 mg, 2.31 mmol). ^1^H NMR (CDCl_3_, 400 MHz): δ 8.07–7.97 (m, 1H, C*H*_Ar_), 7.49–7.41 (m, 5H, PC*H*_Ar_), 7.39–7.28 (m, 8H, C*H*_Ar_ + PC*H*_Ar_), 4.78–4.68 (m, 2H, NC*H*_2_), 2.82–2.73 (m, 2H, C*H*_2_P). ^13^C­{^1^H} NMR APT, ^1^H–^13^C HSQC, ^1^H–^13^C HMBC (CDCl_3_, 101 MHz): δ 146.1 (s, *C*_*ipso*_N), 136.9 (d, ^1^ *J*_P–C_ = 12.0, *C*_*ipso*_P), 132.8 (d, ^2^ *J*_P–C_ = 19.2, *C*H_Ar‑*ortho*_P), 132.8 (s, *C*_*ipso*_N), 129.3 (s, *C*H_Ar‑*para*_P), 128.9 (d, ^3^ *J*_P–C_ = 7.0, *C*H_Ar‑*meta*_P), 127.4 (s, *C*H_Ar_N), 124.0 (s, *C*H_Ar_N), 120.2 (s, *C*H_Ar_N), 109.3 (s, *C*H_Ar_N), 45.5 (d, ^2^ *J*_P–C_ = 25.6, N*C*H_2_), 29.1 (d, ^1^ *J*_P–C_ = 15.5, *C*H_2_P). ^31^P­{^1^H} NMR (CDCl_3_, 162 MHz): δ −21.4 (s, *P*Ph_2_). HRMS (ESI): *m*/*z* calcd for [C_20_H_19_N_3_P]^+^, 332.1317; found, 332.1313.

### Synthesis of 2-[2-(Diphenylphosphanyl)­ethyl]-1*H*-benzo-1,2,3-triazole (**2**)

*t*BuOK (338 mg, 3.00 mmol) was added to a solution of HPPh_2_ (524 μL, 3.00 mmol) in dry THF (10 mL) at −78 °C, and the mixture was stirred for 15 min. The cooling bath was then removed, and the reaction was allowed to stir an additional 2 h. The reddish solution was added dropwise via cannula to another solution of 2-(2-bromoethyl)-1*H*-benzo-1,2,3-triazole (678 mg, 3.00 mmol) in dry thf (10 mL). The mixture was stirred for 24 h at room temperature. The solvent was evaporated under reduced pressure, and the resulting precipitate was dissolved in dichloromethane and filtered through Celite. The filtrate was evaporated under reduced pressure, and the resulting oil was washed with diethyl ether (3 × 15 mL) and dried in vacuo, yielding **2** as a white solid in 88% yield (825 mg, 2.49 mmol). ^1^H NMR (CDCl_3_, 400 MHz): δ 7.86–7.80 (m, 2H, C*H*_Ar_), 7.49–7.44 (m, 4H, PC*H*_Ar_), 7.36–7.31 (m, 8H, C*H*_Ar_ + PC*H*_Ar_), 4.87–4.78 (m, 2H, NC*H*_2_), 2.93–2.86 (m, 2H, C*H*_2_P). ^13^C­{^1^H} NMR APT, ^1^H–^13^C HSQC, ^1^H–^13^C HMBC (CDCl_3_, 101 MHz): δ 144.3 (s, *C*_*ipso*_N), 136.8 (d, ^1^ *J*_P–C_ = 12.0, *C*_*ipso*_P), 132.7 (d, ^2^ *J*_P–C_ = 19.2, *C*H_Ar‑*ortho*_P), 129.0 (s, *C*H_Ar‑*para*_P), 128.7 (d, ^3^ *J*_P–C_ = 6.8, *C*H_Ar‑*meta*_P), 126.3 (s, *C*H_Ar_N), 117.9 (s, *C*H_Ar_N), 53.9 (d, ^2^ *J*_P–C_ = 25.2, N*C*H_2_), 29.1 (d, ^1^ *J*_P–C_ = 15.7, *C*H_2_P). ^31^P­{^1^H} NMR (CDCl_3_, 162 MHz): δ −21.0 (s, *P*Ph_2_). HRMS (ESI): *m*/*z* calcd for [C_20_H_19_N_3_P]^+^, 332.1317; found, 332.1304.

### Synthesis of **Ir-1**, [Ir­(cod)(1)]­[BF_4_]

Complex [Ir­(μ-Cl)­(cod)]_2_ (76 mg, 0.11 mmol) was dissolved in dichloromethane (6 mL), and **1** (75 mg, 0.22 mmol) was added. The solution was stirred for 1 h at room temperature, after which AgBF_4_ (44 mg, 0.22 mmol) was added, and the mixture was stirred for 16 h protected from light. Subsequently, the mixture was filtered through Celite, the solvent was evaporated under reduced pressure, and the resulting oil was washed with hexane (3 × 10 mL), affording **Ir-1** as a yellow powder in 82% yield (129 mg, 0.18 mmol). ^1^H NMR (CD_2_Cl_2_, 400 MHz): δ 8.05–7.98 (m, 1H, C*H*_Ar_), 7.88–7.83 (m, 1H, C*H*_Ar_), 7.76–7.71 (m, 1H, C*H*_Ar_), 7.69–7.61 (m, 4H, PC*H*_Ar_), 7.59–7.56 (m, 1H, C*H*_Ar_), 7.55–5.46 (m, 6H, PC*H*_Ar_), 5.66 (br s, 2H, C*H*_COD_), 5.45–5.39 (m, 1H, NC*H*_2_), 5.38–5.34 (m, 1H, NC*H*_2_), 3.46 (br s, 2H, C*H*_COD_), 3.02–2.92 (m, 2H, C*H*_2_P), 2.48–2.32 (m, 4H, C*H*_2 COD_), 2.23–2.08 (m, 4H, C*H*_2 COD_). ^13^C­{^1^H} NMR APT, ^1^H–^13^C HSQC, ^1^H–^13^C HMBC (CD_2_Cl_2_, 101 MHz): δ 145.7 (s, *C*_*ipso*_N), 134.3 (s, *C*_*ipso*_N), 133.8 (d, ^2^ *J*_P–C_ = 11.1, *C*H_Ar‑*ortho*_P), 132.3 (d, ^4^ *J*_P–C_ = 2.5, *C*H_Ar‑*para*_P), 131.9 (s, *C*H_Ar_N), 130.0 (d, ^1^ *J*_P–C_ = 54.5, *C*_*ipso*_P), 129.7 (d, ^3^ *J*_P–C_ = 10.7, *C*H_Ar‑*meta*_P), 127.6 (s, *C*H_Ar_N), 120.0 (s, *C*H_Ar_N), 111.2 (s, *C*H_Ar_N), 99.5 (d, ^2^ *J*_P–C_ = 11.2, *C*H_COD_), 68.0 (s, *C*H_COD_), 48.1 (d, ^2^ *J*_P–C_ = 2.9, N*C*H_2_), 33.2 (br s, *C*H_2 COD_), 29.6 (br s, *C*H_2 COD_), 25.9 (d, ^1^ *J*_P–C_ = 34.2, *C*H_2_P). ^31^P­{^1^H} NMR (CD_2_Cl_2_, 162 MHz): δ 7.8 (s, *P*Ph_2_). ^19^F NMR (CD_2_Cl_2_, 376 MHz): δ −152.3 (s, B*F*_4_). HRMS (ESI): *m*/*z* calcd for [C_28_H_30_IrN_3_P]^+^, 632.1807; found, 632.1779.

### Synthesis of **Ir-2**, [Ir­(cod)(2)]­[BF_4_]

Complex [Ir­(μ-Cl)­(cod)]_2_ (76 mg, 0.11 mmol) was dissolved in dichloromethane (6 mL), and **2** (75 mg, 0.22 mmol) was added. The solution was stirred for 1 h at room temperature, after which AgBF_4_ (44 mg, 0.22 mmol) was added, and the mixture was stirred for 16 h protected from light. The mixture was then filtered through Celite, the solvent was evaporated under reduced pressure, and the resulting oil was washed with pentane (3 × 10 mL), affording **Ir-2** as an orange powder in 77% yield (122 mg, 0.17 mmol). ^1^H NMR (CD_2_Cl_2_, 400 MHz): δ 8.04 (d, ^3^ *J*_H–H_ = 8.8, 1H, C*H*_Ar_), 7.88 (d, ^3^ *J*_H–H_ = 8.7, 1H, C*H*_Ar_), 7.67 (at, *J*_H–H_ = 7.8, 1H, C*H*_Ar_), 7.54–7.46 (m, 5H, PC*H*_Ar_), 7.42–7.35 (m, 5H, PC*H*_Ar_ + C*H*_Ar_), 5.77 (dd, ^2^ *J*_H–H_ = 6.9, ^3^ *J*_H–H_ = 5.0, 1H, NC*H*_2_), 5.72 (dd, ^2^ *J*_H–H_ = 7.0, ^3^ *J*_H–H_ = 5.0, 1H, NC*H*_2_), 5.20 (br s, 2H, C*H*_COD_), 3.71 (br s, 2H, C*H*_COD_), 3.07 (dt, ^2^ *J*_P–H_ = 6.9, ^3^ *J*_H–H_ = 5.0, 2H, C*H*_2_P), 2.50–2.39 (m, 4H, C*H*_2 COD_), 2.15–2.04 (m, 4H, C*H*_2 COD_). ^13^C­{^1^H} NMR APT, ^1^H–^13^C HSQC, ^1^H–^13^C HMBC (CD_2_Cl_2_, 101 MHz): δ 144.5 (s, *C*_*ipso*_N), 143.3 (s, *C*_*ipso*_N),133.7 (d, ^2^ *J*_P–C_ = 11.6, *C*H_Ar‑*ortho*_P), 132.2 (d, ^4^ *J*_P–C_ = 2.6, *C*H_Ar‑*para*_P), 131.0 (s, *C*H_Ar_N), 129.5 (d, ^3^ *J*_P–C_ = 11.0, *C*H_Ar‑*meta*_P), 128.8 (d, ^1^ *J*_P–C_ = 54.3, *C*_*ipso*_P), 128.7 (s, *C*H_Ar_N), 120.3 (s, *C*H_Ar_N), 115.8 (s, *C*H_Ar_N), 94.3 (d, ^2^ *J*_P–C_ = 11.6, *C*H_COD_), 68.6 (br s, *C*H_COD_), 57.8 (d, ^2^ *J*_P–C_ = 2.8, N*C*H_2_), 32.8 (br s, *C*H_2 COD_), 30.2 (br s, *C*H_2 COD_), 26.4 (d, ^1^ *J*_P–C_ = 33.2, *C*H_2_P). ^31^P­{^1^H} NMR (CD_2_Cl_2_, 162 MHz): δ 16.3 (s, *P*Ph_2_). ^19^F NMR (CD_2_Cl_2_, 376 MHz): δ −151.8 (s, B*F*_4_). HRMS (ESI): *m*/*z* calcd for [C_28_H_30_IrN_3_P]^+^, 632.1807; found, 632.1805.

### Synthesis of **Ir-3**, [Ir­(cod)(3)]­[BF_4_]

Complex [Ir­(μ-Cl)­(cod)]_2_ (76 mg, 0.11 mmol) was dissolved in dichloromethane (6 mL), and **3** (70 mg, 0.22 mmol) was added. The solution was stirred for 1 h at room temperature, after which AgBF_4_ (44 mg, 0.22 mmol) was added, and the mixture was stirred for 16 h protected from light. Subsequently, the mixture was filtered through Celite, the solvent was evaporated under reduced pressure, and the resulting oil was washed with hexane (3 × 10 mL), affording **Ir-3** as a red powder in del 79% yield (122 mg, 0.17 mmol). ^1^H NMR (CD_2_Cl_2_, 400 MHz): δ 8.12–8.05 (m, 1H, C*H*_Ar_), 7.98–7.92 (m, 1H, C*H*_Ar_), 7.83–7.73 (m, 5H, C*H*_Ar_ + PC*H*_Ar_), 7.64–7.54 (m, 7H, C*H*_Ar_ + PC*H*_Ar_), 5.88 (br s, 2H, C*H*_COD_), 5.30 (d, ^2^ *J*_P–H_ = 6.8, 2H, C*H*_2_P), 4.08 (br s, 2H, C*H*_COD_), 2.45–2.28 (m, 8H, C*H*_2 COD_). ^13^C­{^1^H} NMR APT, ^1^H–^13^C HSQC, ^1^H–^13^C HMBC (CD_2_Cl_2_, 101 MHz): δ 146.6 (s, *C*_*ipso*_N), 134.0 (d, ^2^ *J*_P–C_ = 12.4, *C*H_Ar‑*ortho*_P), 133.2 (d, ^4^ *J*_P–C_ = 2.5, *C*H_Ar‑*para*_P), 133.0 (s, *C*H_Ar_N), 130.2 (d, ^3^ *J*_P–C_ = 11.3, *C*H_Ar‑*meta*_P), 128.4 (s, *C*_*ipso*_N), 128.3 (s, *C*H_Ar_N), 126.9 (d, ^1^ *J*_P–C_ = 56.1, *C*_*ipso*_P), 120.5 (s, *C*H_Ar_N), 112.9 (s, *C*H_Ar_N), 100.5 (br s, *C*H_COD_), 69.9 (br s, *C*H_COD_), 49.0 (d, ^1^ *J*_P–C_ = 33.6, *C*H_2_P), 33.4 (br s, *C*H_2 COD_), 29.3 (br s, *C*H_2 COD_). ^31^P­{^1^H} NMR (CD_2_Cl_2_, 162 MHz): δ 38.3 (s, *P*Ph_2_). ^19^F NMR (CD_2_Cl_2_, 376 MHz): δ −151.2 (s, B*F*_4_). HRMS (ESI): *m*/*z* calcd for [C_27_H_29_IrN_3_P]^+^, 619.1728; found, 619.1752.

### Synthesis of **Rh-1**, [Rh­(cod)(1)]­[BF_4_]

Complex [Rh­(μ-Cl)­(cod)]_2_ (37 mg, 0.08 mmol) was dissolved in dichloromethane (6 mL), and **1** (50 mg, 0.16 mmol) was added. The solution was stirred for 1 h at room temperature, after which AgBF_4_ (31 mg, 0.16 mmol) was added, and the mixture was stirred for 16 h protected from light. The mixture was then filtered through Celite, the solvent was evaporated under reduced pressure, and the resulting oil was washed with hexane (3 × 10 mL), affording **Rh-1** as a yellow powder in 90% yield (91 mg, 0.14 mmol). ^1^H NMR (CD_2_Cl_2_, 400 MHz): δ 8.00–7.95 (m, 1H, C*H*_Ar_), 7.81–7.77 (m, 1H, C*H*_Ar_), 7.72–7.64 (m, 5H, PC*H*_Ar_), 7.54–7.45 (m, 7H, C*H*_Ar_ + PC*H*_Ar_), 5.93 (br s, 2H, C*H*_COD_), 5.43–5.38 (m, 1H, NC*H*_2_), 5.37–5.33 (m, 1H, NC*H*_2_), 3.78–3.68 (m, 2H, C*H*_COD_), 2.95–2.85 (m, 2H, C*H*_2_P), 2.65–2.49 (m, 4H, C*H*_2 COD_), 2.38–2.23 (m, 4H, C*H*_2 COD_). ^13^C­{^1^H} NMR APT, ^1^H–^13^C HSQC, ^1^H–^13^C HMBC (CD_2_Cl_2_, 101 MHz): δ 145.5 (s, *C*_*ipso*_N), 134.2 (s, *C*_*ipso*_N), 133.6 (d, ^2^ *J*_P–C_ = 11.4, *C*H_Ar‑*ortho*_P), 132.1 (d, ^4^ *J*_P–C_ = 2.4, *C*H_Ar‑*para*_P), 131.1 (s, *C*H_Ar_N), 130.4 (d, ^1^ *J*_P–C_ = 46.7, *C*_*ipso*_P), 129.7 (d, ^3^ *J*_P–C_ = 10.5, *C*H_Ar‑*meta*_P), 127.0 (s, *C*H_Ar_N), 119.8 (s, *C*H_Ar_N), 110.9 (s, *C*H_Ar_N), 109.6 (dd, ^1^ *J*_Rh–C_ = 9.6, ^2^ *J*_P–C_ = 6.8, *C*H_COD_), 81.8 (d, ^1^ *J*_Rh–C_ = 12.4, *C*H_COD_), 47.4 (d, ^2^ *J*_P–C_ = 4.0, N*C*H_2_), 32.7 (d, ^2^ *J*_Rh–C_ = 2.6, *C*H_2 COD_), 29.0 (d, ^2^ *J*_Rh–C_ = 1.3, *C*H_2 COD_), 27.3 (d, ^1^ *J*_P–C_ = 27.3, *C*H_2_P). ^31^P­{^1^H} NMR (CD_2_Cl_2_, 162 MHz): δ 22.3 (d, ^1^ *J*_Rh–P_ = 147.7, *P*Ph_2_). ^19^F NMR (CD_2_Cl_2_, 376 MHz): δ −152.4 (s, B*F*_4_). HRMS (ESI): *m*/*z* calcd for [C_28_H_30_N_3_PRh]^+^, 542.1232; found, 542.1220.

### Synthesis of **Rh-2**, [Rh­(cod)(2)]­[BF_4_]

Complex [Rh­(μ-Cl)­(cod)]_2_ (50 mg, 0.10 mmol) was dissolved in dichloromethane (6 mL), and **2** (67 mg, 0.20 mmol) was added. The solution was stirred for 1 h at room temperature, after which AgBF_4_ (40 mg, 0.20 mmol) was added, and the mixture was stirred for 16 h protected from light. Subsequently, the mixture was filtered through Celite, the solvent was evaporated under reduced pressure, and the resulting oil was washed with hexane (3 × 10 mL), affording **Rh-2** as a yellow powder in 86% yield (110 mg, 0.17 mmol). ^1^H NMR (CD_2_Cl_2_, 400 MHz): δ 8.04–7.93 (m, 1H, C*H*_Ar_), 7.91–7.81 (m, 1H, C*H*_Ar_), 7.67–7.60 (m, 1H, C*H*_Ar_), 7.54–7.47 (m, 5H, C*H*_Ar_ + PC*H*_Ar_), 7.40–7.31 (m, 6H, PC*H*_Ar_), 5.96–5.91 (m, 1H, NC*H*_2_), 5.91–5.51 (m, 1H, NC*H*_2_), 5.55 (br s, 2H, C*H*_COD_), 3.96 (br s, 2H, C*H*_COD_), 3.12–3.03 (m, 2H, C*H*_2_P), 2.71–2.59 (m, 4H, C*H*_2 COD_), 2.34–2.22 (m, 4H, C*H*_2 COD_). ^13^C­{^1^H} NMR APT, ^1^H–^13^C HSQC, ^1^H–^13^C HMBC (CD_2_Cl_2_, 101 MHz): δ 144.3 (s, *C*_*ipso*_N), 143.9 (s, *C*_*ipso*_N), 133.5 (d, ^2^ *J*_P–C_ = 12.0, *C*H_Ar‑*ortho*_P), 131.9 (d, ^4^ *J*_P–C_ = 2.6, *C*H_Ar‑*para*_P), 130.6 (s, *C*H_Ar_N), 129.5 (d, ^3^ *J*_P–C_ = 10.5, *C*H_Ar‑*meta*_P), 128.9 (d, ^1^ *J*_P–C_ = 46.7, *C*_*ipso*_P), 128.2 (s, *C*H_Ar_N), 120.0 (s, *C*H_Ar_N), 115.9 (s, *C*H_Ar_N), 106.0 (dd, ^1^ *J*_Rh–C_ = 10.0, ^2^ *J*_P–C_ = 6.9, *C*H_COD_), 81.7 (d, ^1^ *J*_Rh–C_ = 12.4, *C*H_COD_), 58.3 (br s, N*C*H_2_), 32.5 (br s, *C*H_2 COD_), 29.4 (br s, *C*H_2 COD_), 27.4 (d, ^1^ *J*_P–C_ = 27.3, *C*H_2_P). ^31^P­{^1^H} NMR (CD_2_Cl_2_, 162 MHz): δ 29.0 (d, ^1^ *J*_Rh–P_ = 143.9, *P*Ph_2_). ^19^F NMR (CD_2_Cl_2_, 376 MHz): δ −151.8 (s, B*F*_4_). HRMS (ESI): *m*/*z* calcd for [C_28_H_30_N_3_PRh]^+^, 542.1232; found, 542.1240.

### X-ray Diffraction Analysis

Single crystals of **2** and **Ir-1** were obtained by slow diffusion of hexane into a solution of **2** and **Ir-1** in dichloromethane. Single crystals of **Rh-1** were obtained by slow evaporation of a concentrated solution of **Rh-1** in dichloromethane. Single crystals of **Rh-2** were obtained by slow diffusion of acetonitrile into a solution of **Rh-2** in dichloromethane. X-ray diffraction data were collected at 100(2) K on a Bruker APEX DUO (**Rh-2**), APEX (**Ir-1** and **Rh-1**) and D8 VENTURE (**2**) diffractometers with graphite-monochromatic Mo Kα radiation (λ = 0.71073 Å) using ω-scans. Intensities were integrated and corrected for absorption effects with the SAINT-PLUS^75^ and SADABS,^76^ both included in the APEX3 (**Ir-1**, **Rh-1**, and **Rh-2**) and APEX4 (**2**) packages. The structures were solved by Patterson’s method with SHELXS-97 ^77^ and refined by full matrix least-squares on *F* ^2^ with SHELXL-2014 ^78^ under WinGX.^79^

### Computational Details

Geometry optimizations and wave function calculations were performed at the density functional theory (DFT) level, by means of the GGA hybrid B3LYP exchange–correlation functional;^80^ in conjunction with D3BJ empirical correction dispersion scheme,^81,82^ and def2-SVP basis set;^83^ as implemented in the Gaussian 16 software package.^84^

The ELF was obtained with the TopMod program^85^ using the corresponding wave functions calculated at the B3LYP-D3BJ/def2-SVP level on a three-dimensional grid of 150 points in each direction. The IQA analysis was performed using the AIMAll package.^86^

## Supplementary Material



## Notes

Experimental data, DFT calculations, NMR spectra and *x*, *y*, *z* coordinates (PDF)
