# Supplementary material for: Hemilability Modulation via Phosphane-Triazole Ligand Design: Impact on Catalytic Formic Acid Dehydrogenation
Source: Inorg Chem. 2025 Dec 11;64(50):24539–52. doi: 10.1021/acs.inorgchem.5c03962 (PMC12728932; doi:10.1021/acs.inorgchem.5c03962)
Supplement: Supplementary file 1 [file ic5c03962_si_001.pdf]

Supporting Information for

Hemilability Modulation via Phosphane-Triazole Ligand Design: Impact on Catalytic Formic Acid Dehydrogenation

Susana García-Abellán,‡ Andrea Pérez-García,‡ Daniel Barrena-Espés,§ Miguel A. Casado, ‡ Julen Munarriz,† Vincenzo Passarelli, ‡ Manuel Iglesias* ‡*

‡Instituto de Síntesis Química y Catálisis Homogénea (ISQCH), CSIC-Universidad de Zaragoza, C/Pedro Cerbuna 12 , Zaragoza 50009, Spain.

†Departamento de Química Física and Instituto de Biocomputación y Física de Sistemas Complejos (BIFI), Universidad de Zaragoza, Zaragoza 50009, Spain.

§Departamento de Química Física y Analítica, Universidad de Oviedo, Oviedo 33006, Spain.

E-mail: s.garcia@unizar.es (Susana García-Abellán); miglesia@unizar.es (Manuel Iglesias)

Table of contents

1. NMR Spectra **3**

NMR spectra of P-N ligands 3

NMR spectra of organometallic complexes of Ir(I) 10

NMR spectra of organometallic complexes of Rh(I) 20

4. Variable temperature ^{1}H NMR spectra of intermediate complexes [IrCl(cod)[PN]] and kinetic analysis using the Arrhenius plot **28**

Ir-1 28

Ir-2 30

Ir-3 32

5. General procedure for catalytic activity studies **33**

Catalysis in HCOOH/H_2O (1:1 v/v). 33

Catalysis in HCOOH/Et_3N (5:2 molar). 33

7 h experiment TON vs time for Ir-3 under optimal conditions 33

6. NMR spectra of stoichiometric experiments 34

7. In operando studies 37

8. X-ray diffraction analysis 38

9. Computational calculations 39

1. NMR Spectra

NMR spectra of P-N ligands

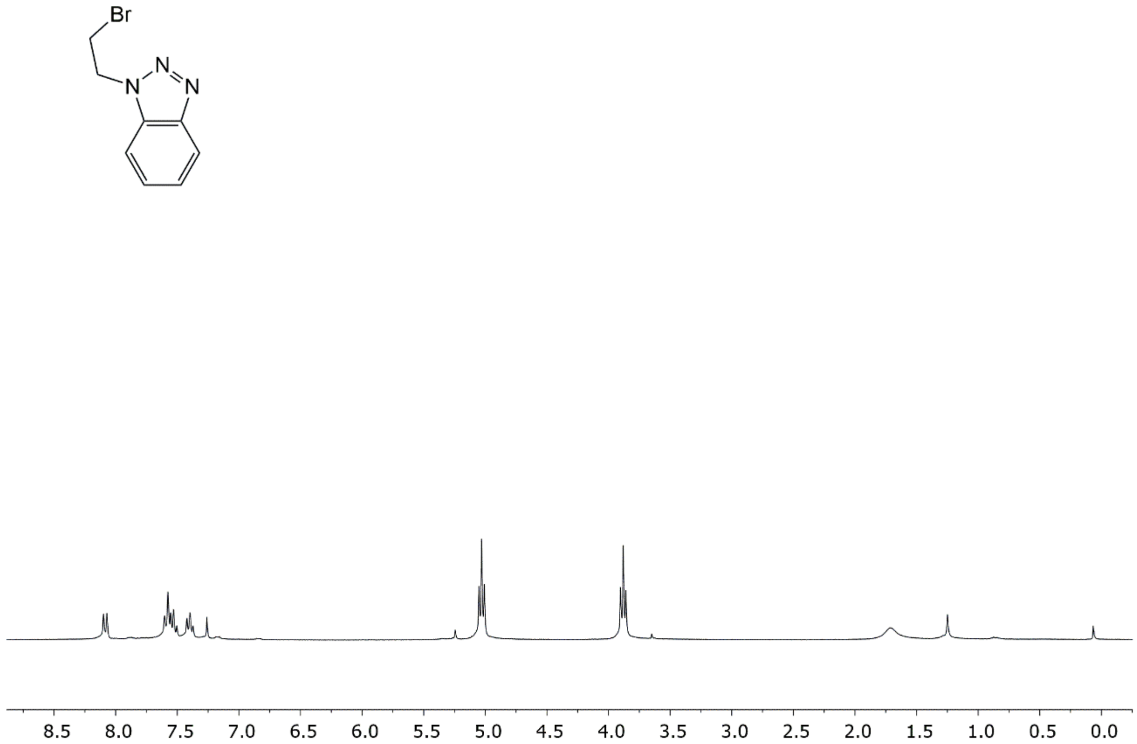

Figure S1. ^{1}H NMR spectrum of 1-(2-bromoetil)-*1H*-benzo-1,2,3-triazol in $CDCl_3$.

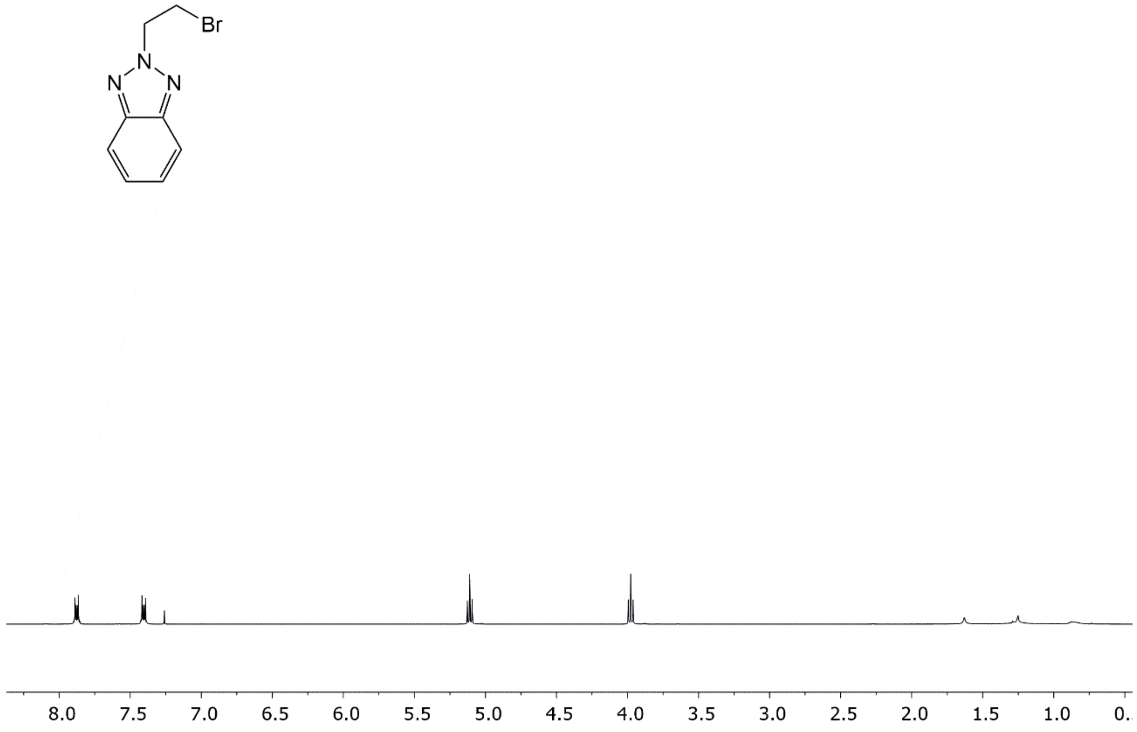

Figure S2. ^{1}H NMR spectrum of 2-(2-bromoetil)-*1H*-benzo-1,2,3-triazol in $CDCl_3$.

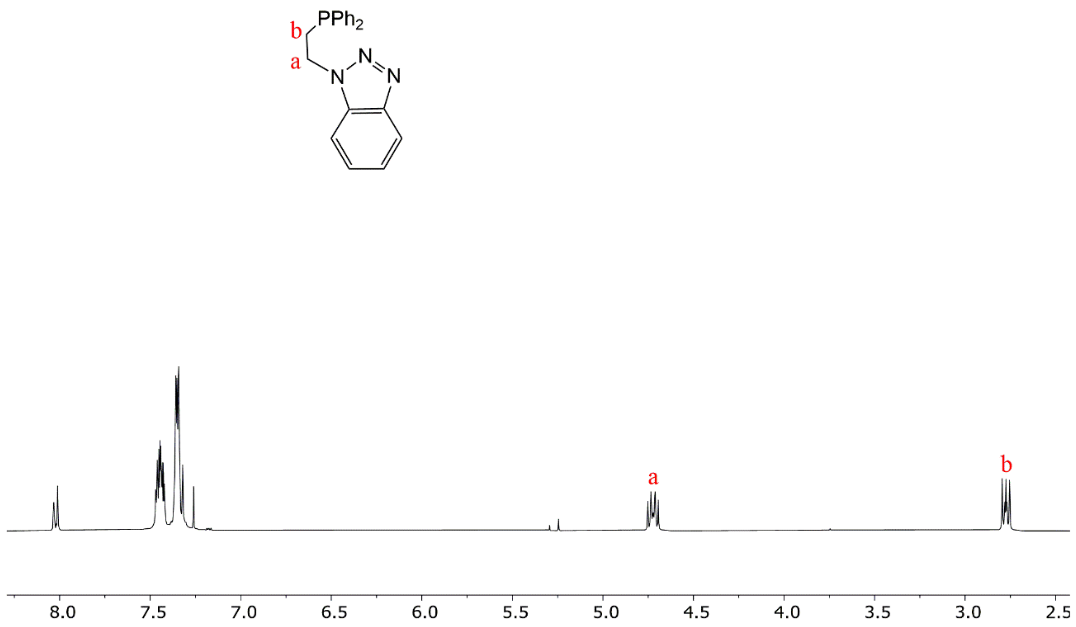

Figure S3. ^{1}H NMR spectrum of **1** in $CDCl_3$.

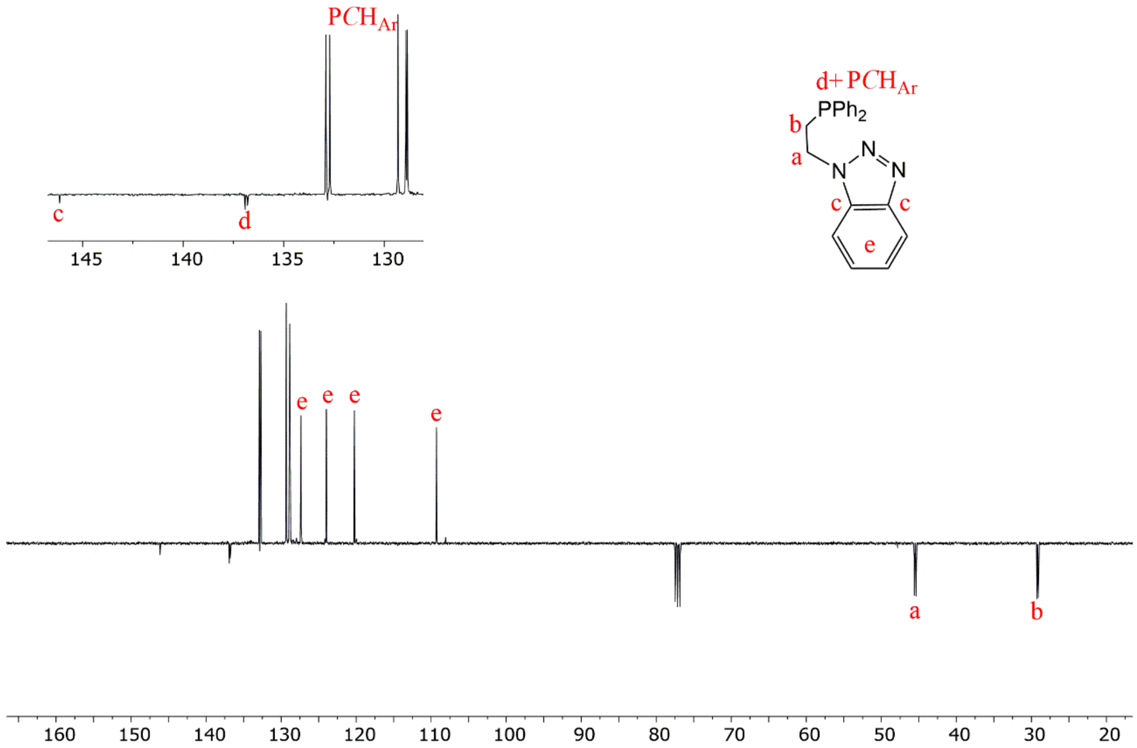

Figure S4. ^{13}C{^{1}H} NMR APT spectrum of **1** in $CDCl_3$.

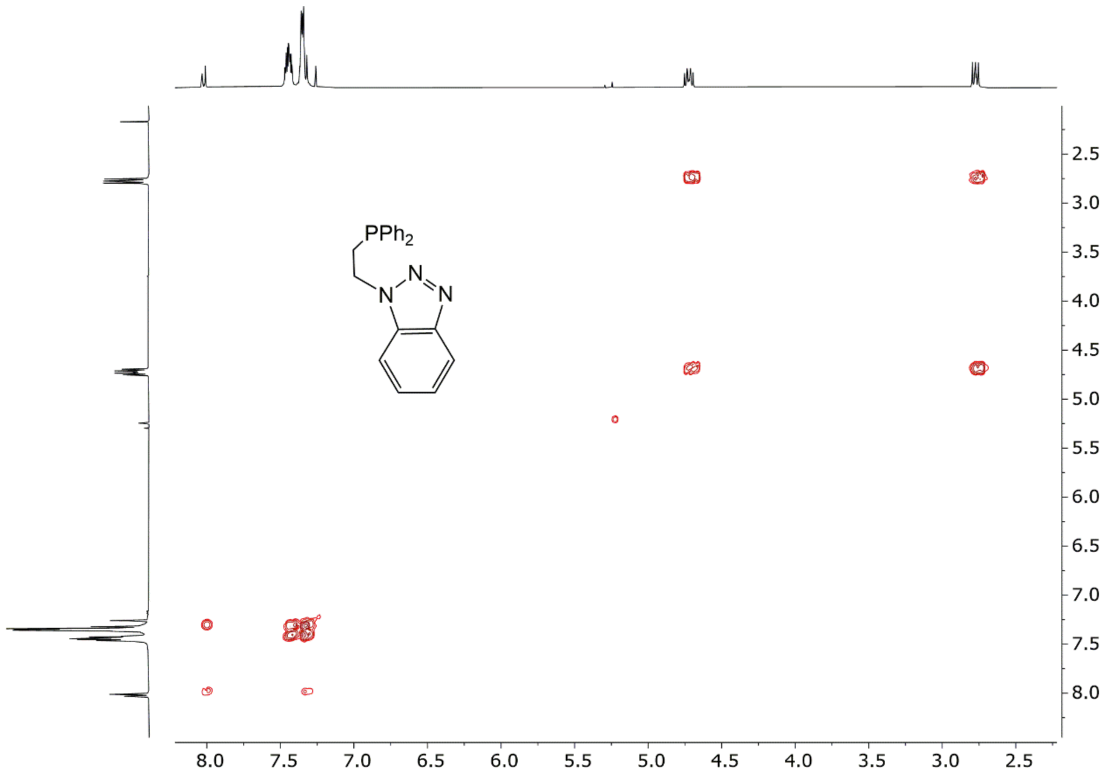

Figure S5. ^{1}H-^{1}H COSY spectrum of **1** in $CDCl_3$.

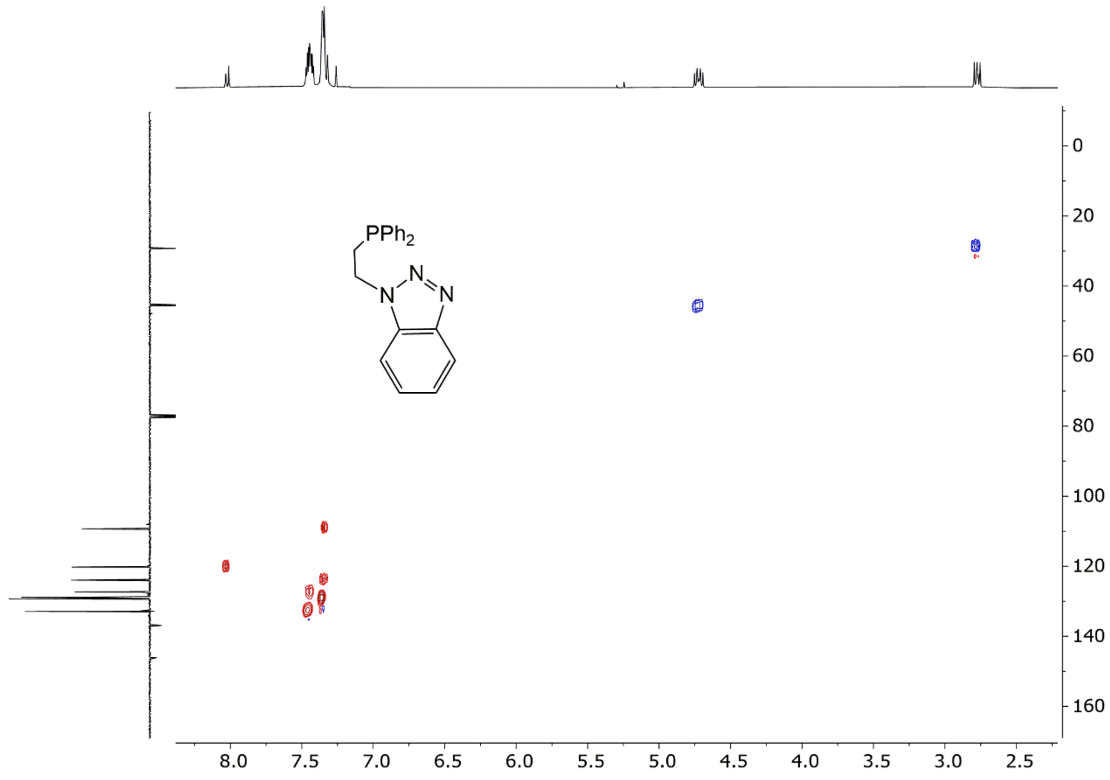

Figure S6. ^{1}H-^{13}C HSQC spectrum of **1** in $CDCl_3$.

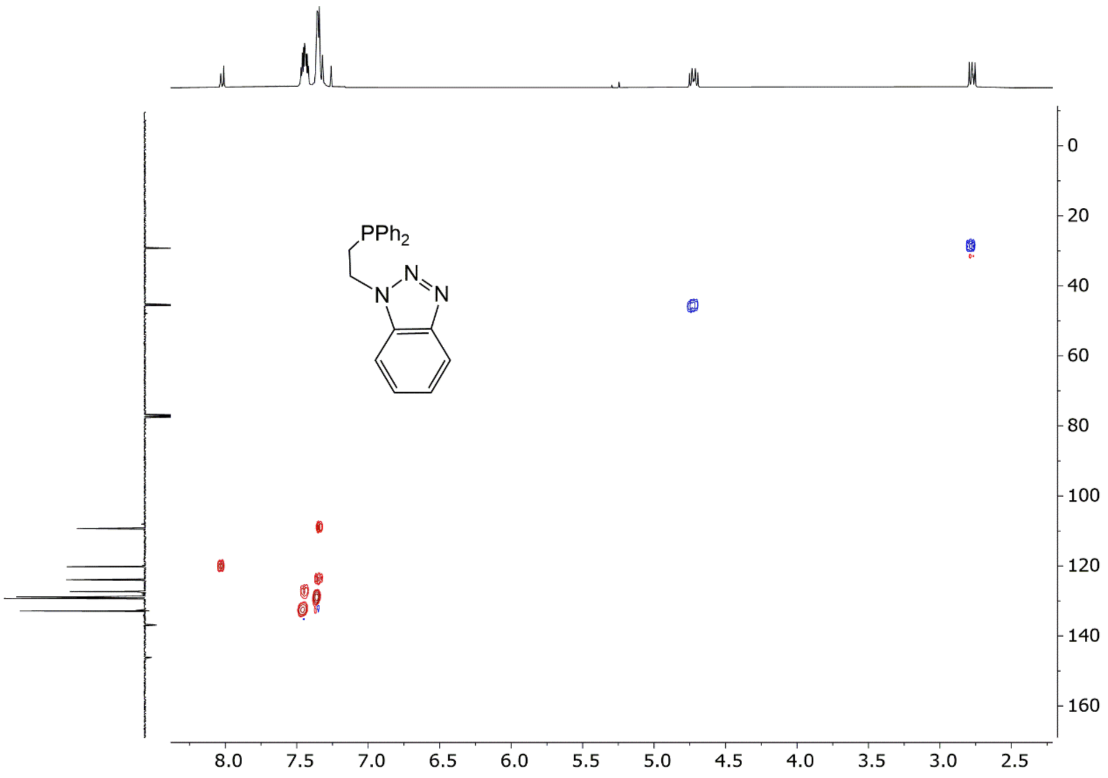

Figure S7. ^{1}H-^{13}C HMBC spectrum of **1** in $CDCl_3$.

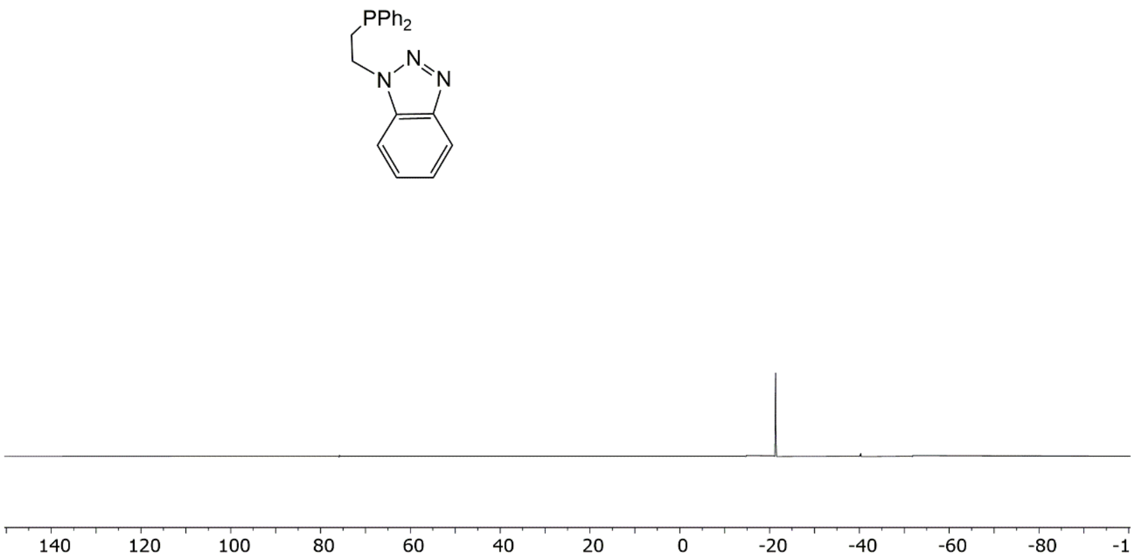

Figure S8. ^{31}P{^{1}H} NMR spectrum of **1** in $CDCl_3$.

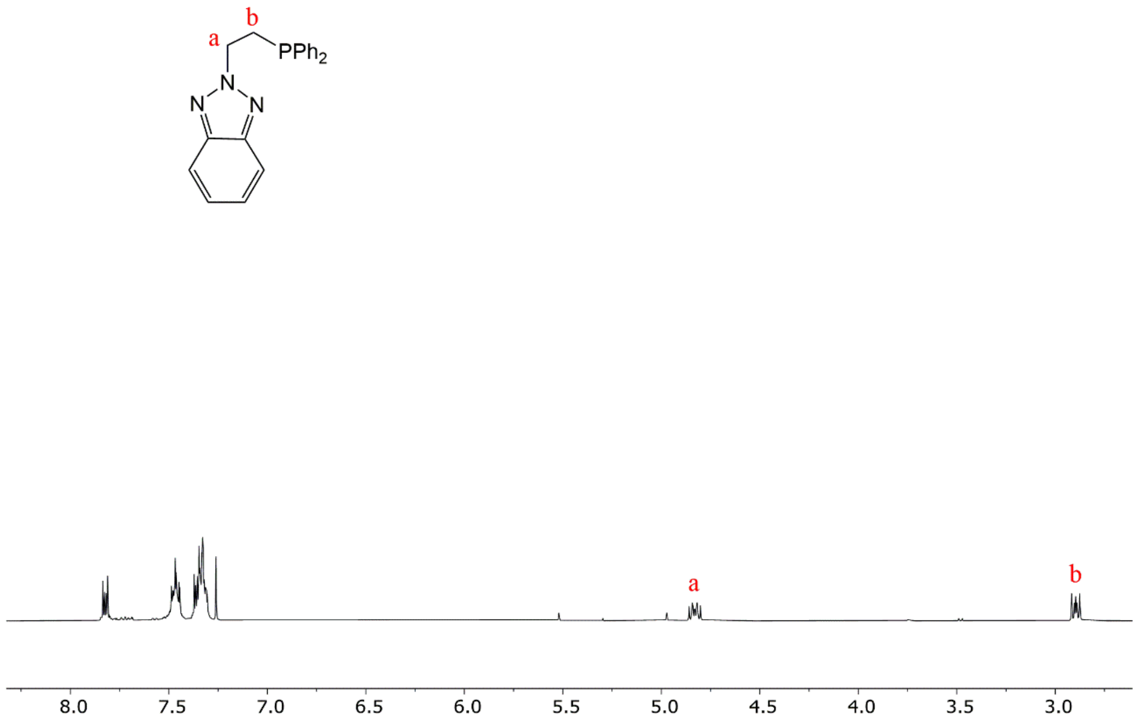

Figure S9. ^{1}H NMR spectrum of **2** in $CDCl_3$.

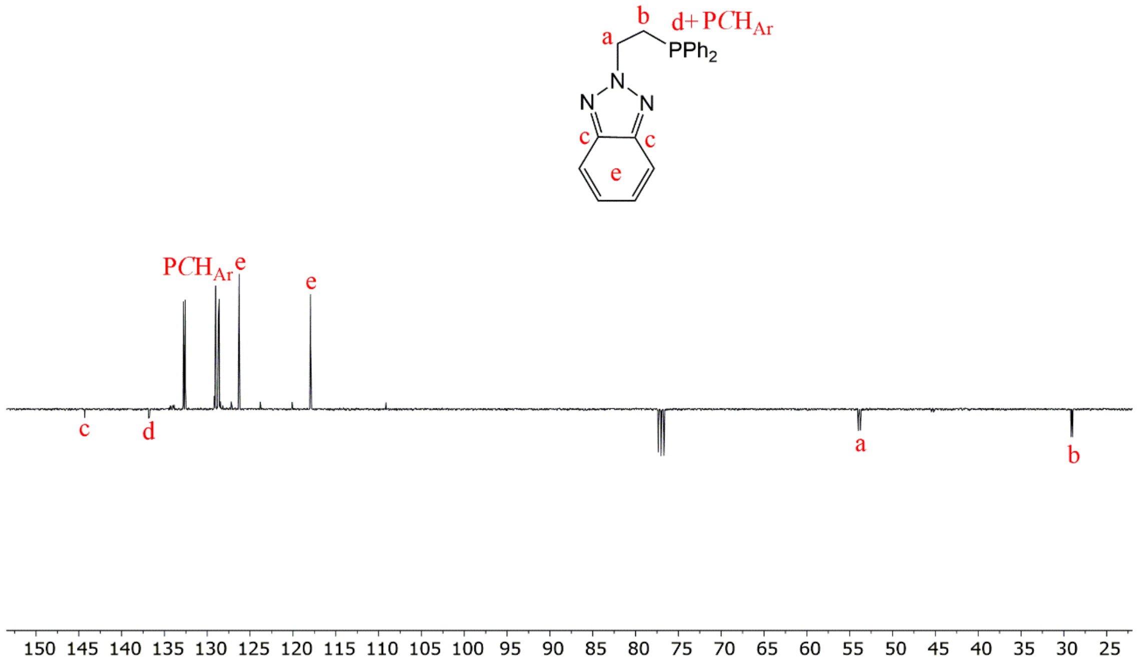

Figure S10. ^{13}C{^{1}H} NMR APT spectrum of **2** in $CDCl_3$.

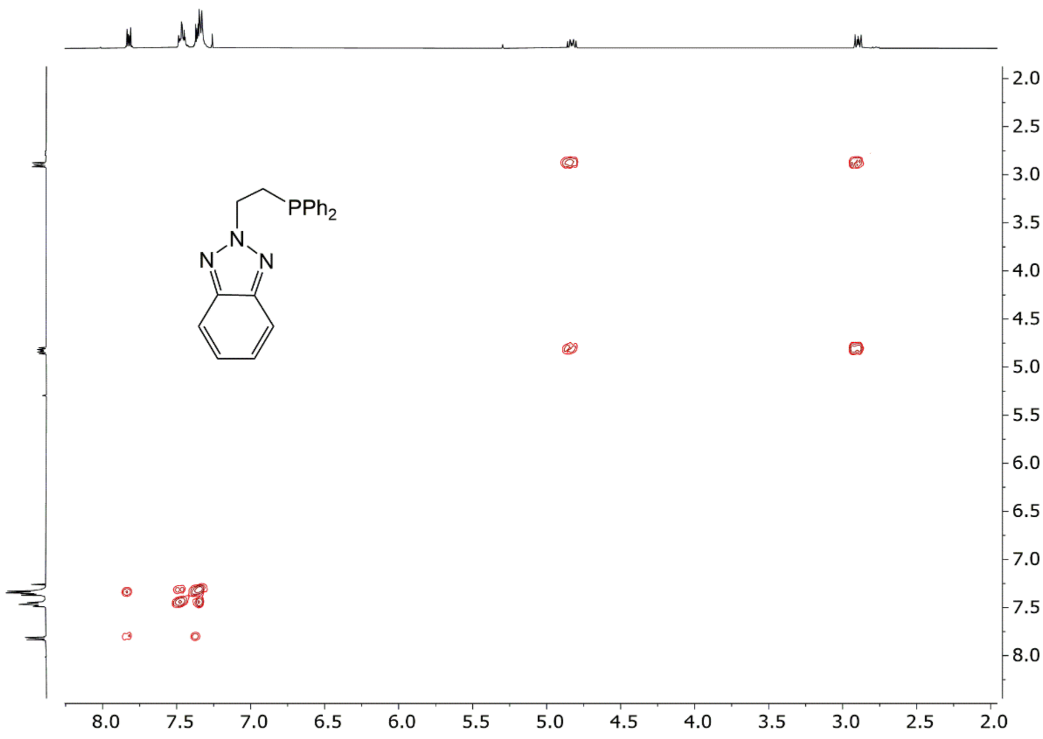

Figure S11. ^{1}H-^{1}H COSY spectrum of **2** in $CDCl_3$.

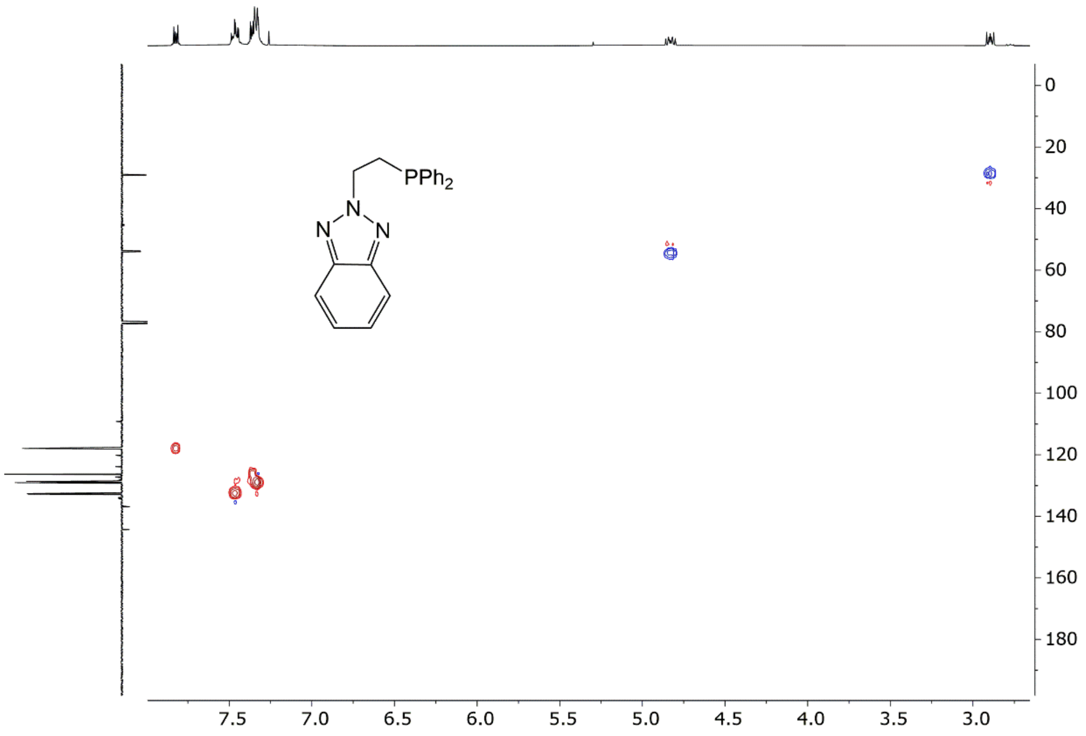

Figure S12. ^{1}H-^{13}C HSQC spectrum of **2** in $CDCl_3$.

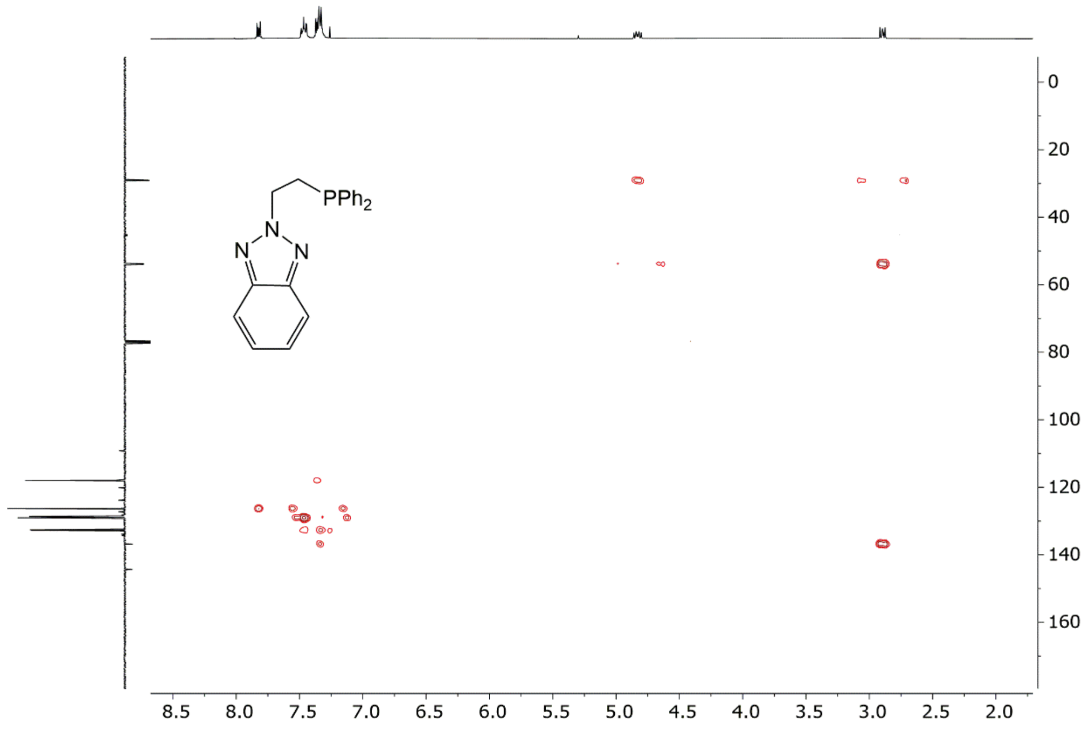

Figure S13. ^{1}H-^{13}C HMBC spectrum of **2** in CDCl3.

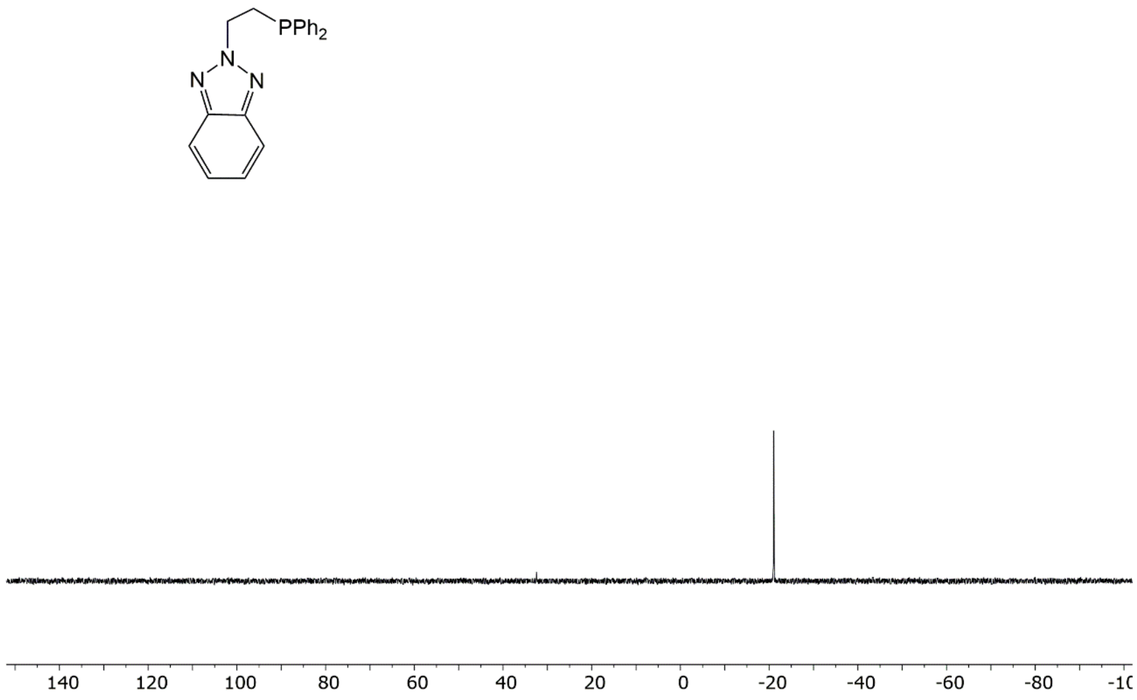

Figure S14. ^{31}P{^{1}H} NMR spectrum of **2** in CDCl3.

NMR spectra of organometallic complexes of Ir(I)

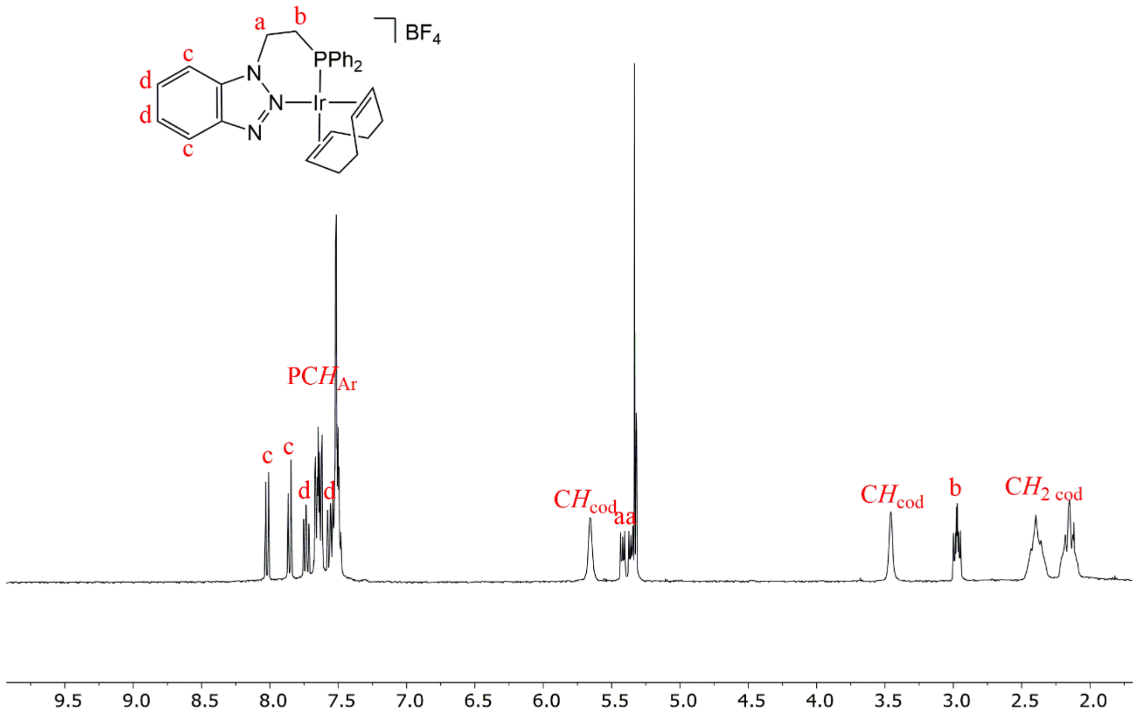

Figure S15. ^{1}H NMR spectrum of **Ir-1** in CD_2Cl_2.

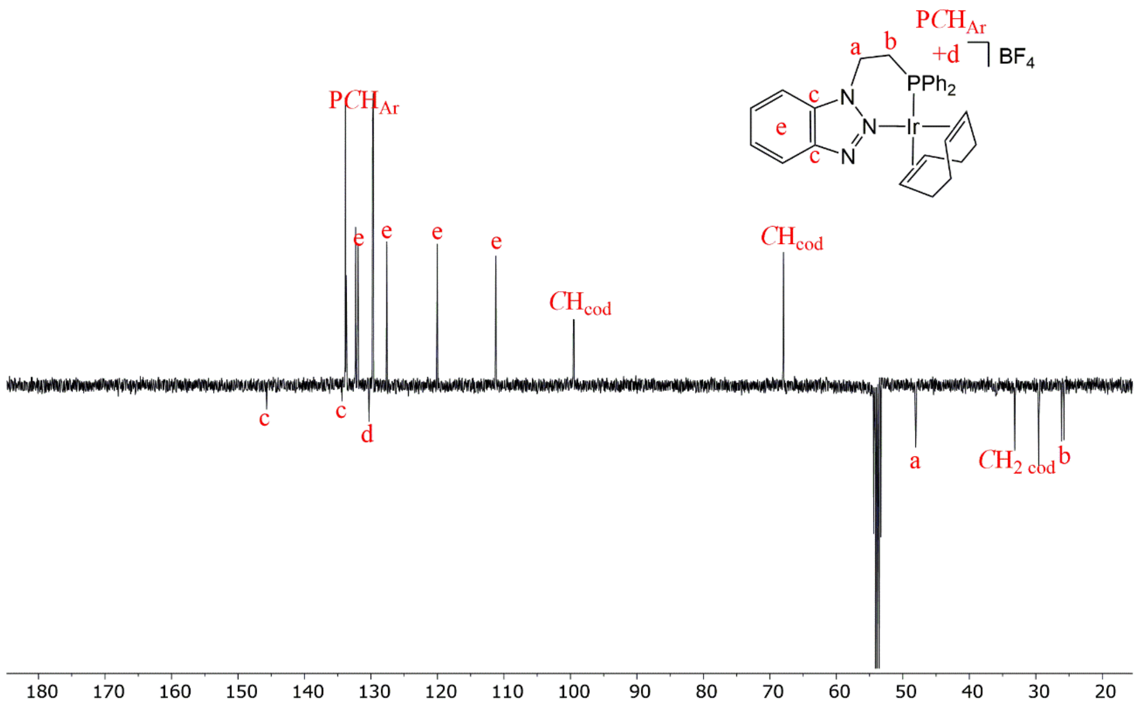

Figure S16. ^{13}C{^{1}H} NMR APT spectrum of **Ir-1** in CD_2Cl_2.

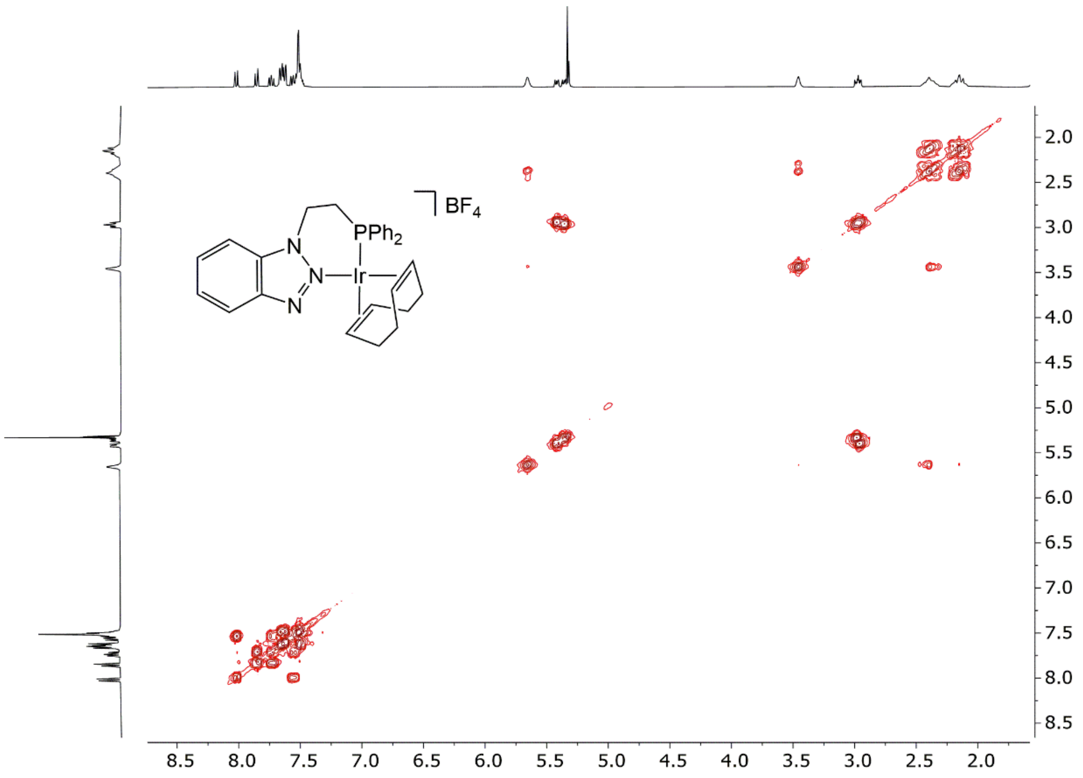

Figure S17. ^{1}H-^{1}H COSY spectrum of **Ir-1** in CD_2Cl_2.

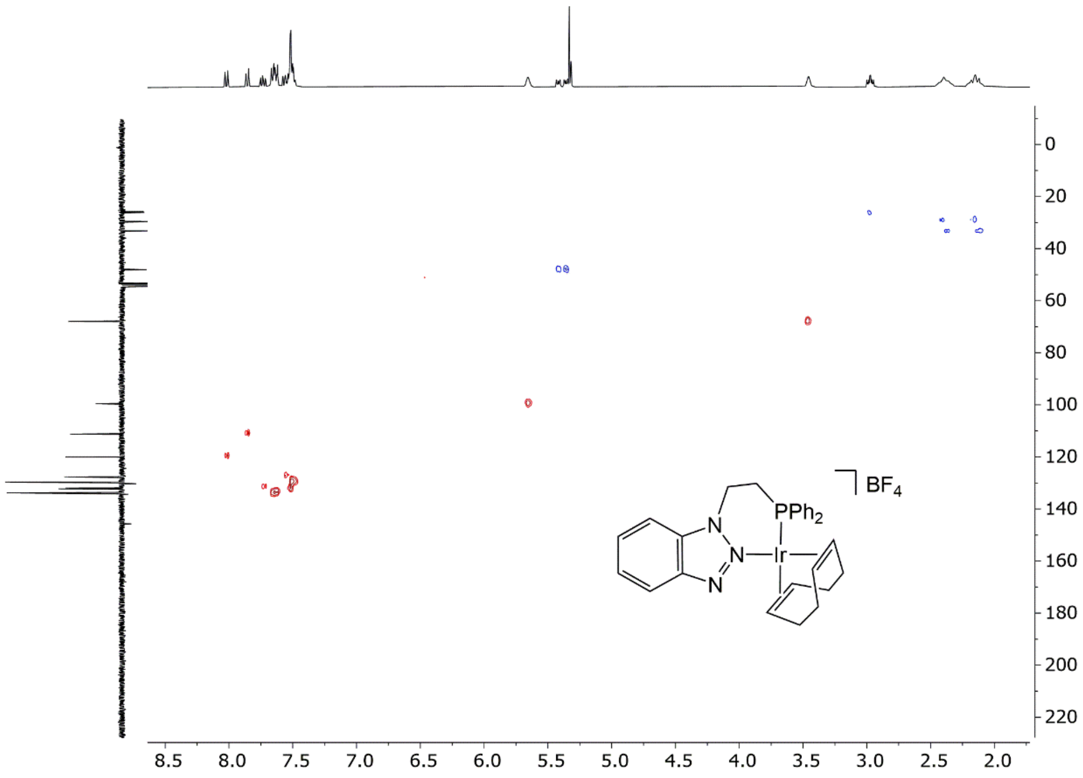

Figure S18. ^{1}H-^{13}C HSQC spectrum of **Ir-1** in CD_2Cl_2.

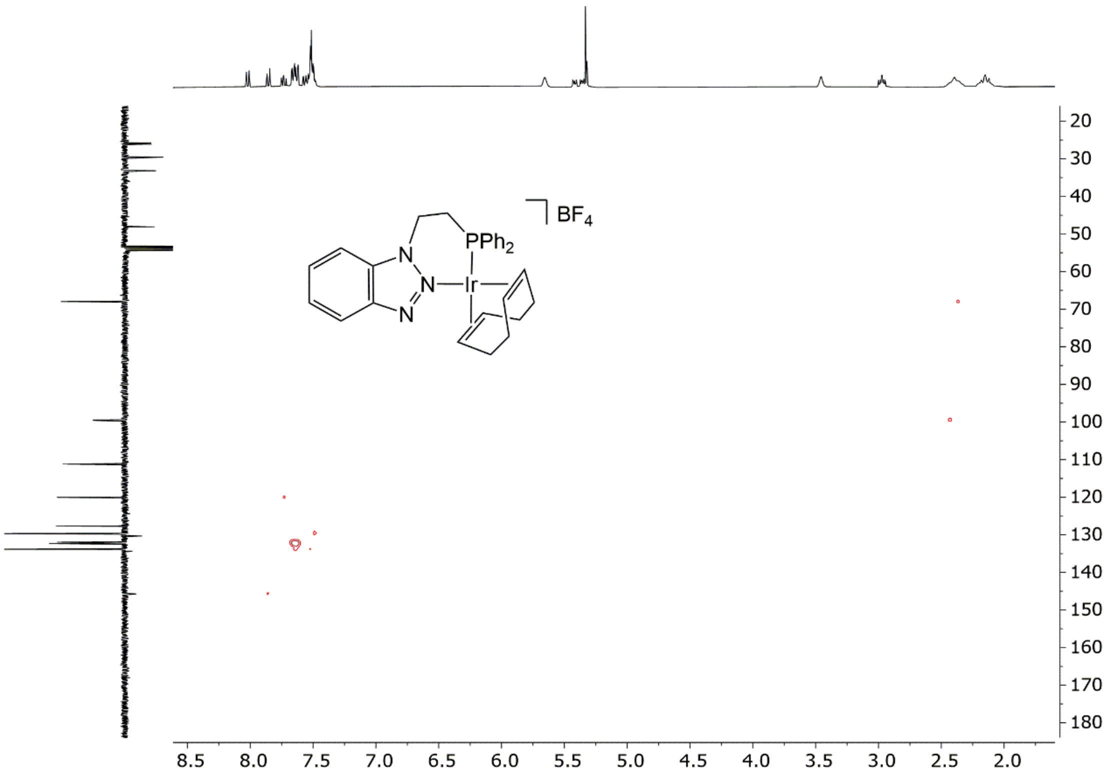

Figure S19. ^{1}H-^{13}C HMBC spectrum of **Ir-1** in CD_2Cl_2.

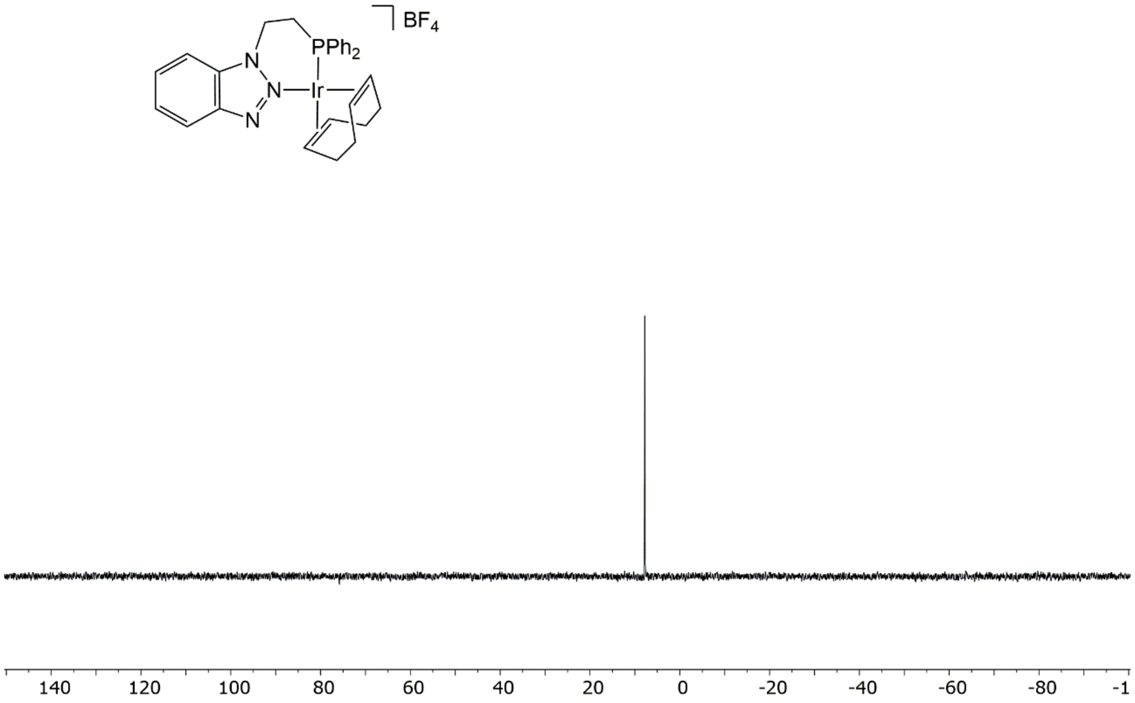

Figure S20. ^{31}P{^{1}H} NMR spectrum of **Ir-1** in CD_2Cl_2.

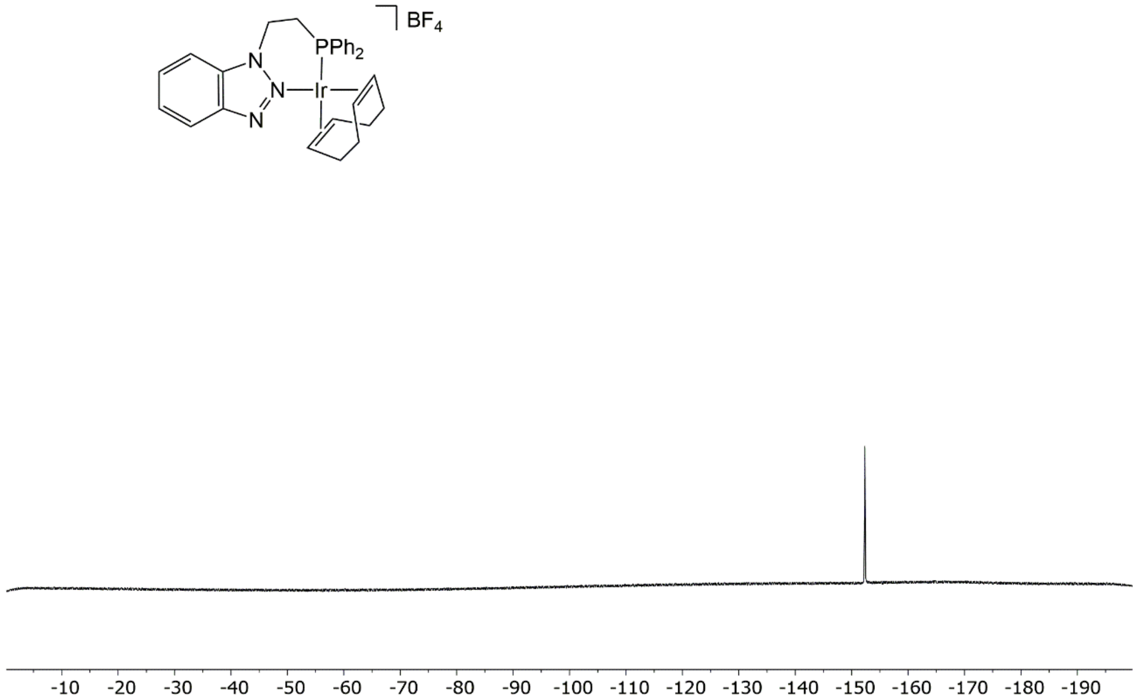

Figure S21. ^{19}F NMR spectrum of **Ir-1** in CD_2Cl_2.

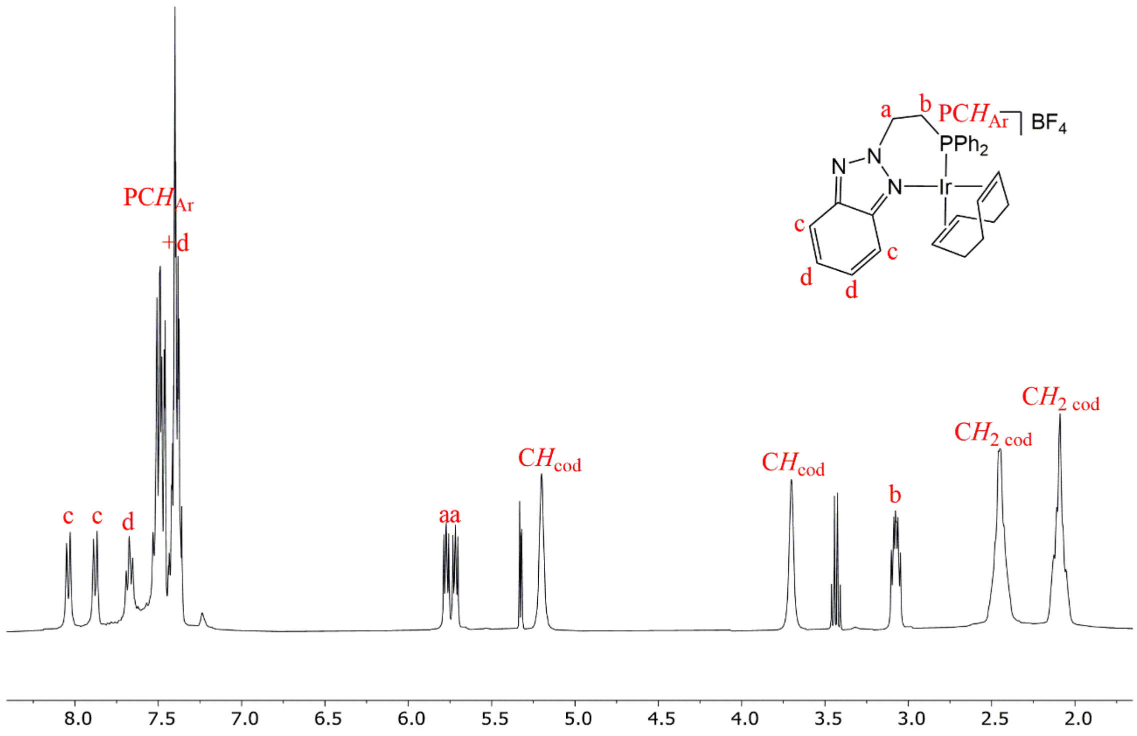

Figure S22. ^{1}H NMR spectrum of **Ir-2** in CD_2Cl_2.

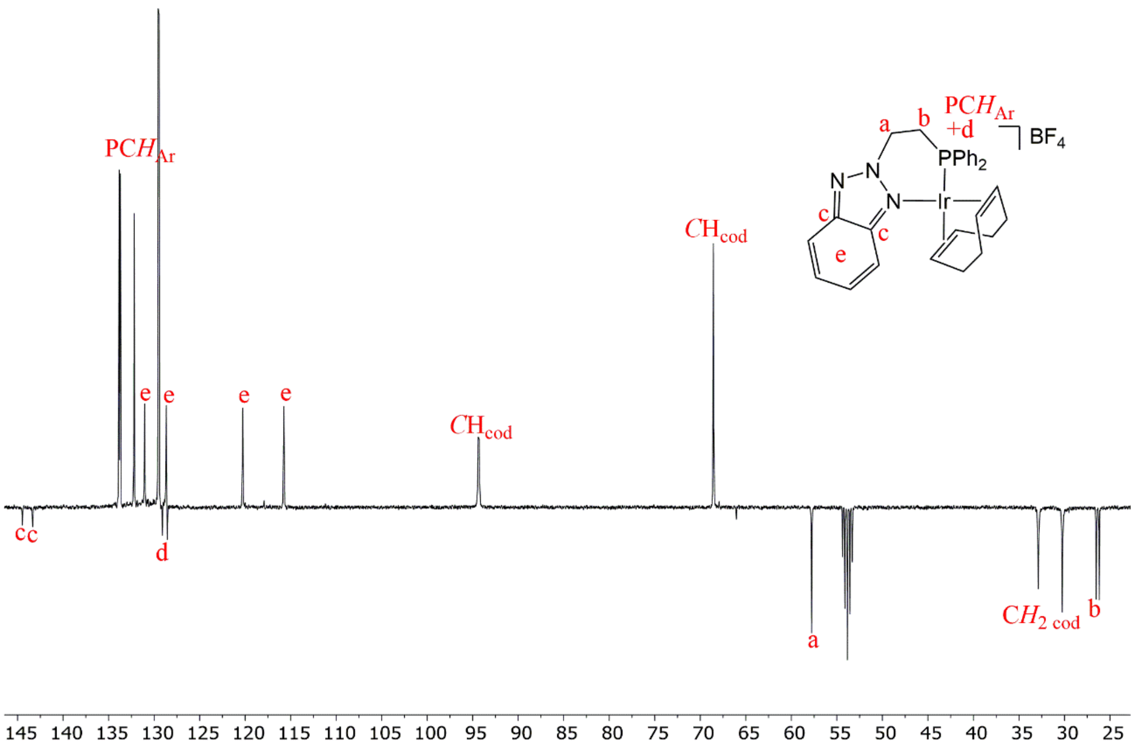

Figure S23. $^{13}C\{^{1}H\}$ NMR APT spectrum of **Ir-2** in CD_2Cl_2.

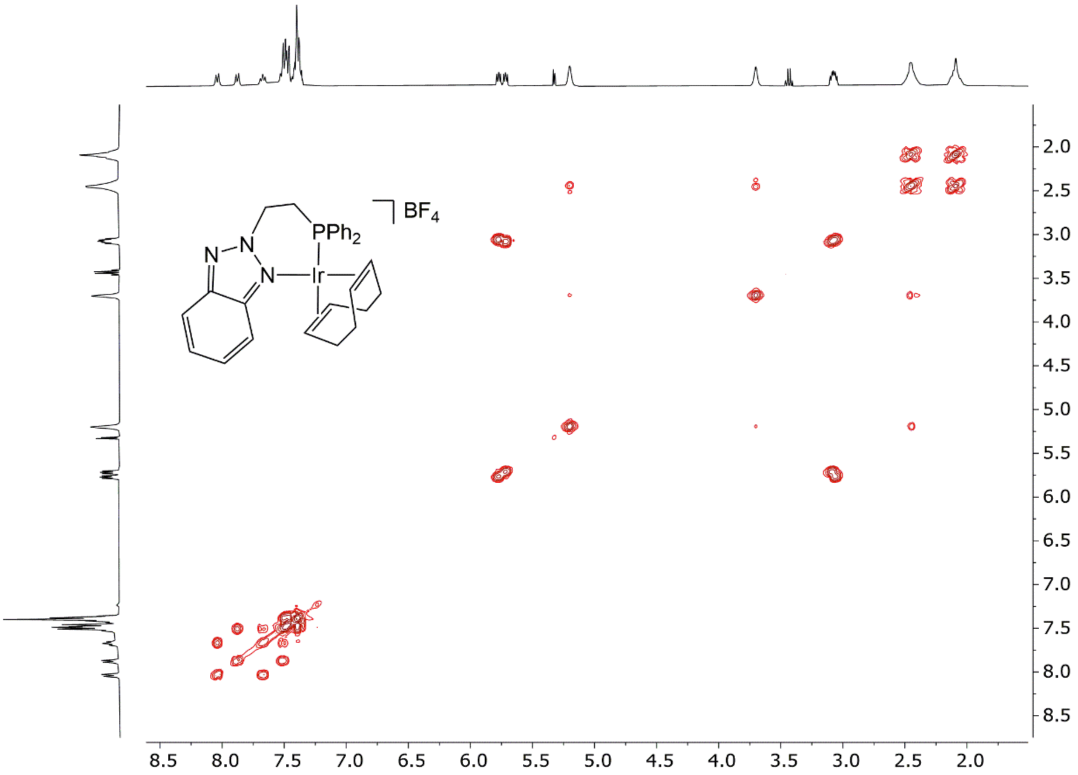

Figure S24. ^{1}H-^{1}H COSY spectrum of **Ir-2** in CD_2Cl_2.

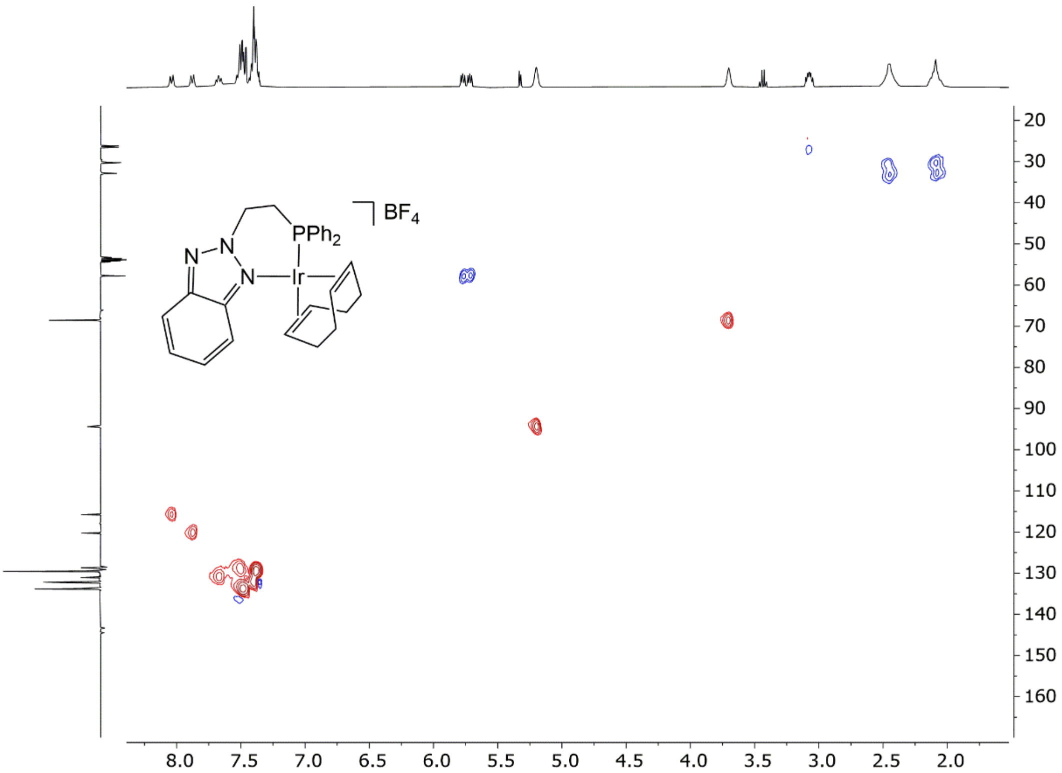

Figure S25. ^{1}H-^{13}C HSQC spectrum of **Ir-2** in CD_2Cl_2.

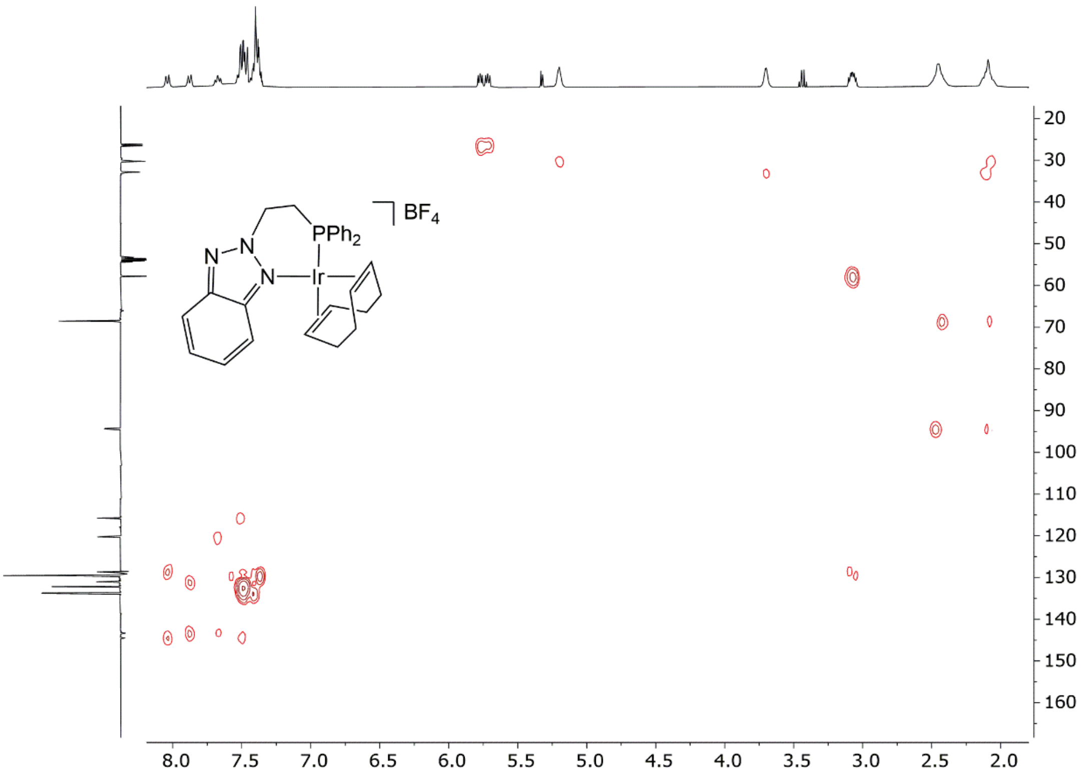

Figure S26. ^{1}H-^{13}C HMBC spectrum of **Ir-2** in CD_2Cl_2.

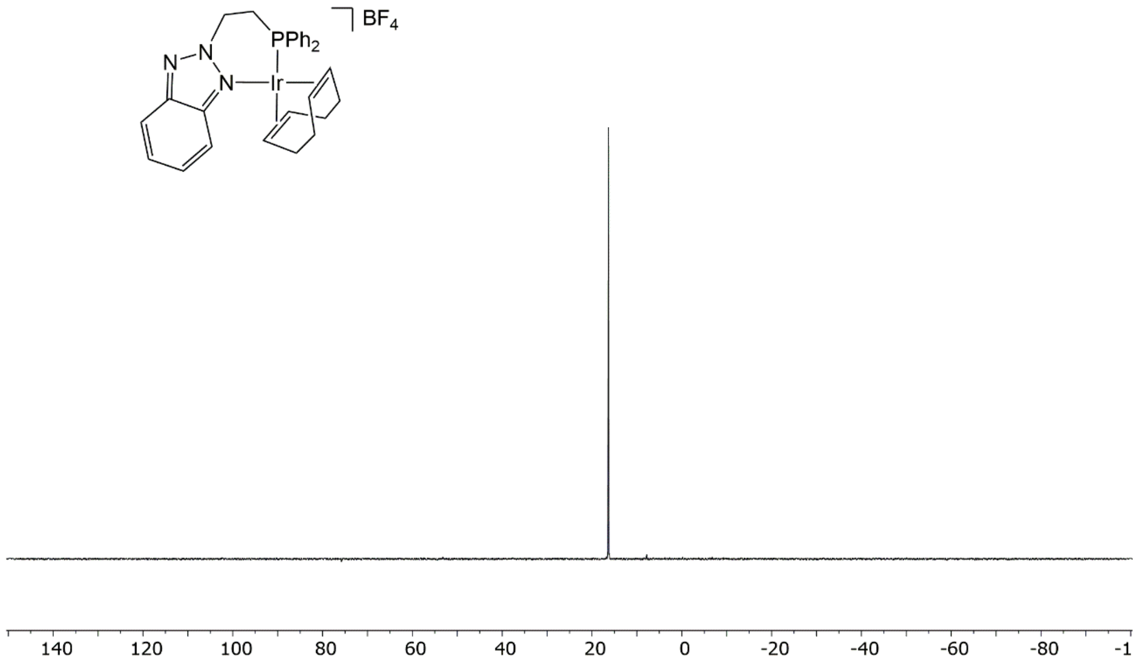

Figure S27. $^{31}P\{^{1}H\}$ NMR spectrum of **Ir-2** in CD_2Cl_2.

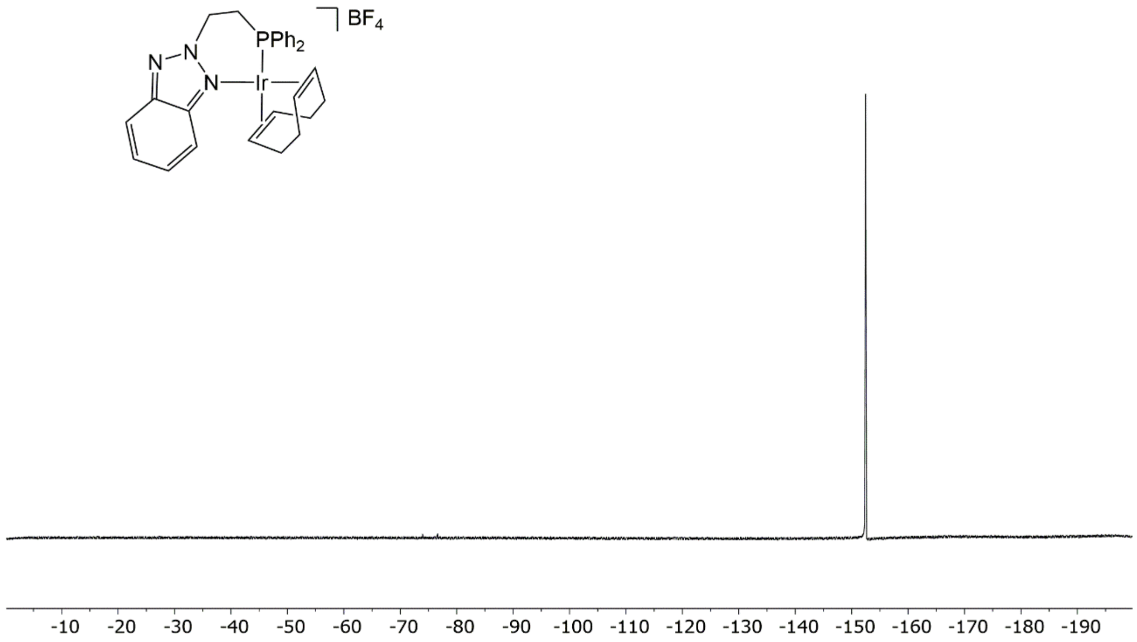

Figure S28. ^{19}F NMR spectrum of **Ir-2** in CD_2Cl_2.

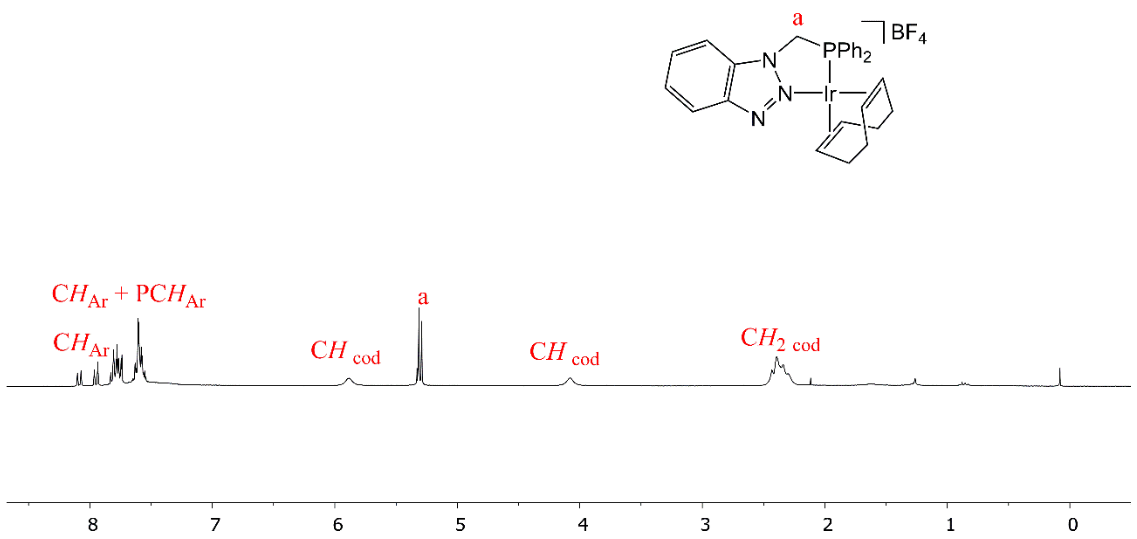

Figure S29. ^{1}H NMR spectrum of **Ir-3** in CD_2Cl_2.

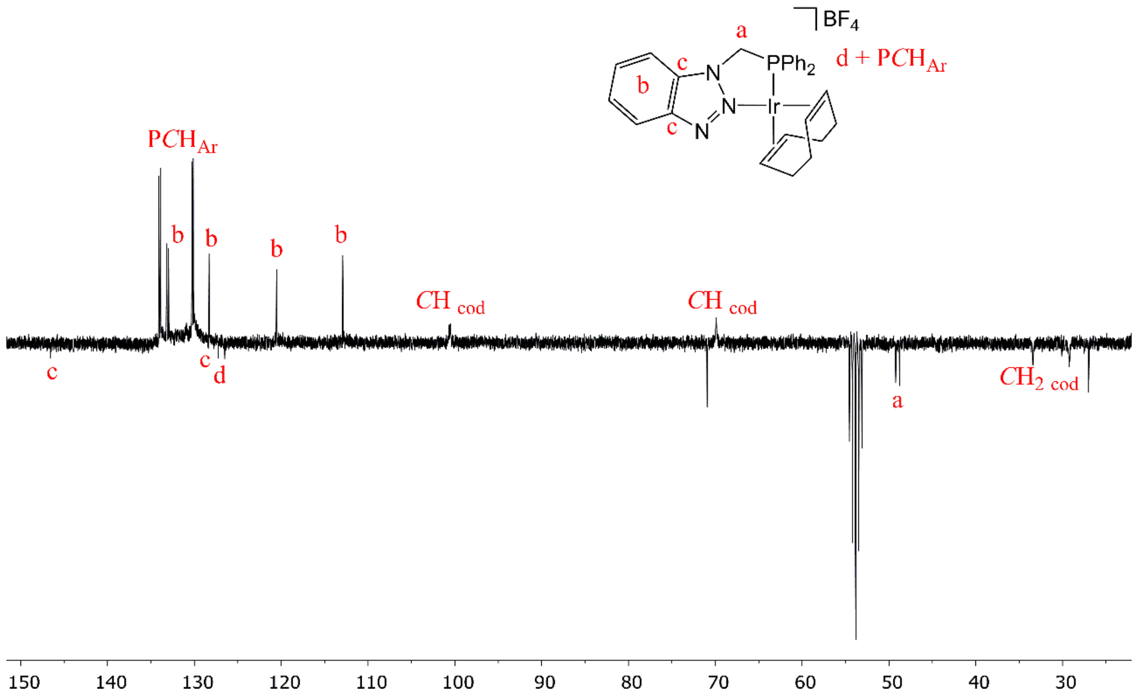

Figure S30. $^{13}C\{^{1}H\}$ NMR APT spectrum of **Ir-3** in CD_2Cl_2.

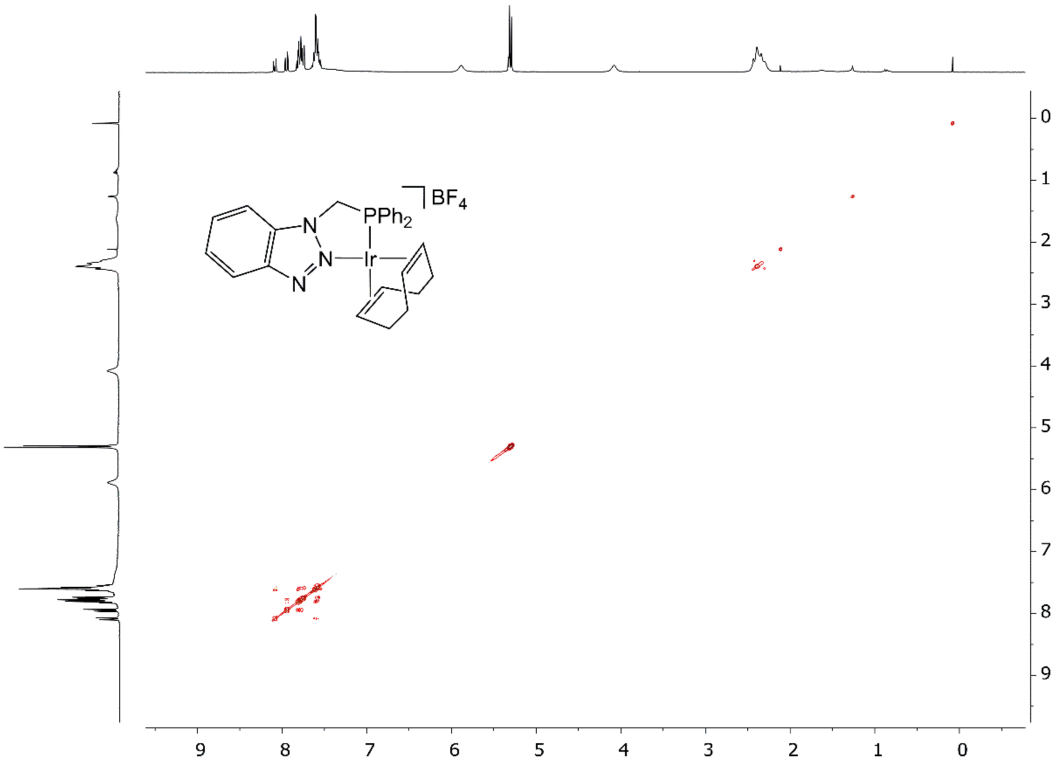

Figure S31. ^{1}H-^{1}H COSY spectrum of **Ir-3** in CD_2Cl_2.

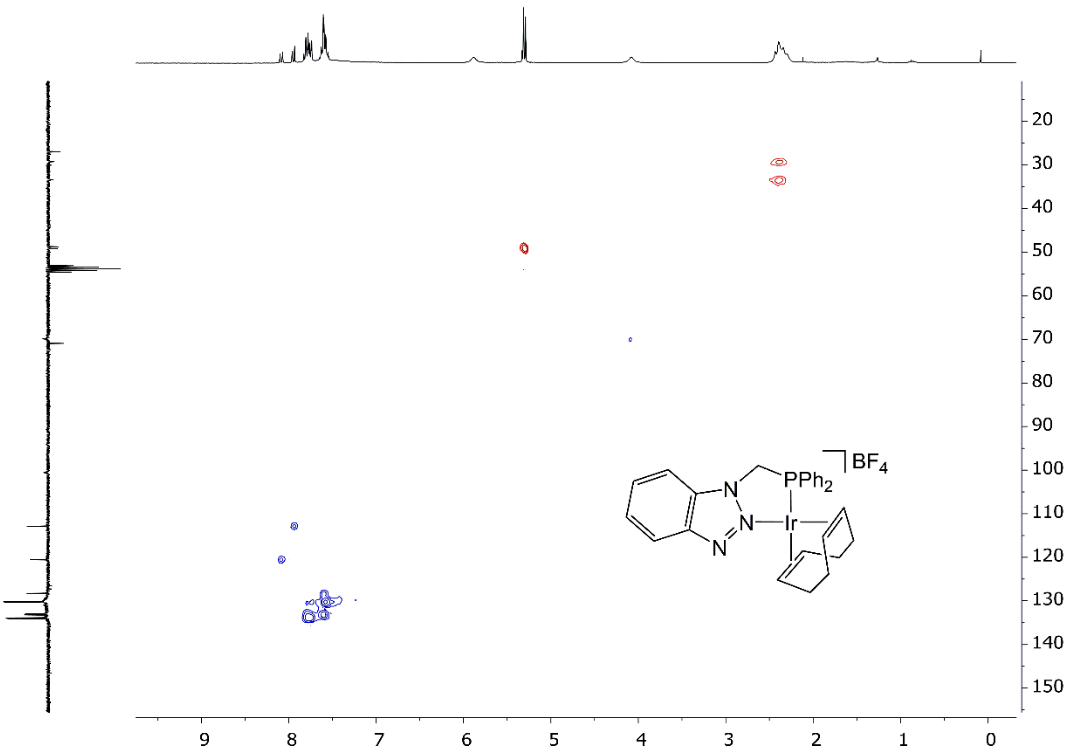

Figure S32. ^{1}H-^{13}C HSQC spectrum of **Ir-3** in CD_2Cl_2.

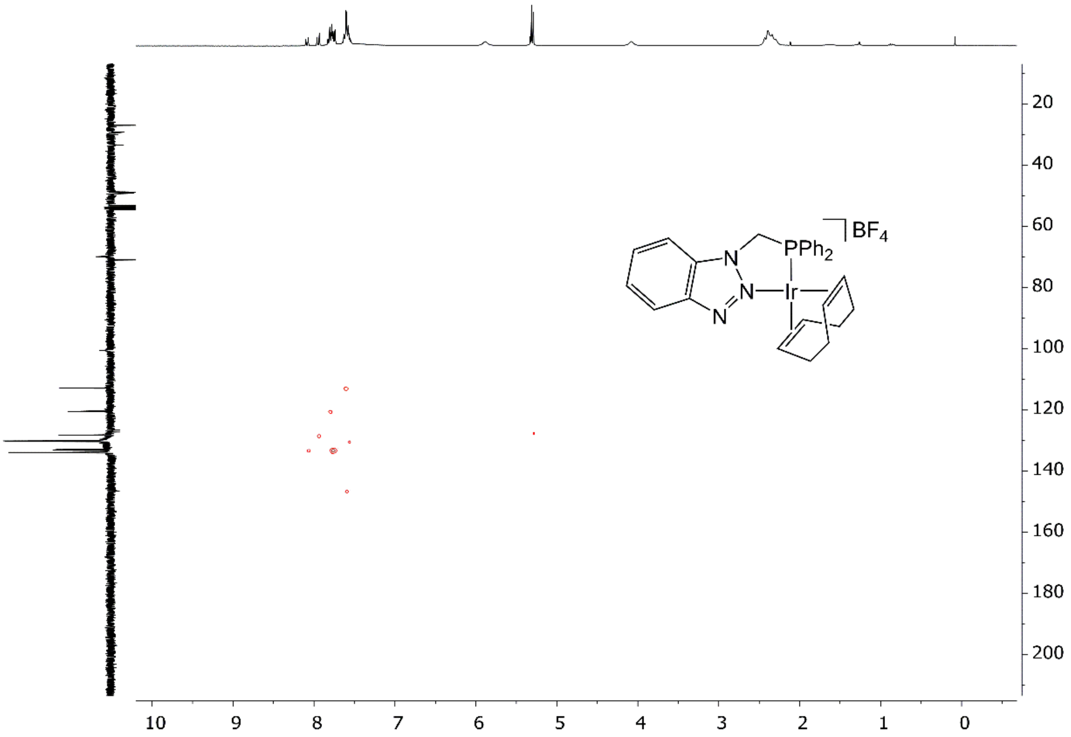

Figure S33. ^{1}H-^{13}C HMBC spectrum of **Ir-3** in CD_2Cl_2.

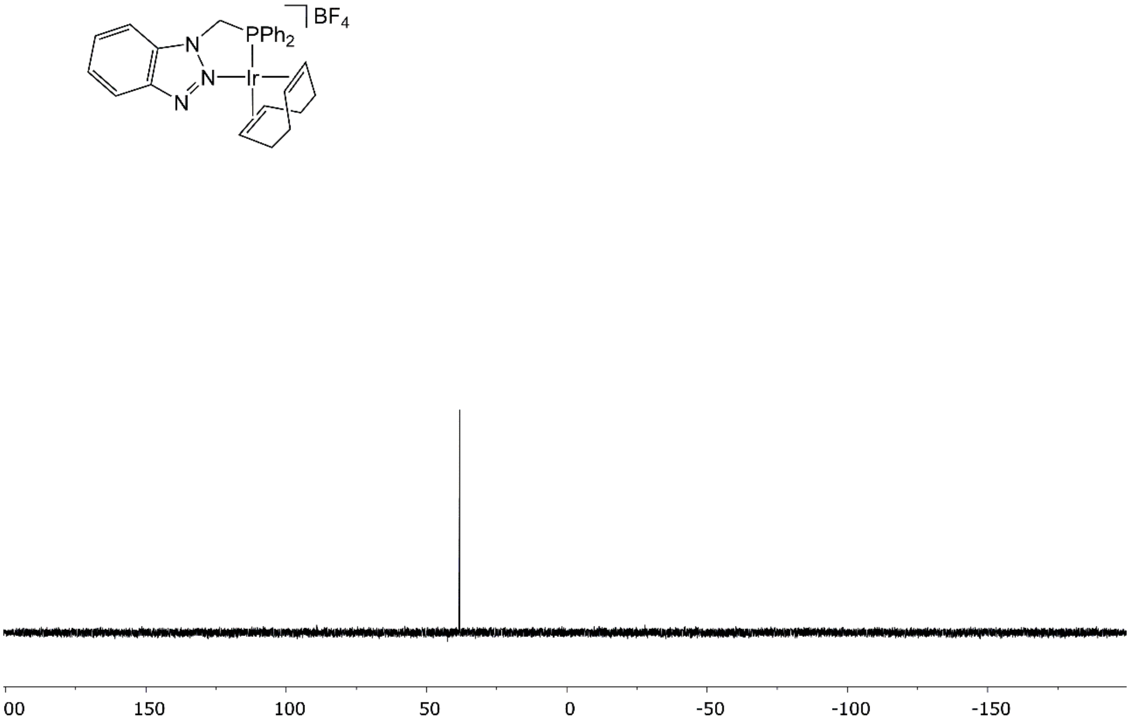

Figure S34. $^{31}P\{^{1}H\}$ NMR spectrum of **Ir-3** in CD_2Cl_2.

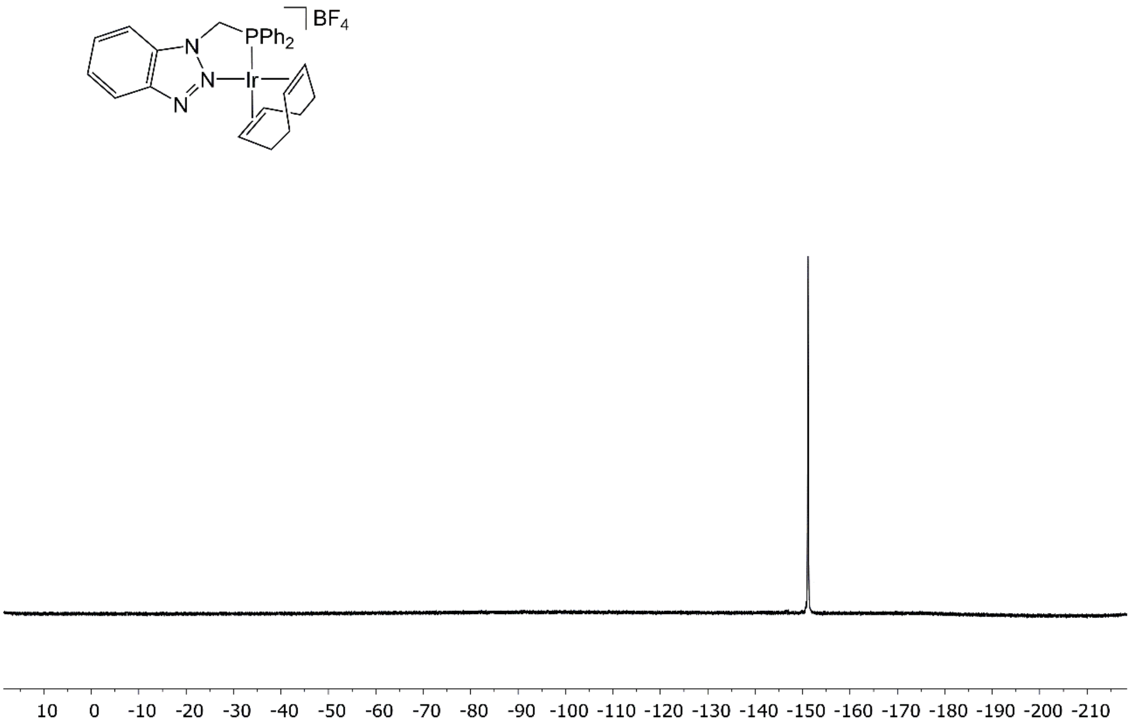

Figure S35. ^{19}F NMR spectrum of **Ir-3** in CD_2Cl_2.

NMR spectra of organometallic complexes of Rh(I)

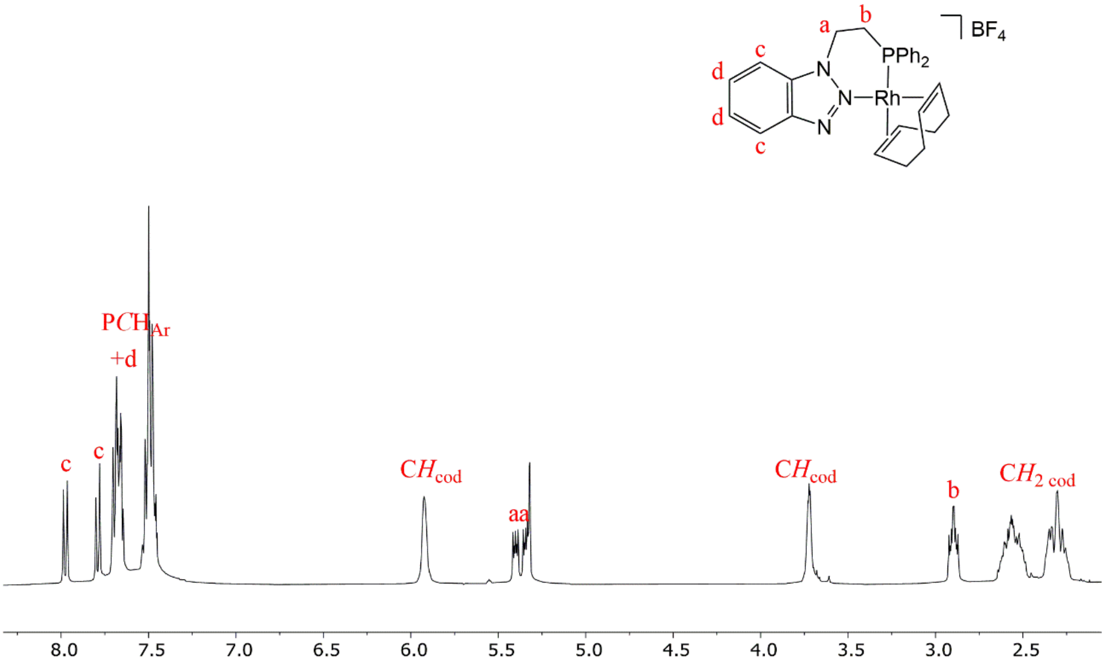

Figure S36. ^{1}H NMR spectrum of **Rh-1** in CD_2Cl_2.

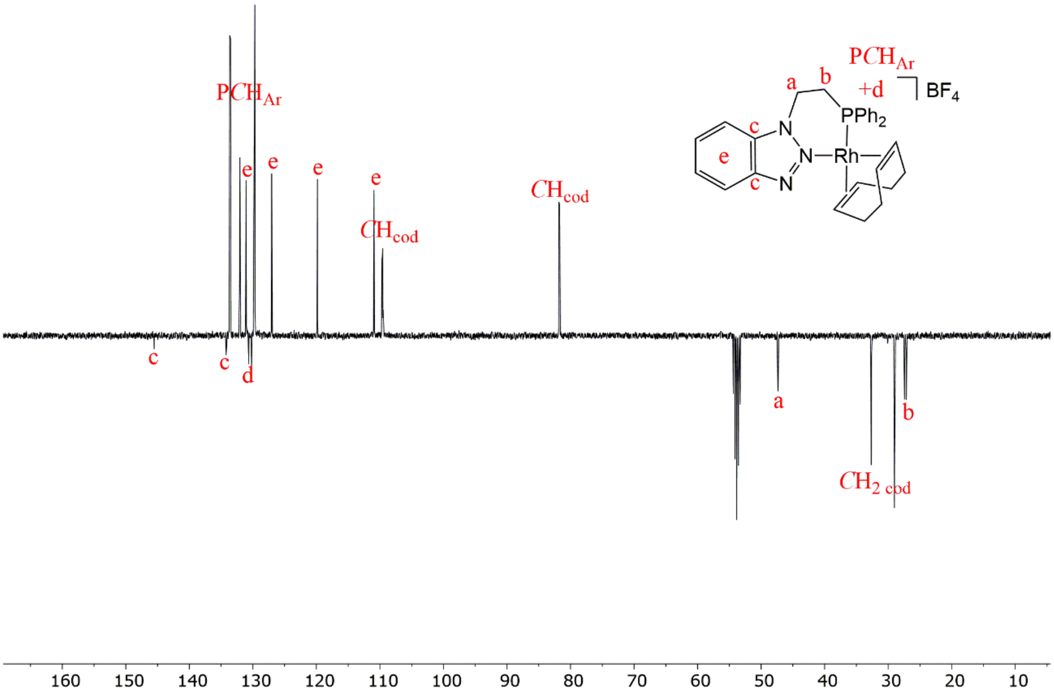

Figure S37. ^{13}C{^{1}H} NMR APT spectrum of **Rh-1** in CD_2Cl_2.

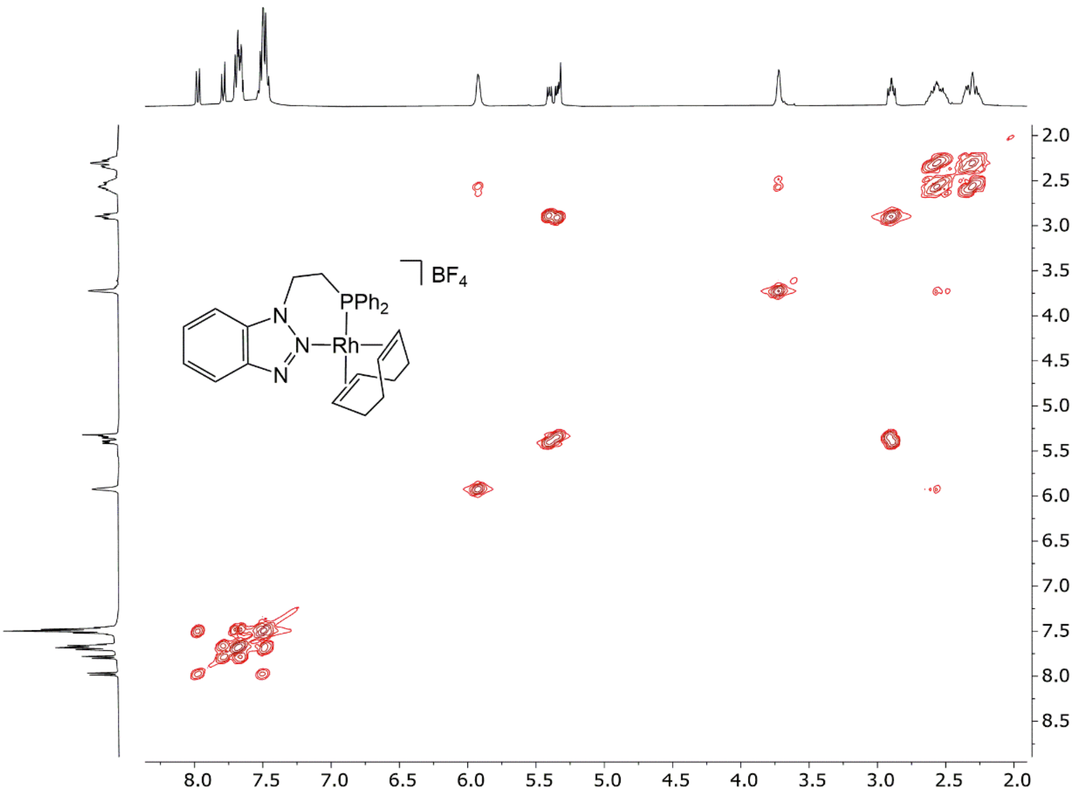

Figure S38. ^{1}H-^{1}H COSY spectrum of **Rh-1** in CD_2Cl_2.

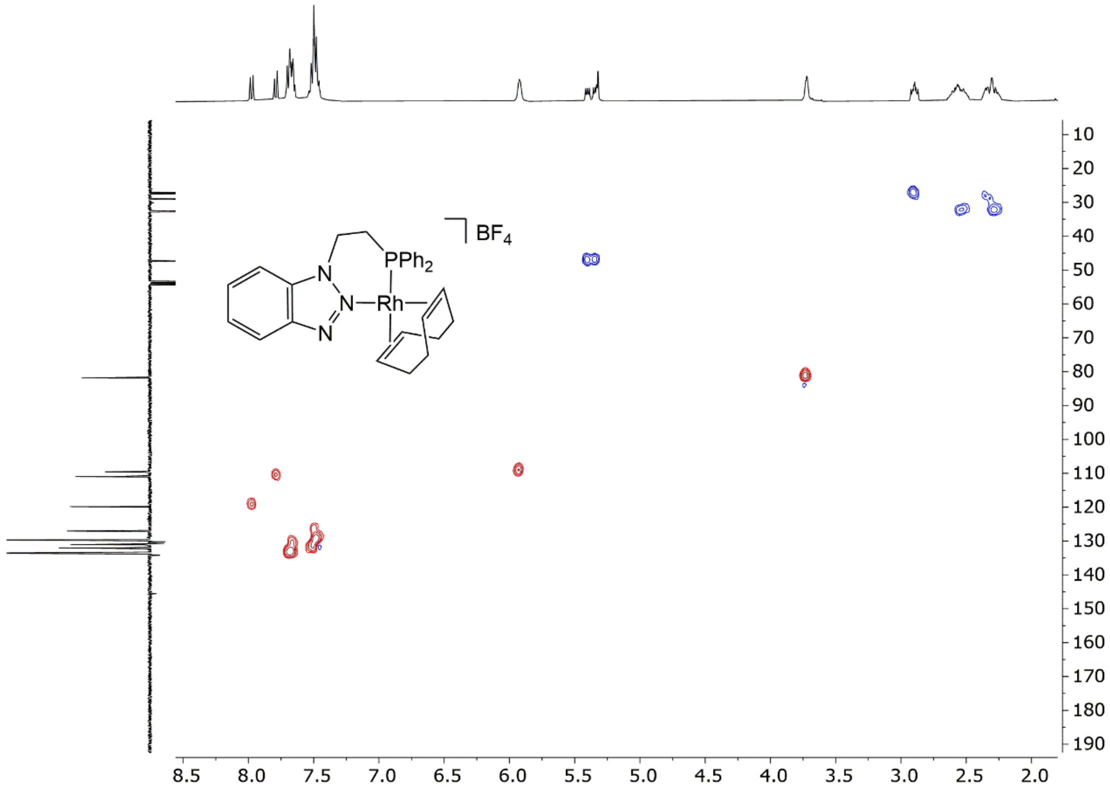

Figure S39. ^{1}H-^{13}C HSQC spectrum of **Rh-1** in CD_2Cl_2.

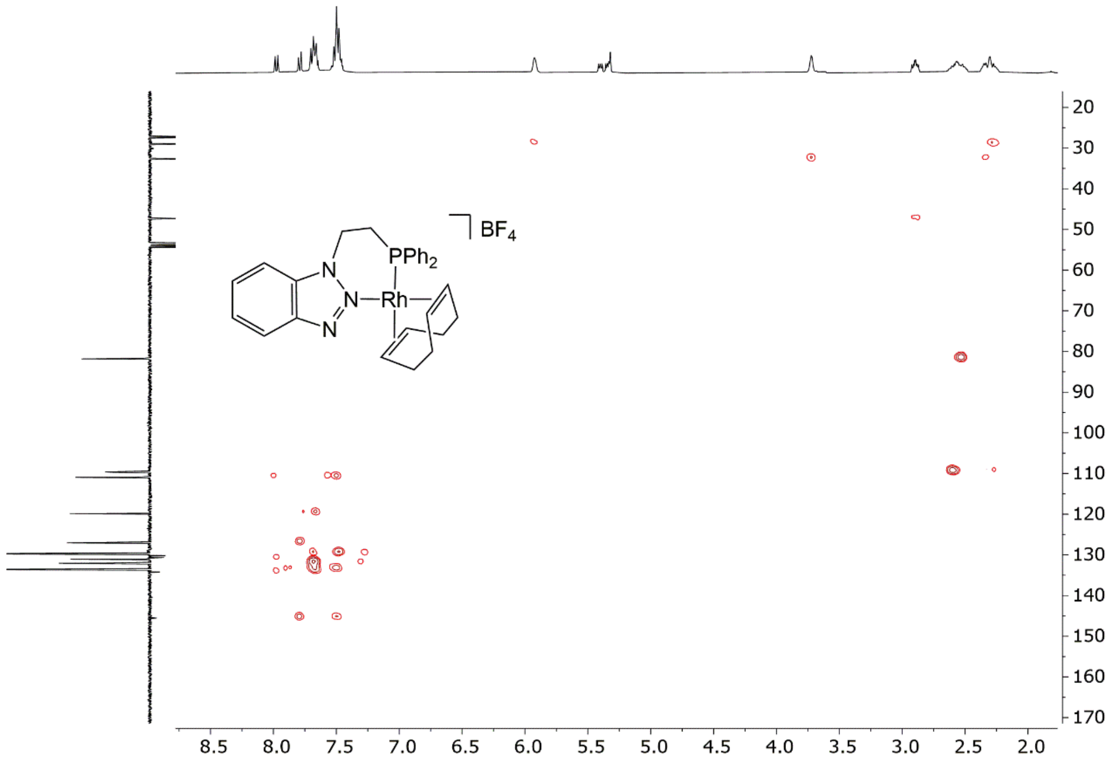

Figure S40. ^{1}H-^{13}C HMBC spectrum of **Rh-1** in CD_2Cl_2.

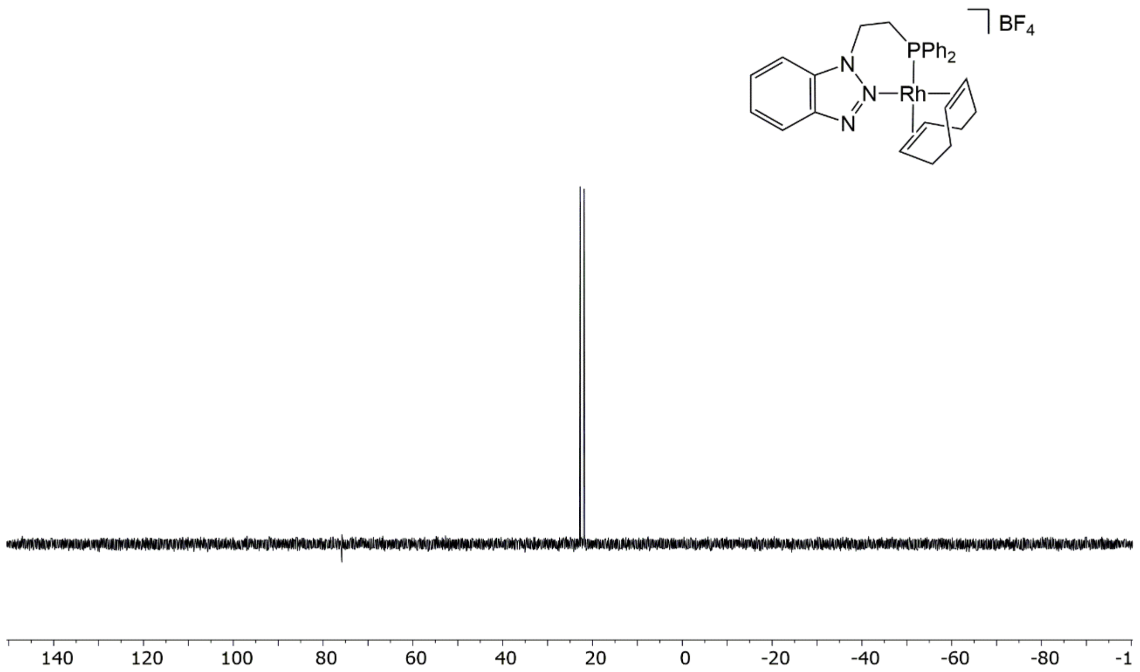

Figure S41. $^{31}P\{^{1}H\}$ NMR spectrum of **Rh-1** in CD_2Cl_2.

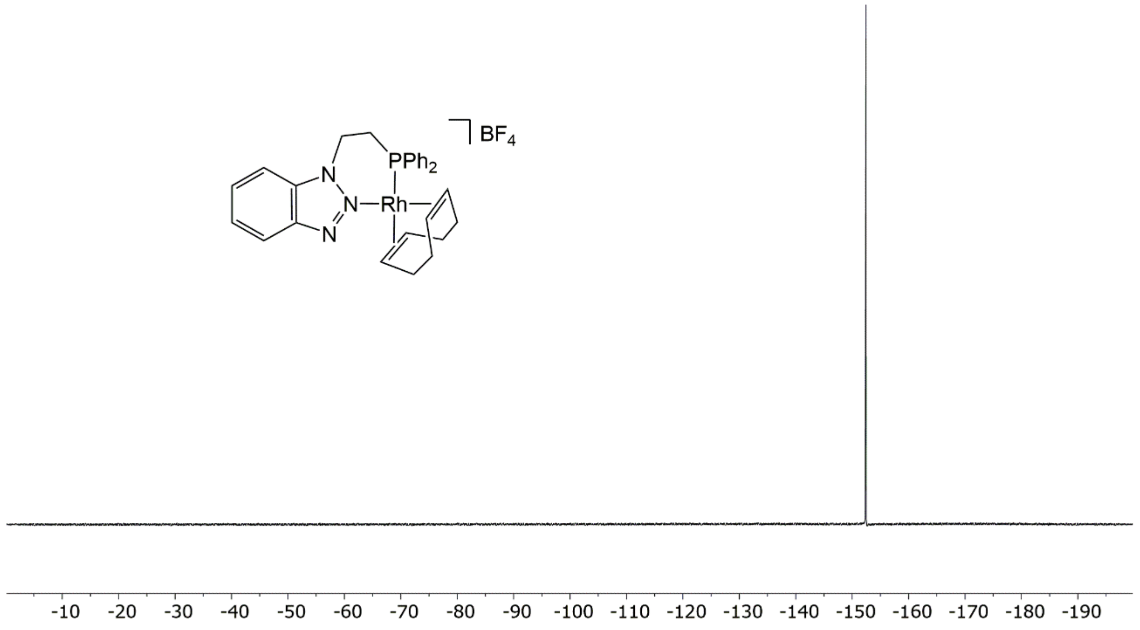

Figure S42. ^{19}F NMR spectrum of **Rh-1** in CD_2Cl_2.

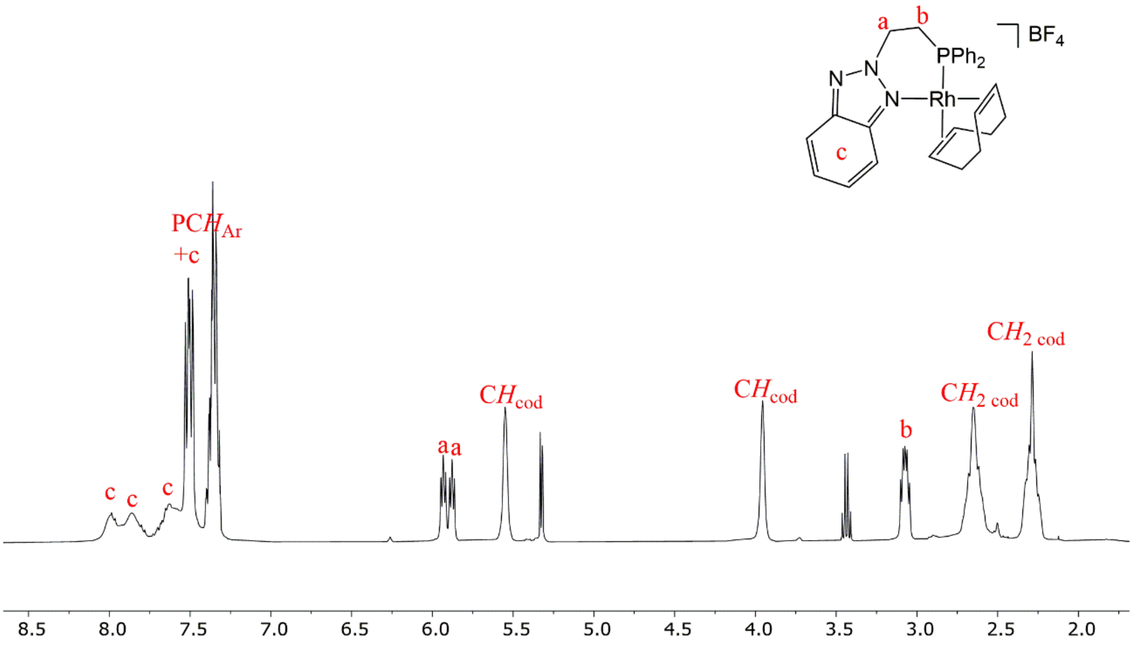

Figure S43. ^{1}H NMR spectrum of **Rh-2** in CD_2Cl_2.

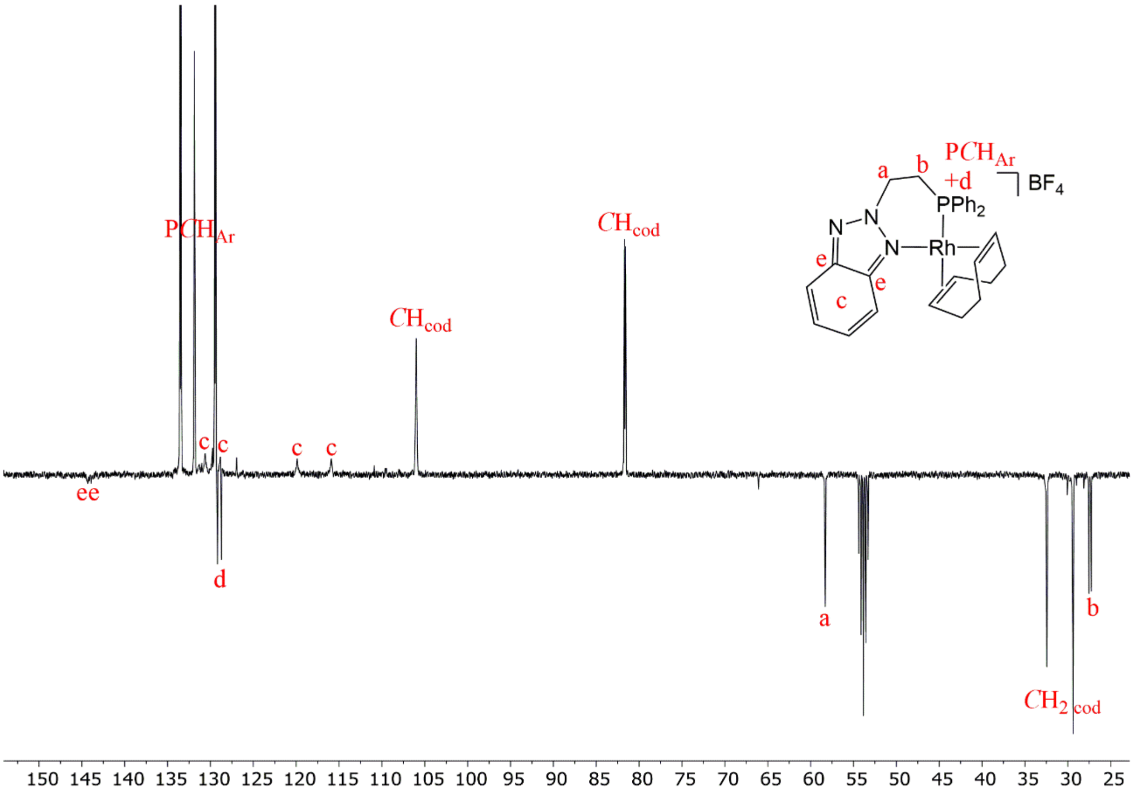

Figure S44. $^{13}C\{^1H\}$ NMR APT spectrum of **Rh-2** in CD_2Cl_2.

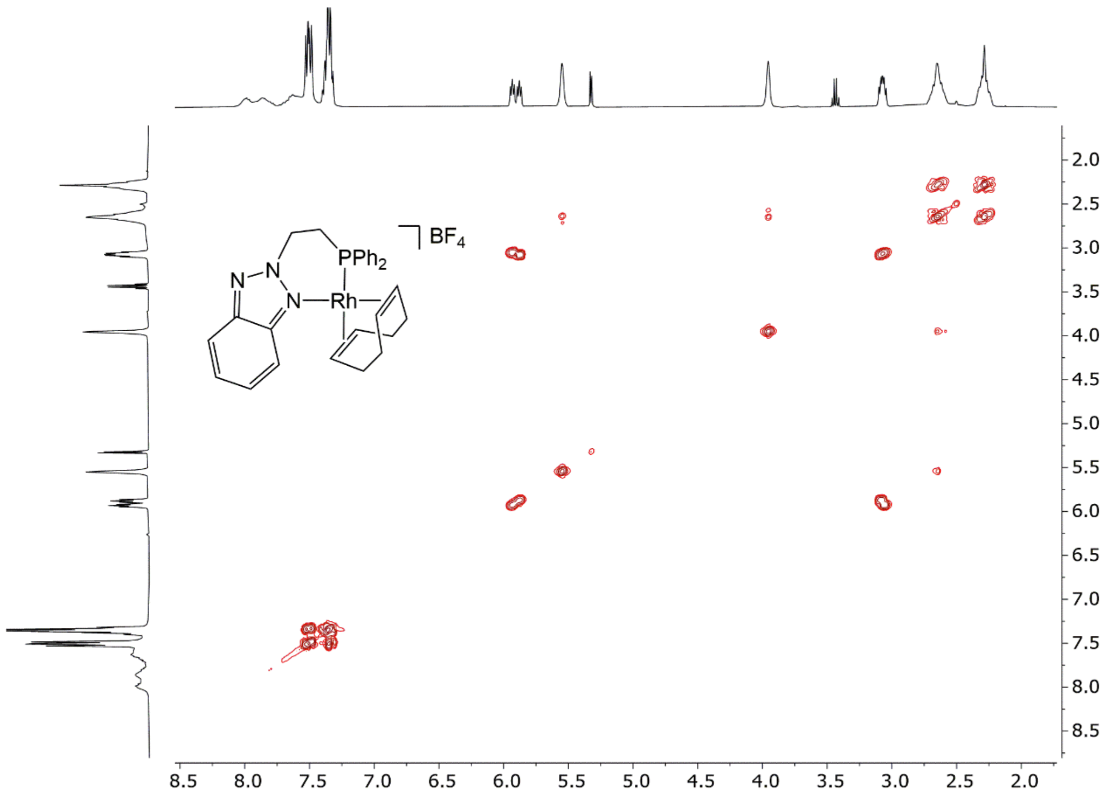

Figure S45. ¹H-¹H COSY spectrum of **Rh-2** in CD_2Cl_2.

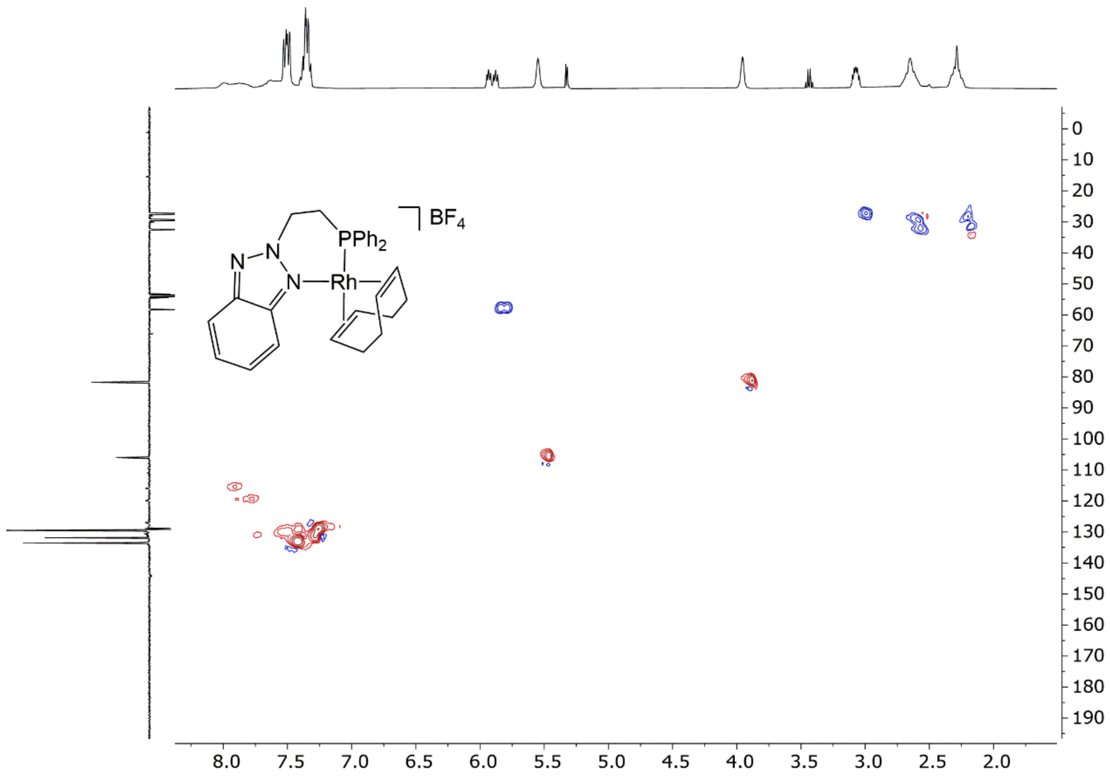

Figure S46. ¹H-¹³C HSQC spectrum of **Rh-2** in CD_2Cl_2.

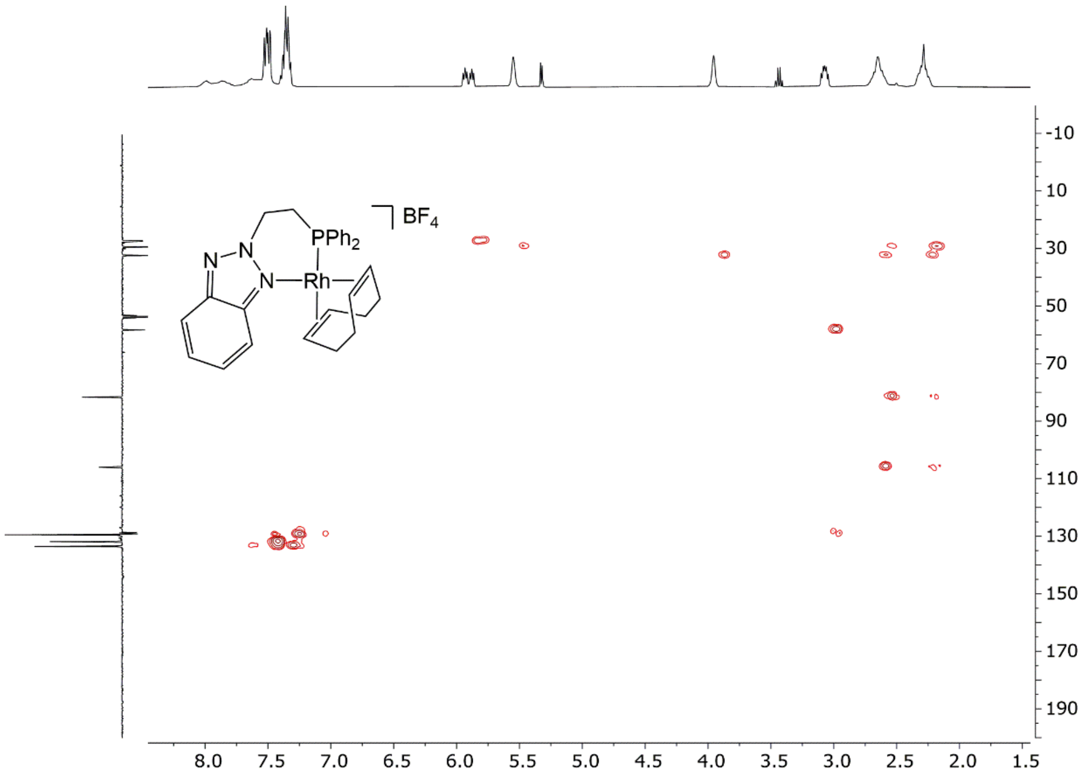

Figure S47. ^{1}H-^{13}C HMBC spectrum of **Rh-2** in CD_2Cl_2.

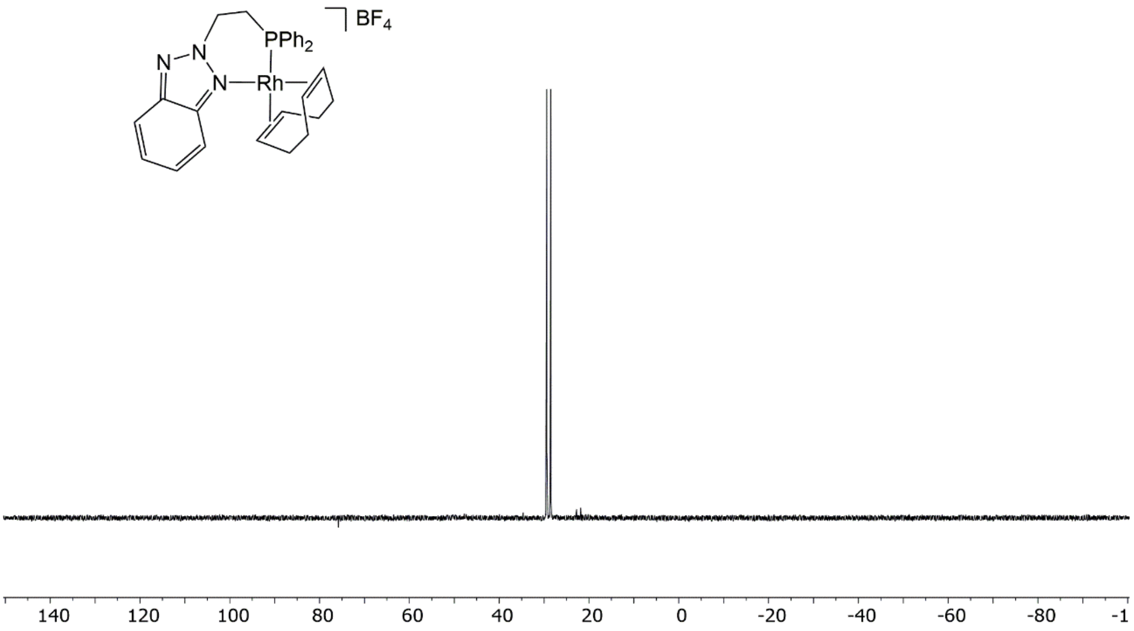

Figure S48. ^{31}P{^{1}H} NMR spectrum of **Rh-2** in CD_2Cl_2.

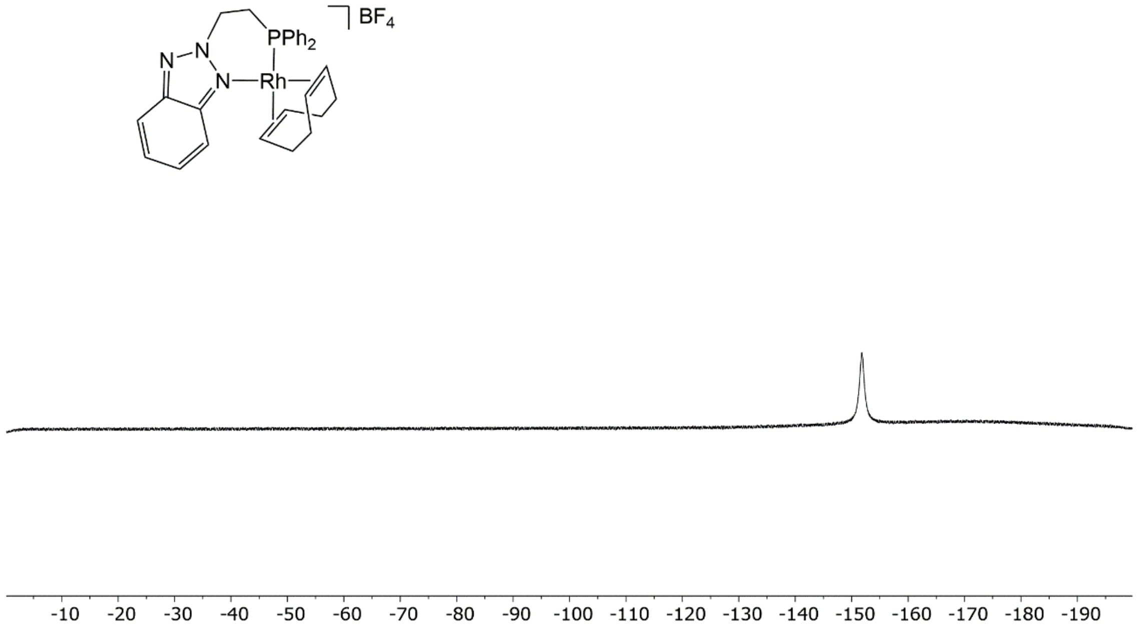

Figure S49. ^{19}F NMR spectrum of **Rh-2** in CD_2Cl_2.

4. Variable temperature ^{1}H NMR spectra of intermediate complexes [IrCl(cod)[PN]] and kinetic analysis using the Arrhenius plot

Ir-1

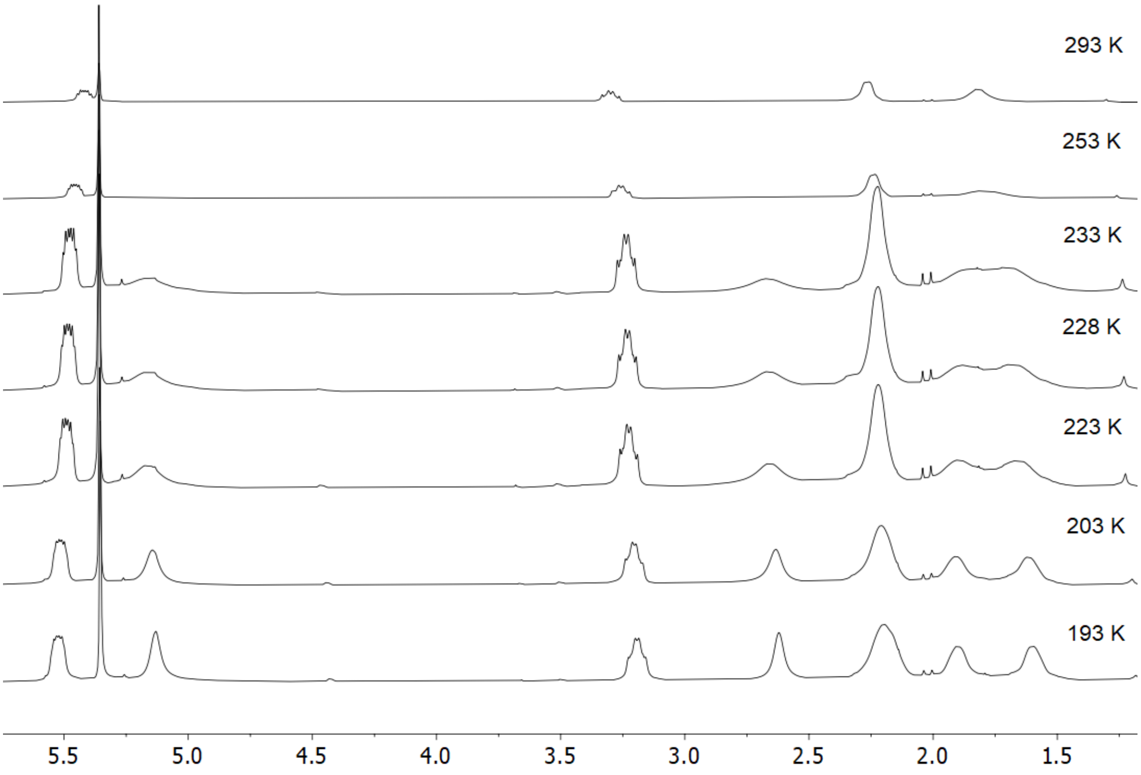

Figure S50. Variable-temperature ^{1}H NMR spectra of [IrCl(cod)**1**] in CD_2Cl_2.

a) Arrhenius Equation

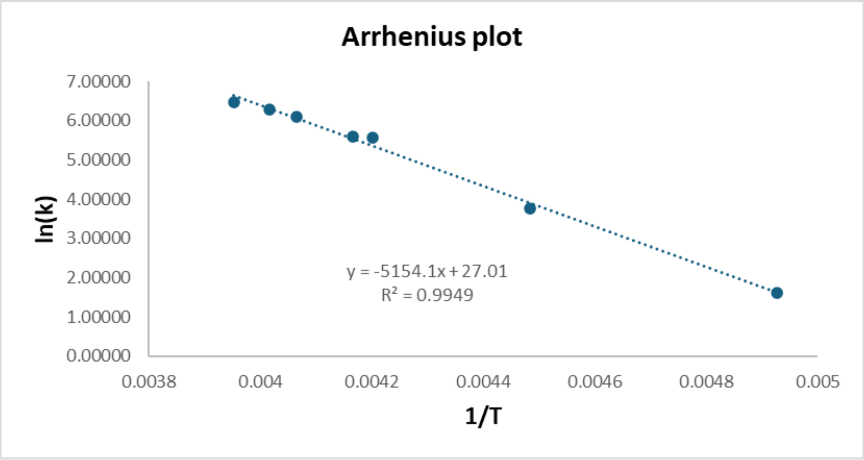

$$\ln(k) = -Ea/R * (1/T) + \ln(A)$$

Comparing with the straight-line equation: $y = mx + b$

we have:

$x = 1/T$

$y = \ln(k)$

$m = -Ea/R$

$b = \ln(A)$

b) Calculation of Ea

$m = -5154.1$

$R = 8.314$ J/mol K

Then:

$Ea = -m * R = 5154.1 * 8.314 = 42851.2$ J/mol = 42.85 kJ/mol (10.24 kcal/mol)

Ir-2

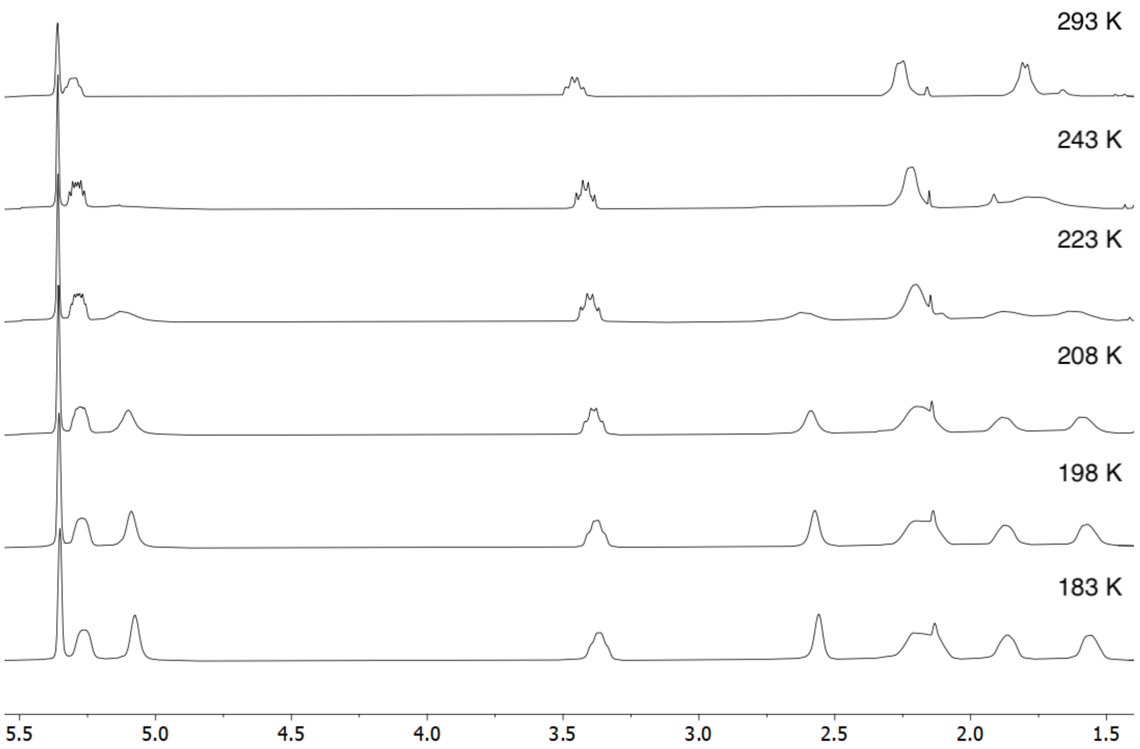

Figure S51. Variable-temperature ^{1}H NMR spectra of [IrCl(cod)**2**] in CD_2Cl_2.

a) Arrhenius Equation

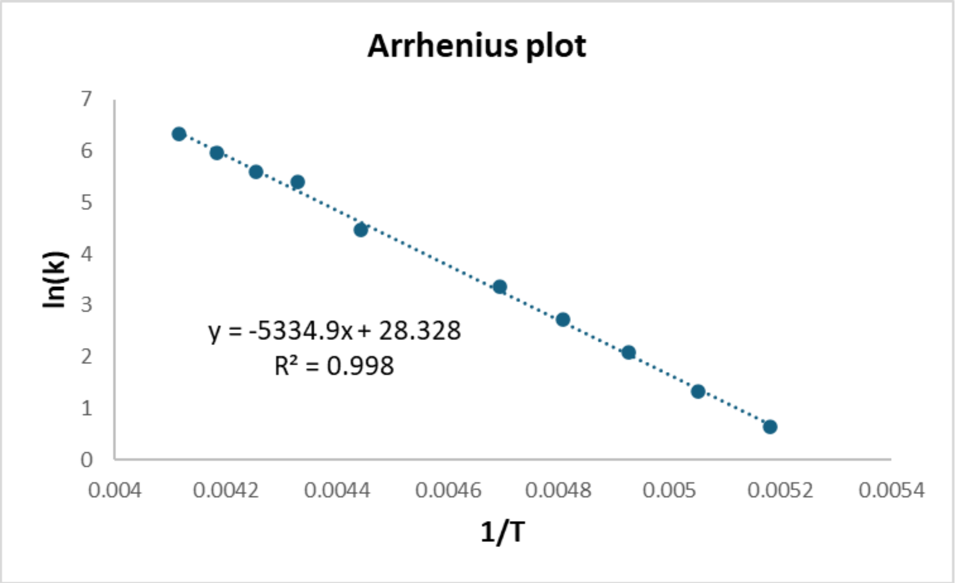

ln(k) = -Ea/R * (1/T) + ln(A)

Comparing with the straight-line equation: $y = mx + b$

we have:

$x = 1/T$

$y = \ln(k)$

$m = -Ea/R$

$b = \ln(A)$

b) Calculation of Ea

$m = -5334.9$

$R = 8.314$ J/mol K

Then:

$Ea = -m * R = 5334.9 * 8.314 = 44371.8$ J/mol = 44.37 kJ/mol (10.60 kcal/mol)

Ir-3

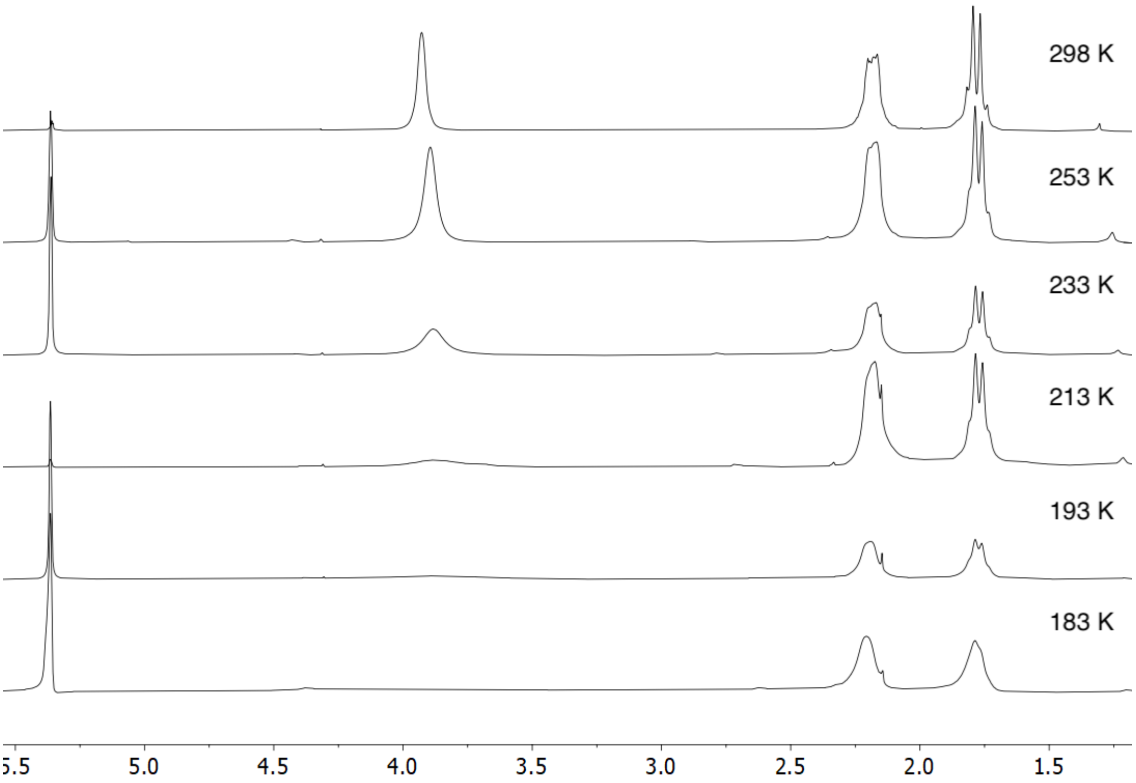

Figure S52. Variable-temperature ^{1}H NMR spectra of [IrCl(cod)**3**] in CD_2Cl_2.

At Tc:

$$\Delta G^{\ddagger}(T_c) \approx RT_c \left[22.96 + \ln\left(\frac{T_c}{\Delta\nu_0}\right)\right]$$

The coalescence temperature (Tc) is approximately −90 ºC (183 K). If we assume a $\Delta\nu_0$ = 120 Hz (similar to those observed in **Ir-1** and **Ir-2**), applying the equation gives:

ΔG=35.61 kJ/mol

Estimated Ea ≈ ΔG+RT=37.13 kJ/mol (8.87 kcal/mol)

5. General procedure for catalytic activity studies

Catalytic reactions were performed in a Man on the Moon series X102 under an inert atmosphere. The conversions of HCOOH to H_2 and CO_2, and the subsequent calculations of TON and TOF, were determined from successive pressure measurements using the ideal gas law. Catalytic reactions were carried out under three different sets of conditions:

Catalysis in HCOOH/H_2O (1:1 v/v). HCOOH (500 µL) was used. First, HCOONa (30 mol%) was added to the flask and dissolved in H_2O (500 µL) and HCOOH (400 µL). The flask was heated to 80 °C. Once the system reached a stable pressure, the catalyst (0.1 mol%) dissolved in HCOOH (100 µL) was added via micropipette, initiating the catalytic reaction.

Catalysis in HCOOH/Et_3N (5:2 molar). HCOOH (500 µL) was used. First, Et_3N (725 µL) and HCOOH (400 µL) were added to the flask. The flask was heated to 80 °C. Once the system reached a stable pressure, the catalyst (0.1 mol%) dissolved in HCOOH (100 µL) was added via micropipette, initiating the catalytic reaction.

7 h experiment TON vs time for Ir-3 under optimal conditions

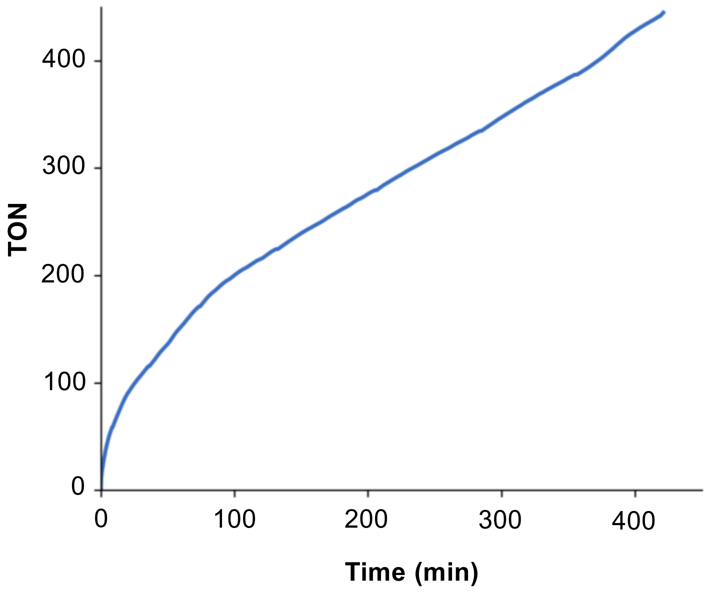

Figure S53. TON vs time of FAD in a 5:2 molar mixture HCOOH/Et_3N using **Ir-3** as catalyst.

6. NMR spectra of stoichiometric experiments

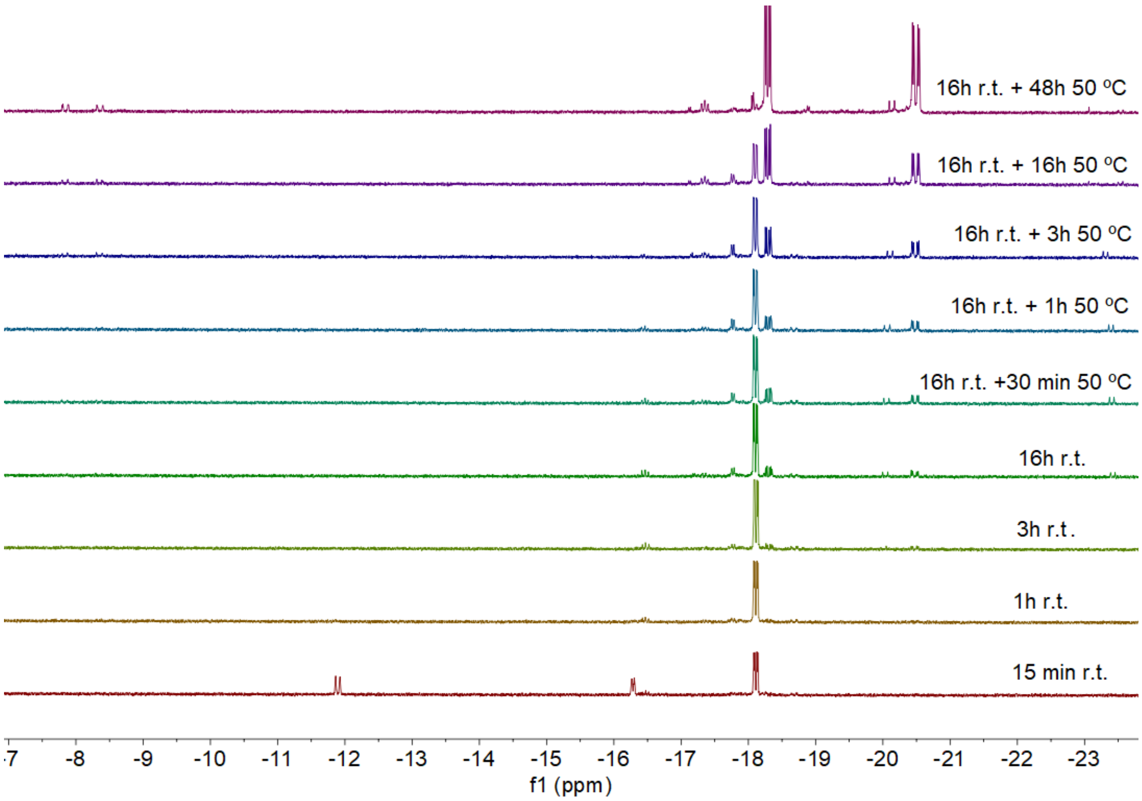

Figure S54. Time evolution (bottom to top) of the reaction monitored by ^{1}H NMR spectroscopy in a Young NMR tube of **Ir-3**, using an excess of HCOOH (10 equiv.) and pyridine (4 equiv.) in CD_2Cl_2, heating at 50 ºC.

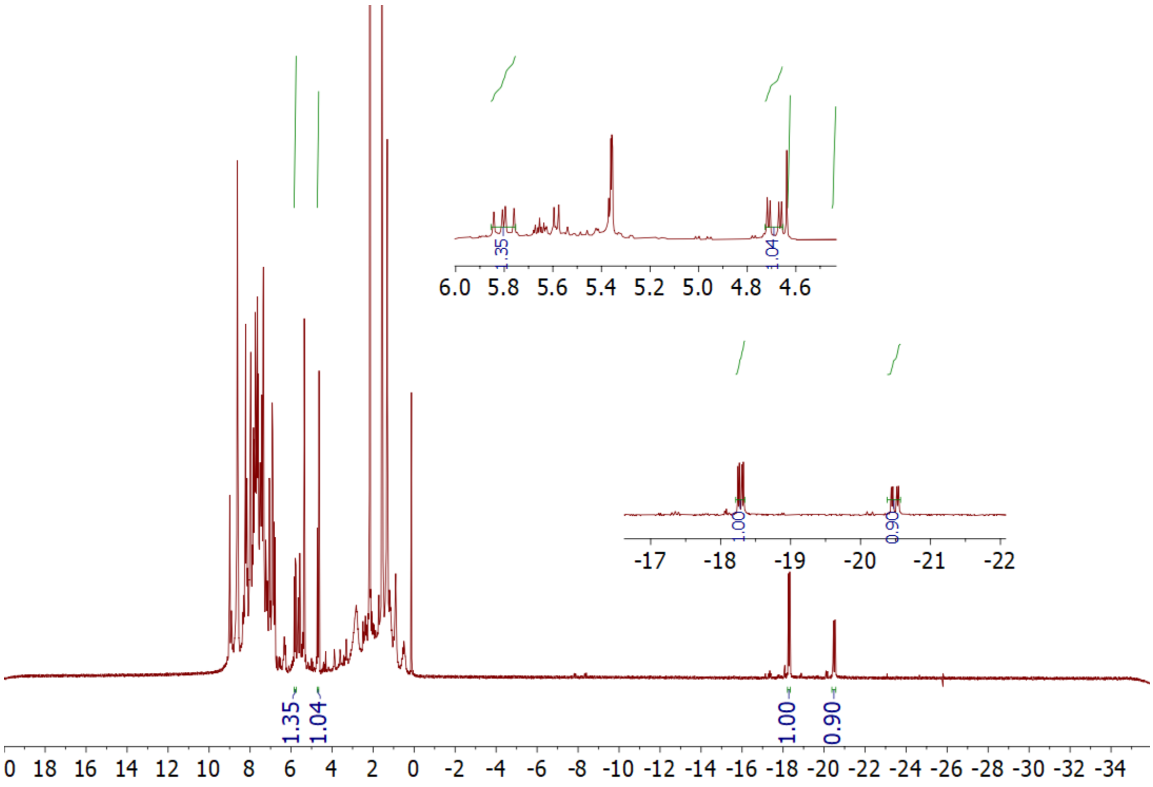

Figure S55. ^{1}H NMR spectrum of stoichiometric experiments in a Young NMR tube of **Ir-3**, using an excess of HCOOH (10 equiv.) and pyridine (4 equiv.) in CD_2Cl_2, after heating at 50 ºC for 16 h.

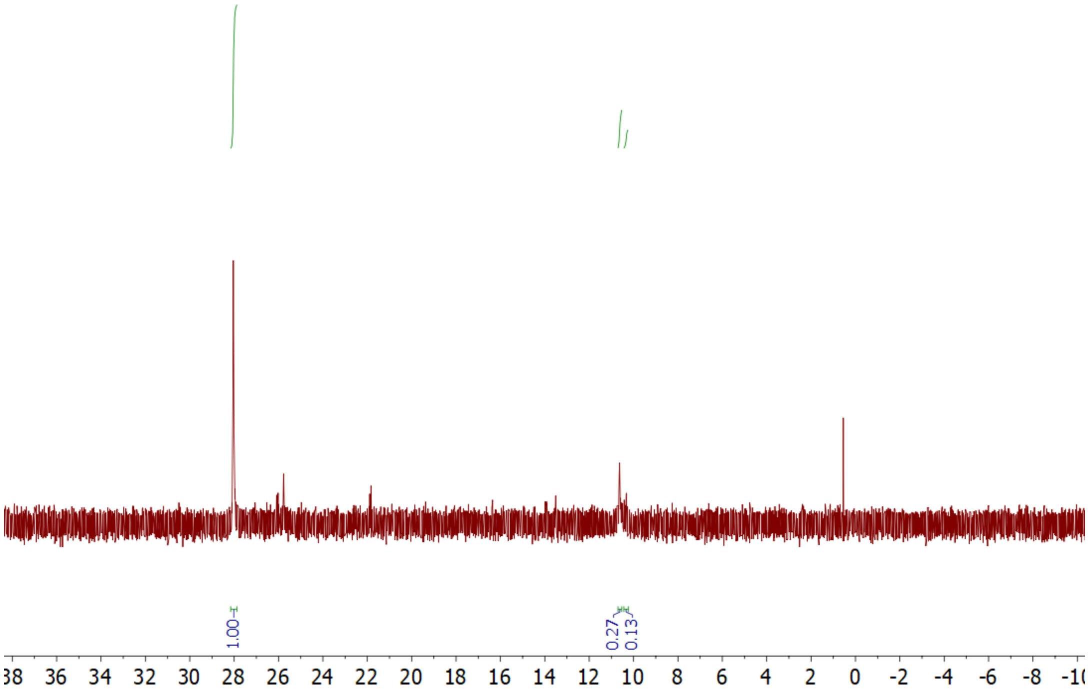

Figure S56. $^{31}P\{^1H\}$ NMR spectrum of stoichiometric experiments in a Young NMR tube of **Ir-3**, using an excess of HCOOH (10 equiv.) and pyridine (4 equiv.) in CD_2Cl_2, after heating at 50 ºC for 16 h.

7. In operando studies

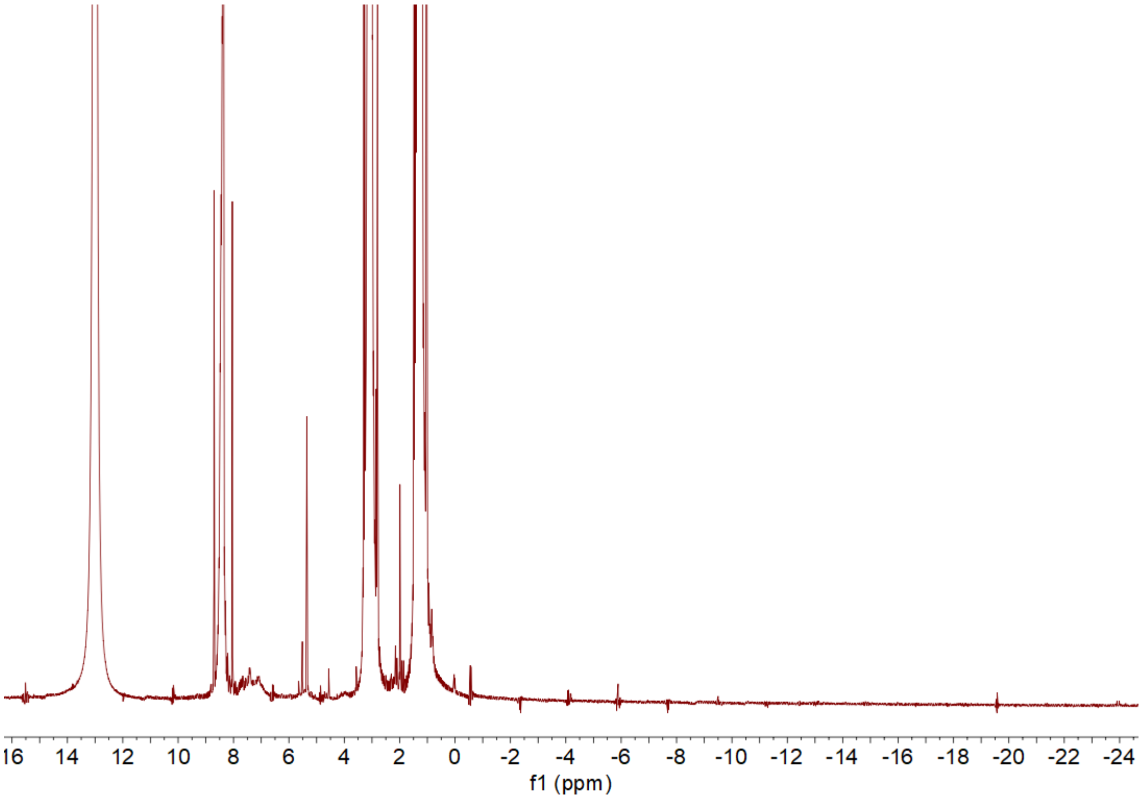

Figure S57. ^{1}H NMR spectrum in operando after 2 h (5:2 HCOOH/Et_3N mixture, 0.1 mol% of **Ir-3**).

8. X-ray diffraction analysis

Crystal data and structure refinement for 2. $C_{20}H_{18}N_3P$, 331.34 g·mol^{-1}, monoclinic, $P2_1/n$, a = 15.2854(6) Å, b = 5.6073(2) Å, c = 19.9300(8) Å, b = 96.314(2)°, V = 1697.83(11) Å^3, Z = 4, D_{calc} = 1.296 g·cm^3, μ = 0.167 mm^{-1}, F(000) = 696, 0.270 x 0.180 x 0.050 mm^3, colourless prism, $\theta_{min}/\theta_{max}$ 2.056/28.288°, index ranges $-20 \le h \le 19$, $-7 \le k \le 7$, $-26 \le l \le 26$, reflections collected/independent 34961/4231 [R(int) = 0.0372], T_{max}/T_{min} 0.7457/0.6677, data/restraints/parameters 4231/0/217, GooF(F^2) 1.055, R_1 = 0.0452 [I>2σ(I)], wR_2 = 0.1193 (all data), largest diff. peak/hole 0.626/–0.259 e·Å^{-3}. CCDC deposit number: 2474509

Crystal data and structure refinement for Ir-1. $C_{28}H_{30}BF_4IrN_3P$, 718.53 g·mol^{-1}, monoclinic, $P2_1/c$, a = 12.7367(15) Å, b = 12.0657(14) Å, c = 17.996(2) Å, β = 107.9750(10)°, V = 2630.6(5) Å^3, Z = 4, D_{calc} = 1.814 g·cm^3, μ = 5.188 mm^{-1}, F(000) = 1408, 0.280 x 0.260 x 0.220 mm^3, colourless prism, $\theta_{min}/\theta_{max}$ 2.380/26.370°, index ranges $-15 \le h \le 15$, $-15 \le k \le 15$, $-22 \le l \le 22$, reflections collected/independent 22054/5372 [R(int) = 0.0517], T_{max}/T_{min} 0.3561/0.1855, data/restraints/parameters 5372/0/343, GooF(F^2) 1.027, R_1 = 0.0278 [I>2σ(I)], wR_2 = 0.0668 (all data), largest diff. peak/hole 1.364/–0.870 e·Å^{-3}. CCDC deposit number: 2474508

Crystal data and structure refinement for Rh-1. $C_{28}H_{30}BF_4N_3PRh$, 629.24 g·mol^{-1}, monoclinic, $P2_1/c$, a = 12.7195(7) Å, b = 12.0775(7) Å, c = 18.0241(10) Å, β = 108.0830(10)°, V = 2632.1(3) Å^3, Z = 4, D_{calc} = 1.588 g·cm^3, μ = 0.762 mm^{-1}, F(000) = 1280, 0.280 x 0.250 x 0.230 mm^3, orange prism, $\theta_{min}/\theta_{max}$ 2.063/28.763°, index ranges $-17 \le h \le 17$, $-16 \le k \le 16$, $-24 \le l \le 24$, reflections collected/independent 45376/6493 [R(int) = 0.0287], T_{max}/T_{min} 0.8015/0.7331, data/restraints/parameters 6493/0/343, GooF(F^2) 1.059, R_1 = 0.0224 [I>2σ(I)], wR_2 = 0.0592 (all data), largest diff. peak/hole 0.512/–0.515 e·Å^{-3}. CCDC deposit number: 2474510

Crystal data and structure refinement for Rh-2·2$C_4H_{10}O$. $C_{36}H_{46}BF_4N_3O_2PRh$, 773.45 g·mol^{-1}, triclinic, $P2_1/c$, a = 9.8451(10) Å, b = 10.1031(10) Å, c = 18.3541(18) Å, α = 99.4090(10)°, β = 99.1600(10)°, γ = 99.3990(10)°, V = 1744.2(3) Å^3, Z = 2, D_{calc} = 1.473 g·cm^3, μ = 0.594 mm^{-1}, F(000) = 800, 0.230 x 0.190 x 0.040 mm^3, yellow prism, $\theta_{min}/\theta_{max}$ 1.146/28.268°, index ranges $-13 \le h \le 13$, $-11 \le k \le 11$, $-24 \le l \le 24$, reflections collected/independent 17960/8566 [R(int) = 0.0268], T_{max}/T_{min} 0.9281/0.7955,

data/restraints/parameters 8566/0/433, GooF(F^2) 1.029, R_1 = 0.0342 [I>2σ(I)], wR_2 = 0.0840 (all data), largest diff. peak/hole 1.067/–0.733 e·Å^{-3}. CCDC deposit number: 2474511.

9. Computational calculations

Table S1. Electron population of the main ELF basins for systems **1**, **Rh-1** and **Ir-1**.

Basin	**1**	**Rh-1**	**Ir-1**
V(N1)	3.04	3.19	3.20
V(N1,N2)	1.57	1.60	1.61
V(N2)	1.05	0.88	0.90
V(N2)	0.80	0.96	0.94
V(N2,C3)	2.14	2.09	2.10
V(C3,C8)	2.68	2.66	2.66
V(C3,C4)	2.74	2.75	2.75
V(C4,C5)	2.96	2.97	2.99
V(C5,C6)	2.63	2.61	2.57
V(C6,C7)	2.93	2.97	2.97
V(C7,C8)	2.74	2.71	2.71
V(C8,N9)	2.21	2.25	2.29
V(N9)	2.99	2.96	3.02
V(N1,N9)	2.15	2.06	2.04
V(N2,C10)	1.81	1.80	1.87
V(C10,C11)	1.86	1.87	1.87
V(P,C11)	1.91	1.98	1.99
V(P,C13)	2.19	2.25	2.27
V(P,C19)	2.19	2.25	2.25
V(P)	2.13	2.12	2.18
V(C25,C26)		2.62	2.50
V(C25)		0.56	0.71
V(C26)		0.58	0.69
V(C26,C27)		1.96	1.94
V(C27,C28)		1.82	1.80
V(C28,C29)		1.97	1.99
V(C29,C30)		3.55	2.72
V(C29)			0.49
V(C30)			0.46
V(C30,C31)		1.97	1.96
V(C31,C32)		1.80	1.81

V(C25,C32)		1.96	1.96

Table S2. Electron population of the main ELF basins for systems **2**, **Rh-2** and **Ir-2**.

Basin	**2**	**Rh-2**	**Ir-2**
V(N1)	3.16	3.25	3.23
V(N1,N2)	1.76	1.68	1.67
V(N2)	0.81	0.61	0.62
V(N2)	0.54	0.91	0.89
V(N2,N3)	1.80	1.80	1.81
V(N3)	3.16	3.08	3.06
V(N3,C4)	2.34	2.34	2.32
V(C4,C6)	2.63	2.64	2.64
V(C4,C5)	2.62	2.65	2.64
V(C5,C6)	3.03	3.02	3.01
V(C6,C7)	2.55	2.53	2.53
V(C7,C8)	3.04	3.04	3.02
V(C8,C9)	2.64	2.66	2.67
V(N1,C9)	2.35	2.33	2.33
V(N2,C10)	2.03	1.99	1.98
V(C10,C11)	1.87	1.87	1.88
V(P,C11)	1.89	1.98	1.99
V(P,C19)	2.20	2.27	2.27
V(P,C13)	2.18	2.24	2.25
V(P)	2.14	2.08	2.16
V(C25,C26)		2.61	2.48
V(C25)		0.59	0.70
V(C26)		0.55	0.69
V(C26,C27)		1.97	1.95
V(C27,C28)		1.81	1.82
V(C28,C29)		1.96	1.95
V(C29,C30)		3.20	2.67
V(C29)		0.41	0.54
V(C30)			0.49
V(C30,C31)		1.97	1.98
V(C31,C32)		1.81	1.80

V(C25,C32)		1.95	1.94

Table S3. Exchange correlation energy contribution (V_{xc}) between pairs of atoms (labelled as A and B) for the most relevant interactions in systems **1**, **Rh-1** and **Ir-1**.

Atom A	Atom B	**1**	**Rh-1**	**Ir-1**
N1	Rh/Ir		–86.1	–97.8
N1	N2	–258.2	–257.8	–256.7
N2	C3	–205.4	–202.9	–203.2
C3	C8	–228.8	–227.1	–226.7
C3	C4	–244.0	–243.3	–243.0
C4	C5	–268.5	–270.0	–270.2
C5	C6	–246.9	–243.8	–243.4
C6	C7	–269.6	–272.9	–273.3
C7	C8	–243.5	–239.9	–239.3
C8	N9	–220.6	–219.2	–219.9
N1	N9	–325.0	–313.3	–311.0
N2	C10	–170.1	–167.1	–166.7
C11	C19	–186.6	–186.6	–186.7
P	C11	–121.7	–120.7	–120.1
P	C13	–127.2	–127.6	–126.8
P	C19	–126.5	–127.3	–127.3
P	Rh/Ir		–118.7	–130.5
C25	Rh/Ir		–82.4	–94.6
C26	Rh/Ir		–80.0	–91.8
C27	Rh/Ir		–2.5	–2.8
C28	Rh/Ir		–3.6	–4.0
C29	Rh/Ir		–64.4	–74.9
C30	Rh/Ir		–61.5	–72.4
C31	Rh/Ir		–2.1	–2.5
C32	Rh/Ir		–4.1	–4.4
C25	C26		–254.9	–246.4
C26	C27		–189.9	–189.2
C27	C28		–181.6	–181.7
C28	C29		–193.4	–193.1
C29	C30		–269.2	–260.5

C30	C31		–191.8	–191.1
C31	C32		–181.1	–181.0
C32	C25		–193.1	–192.5

Table S4. Exchange correlation energy contribution (V_{xc}) between pairs of atoms (labelled as A and B) for the most relevant interactions in systems **2**, **Rh-2** and **Ir-2**.

Atom A	Atom B	**2**	**Rh-2**	**Ir-2**
N1	Rh/Ir		–83.2	–93.4
N1	N2	–283.6	–268.4	–266.6
N2	N3	–284.5	–293.3	–294.0
N3	C4	–225.8	–223.8	–223.6
C4	C9	–220.6	–221.6	–221.8
C4	C5	–235.9	–235.2	–235.1
C5	C6	–276.7	–277.6	–277.6
C6	C7	–239.4	–238.9	–238.9
C7	C8	–276.8	–275.7	–275.5
C8	C9	–235.9	–236.9	–237.2
N1	C9	–226.0	–215.2	–213.6
N2	C10	–165.3	–163.2	–162.8
C10	C11	–186.6	–185.6	–185.6
P	C11	–122.2	–121.4	–121.0
P	C13	–126.6	–127.2	–127.1
P	C19	–127.1	–127.8	–127.7
P	Rh/Ir		–80.6	–128.3
C25	Rh/Ir		–84.1	–93.2
C26	Rh/Ir		–4.1	–96.8
C27	Rh/Ir		–2.1	–4.5
C28	Rh/Ir		–61.6	–2.5
C29	Rh/Ir		–65.5	–73.8
C30	Rh/Ir		–3.8	–78.0
C31	Rh/Ir		–2.5	–4.2
C32	Rh/Ir		–80.6	–2.9
C25	C26		–254.2	–245.5
C26	C27		–193.2	–192.5
C27	C28		–181.2	–181.0
C28	C29		–191.5	–190.7
C29	C30		–268.5	–258.5
C30	C31		–193.5	–193.0

C31	C32		–181.8	–181.7
C32	C25		–190.1	–189.4

Table S5. Coordinates of systems **1**, **2**, **Rh-1**, **Rh-2**, **Ir-1** and **Ir-2** obtained at B3LYP-D3BJ/def2-SVP level.

1

C	3.201725	-0.384103	-0.296538
C	-0.826275	-1.932933	0.353576
H	-0.684158	-1.390341	1.301362
H	-1.626722	-2.668111	0.527528
C	-2.990757	-0.179941	-0.198134
N	2.033436	-1.680123	-1.576955
N	2.993451	-0.824226	-1.585113
P	-1.427829	-0.768127	-0.996277
C	2.282028	-1.045373	0.549542
C	4.111668	0.549047	0.226836
C	0.439111	-2.700654	-0.037192
C	-3.962918	-1.120936	0.188162
C	-3.302864	1.184692	-0.078455
N	1.577501	-1.843704	-0.305629
C	-0.298263	0.670408	-0.739345
C	2.239782	-0.813996	1.933474
C	4.069329	0.786281	1.593780
H	4.814918	1.062981	-0.429996
H	0.715777	-3.408320	0.762208
H	0.278386	-3.275105	-0.959917
C	-5.191855	-0.712726	0.709040
H	-3.766921	-2.190685	0.074797
C	-4.537088	1.593249	0.434546
H	-2.574528	1.937429	-0.384196
C	-0.078100	1.267919	0.514122
C	0.397146	1.161361	-1.853103
H	1.530167	-1.325563	2.585308
C	3.147791	0.111259	2.433228
H	4.758339	1.507094	2.039419
H	-5.927758	-1.462429	1.010555
C	-5.483832	0.648503	0.836797
H	-4.756932	2.660318	0.522140
H	-0.613315	0.903479	1.394012
C	0.817511	2.327193	0.648975
H	0.250128	0.690413	-2.827385
C	1.296207	2.223745	-1.719584
H	3.152118	0.328176	3.504044
H	-6.446315	0.969700	1.241899
H	0.989308	2.772615	1.631388
C	1.508197	2.806262	-0.469735
H	1.843384	2.582827	-2.593959
H	2.219585	3.628263	-0.361112

2

P	-1.504483	0.742175	-0.904584
N	1.948461	1.745412	-0.910221
C	-0.343590	-0.623434	-0.458183
C	-0.209308	-1.136679	0.843326
C	-1.160386	1.983918	0.467308
H	-1.100720	1.490091	1.448778
N	1.304056	2.003125	0.216139
C	2.917304	0.888538	-0.521158
C	-3.366114	-1.278336	-0.090954
H	-2.554180	-1.996637	-0.213643
C	-3.126803	0.090210	-0.296729
C	0.463861	-1.149244	-1.476749
H	-0.828633	-0.743567	1.652219
C	0.710391	-2.148726	1.115443
H	-2.021486	2.668796	0.498629
C	0.096874	2.818872	0.227302
C	2.779516	0.676654	0.887478
C	3.928398	0.232704	-1.261861
C	-4.635769	-1.734001	0.275980
C	-4.203357	0.984226	-0.148294
H	0.379449	-0.742706	-2.487308
C	1.388530	-2.162127	-1.205169
C	1.514197	-2.661271	0.091701
H	0.811199	-2.531215	2.133956
H	0.067752	3.324772	-0.746008
H	0.214819	3.569590	1.022845
C	3.650034	-0.195810	1.581400
N	1.731748	1.412393	1.318068
H	4.027027	0.393548	-2.336453
C	4.764827	-0.616908	-0.566312
C	-5.689642	-0.833298	0.443877
H	-4.798797	-2.803092	0.434589
C	-5.468211	0.530361	0.227984
H	-4.058608	2.051865	-0.336484
H	2.021378	-2.550309	-2.006012
H	2.246607	-3.441607	0.309997
C	4.626810	-0.828812	0.839471
H	3.538028	-0.360007	2.654056
H	5.557101	-1.148064	-1.099204
H	-6.680000	-1.190938	0.735387
H	-6.286917	1.244700	0.346161
H	5.317996	-1.515396	1.334168

Rh-1

C	3.750077	0.510068	0.008268
C	0.256325	-1.488882	-1.979605
H	0.816297	-2.410629	-1.765698
H	-0.446992	-1.709333	-2.794963
C	-2.419332	-1.586600	-0.777573
C	-2.447020	1.857842	-0.072050
H	-2.930688	1.179086	-0.780412
N	1.656235	0.832431	-0.349393
N	2.612596	1.108544	0.474350
P	-0.717409	-1.016044	-0.453828
Rh	-0.360852	1.220013	0.040542
C	3.437330	-0.174136	-1.193130
C	5.060706	0.499357	0.526414
C	1.201740	-0.371941	-2.428648
C	-3.049564	-1.220020	-1.979890

C	-3.153182	-2.272228	0.203044
C	-2.571759	3.338898	-0.403297
C	-2.182430	1.335396	1.198977
N	2.105145	0.066773	-1.375511
C	-0.054814	-2.103851	0.852287
C	4.404331	-0.886547	-1.921527
C	6.013053	-0.205571	-0.189004
H	5.298374	1.028228	1.449990
H	1.826584	-0.702982	-3.269367
H	0.631221	0.508437	-2.760340
C	-4.384305	-1.556954	-2.206721
H	-2.502620	-0.661650	-2.744523
C	-4.491740	-2.602000	-0.026859
H	-2.679621	-2.557105	1.144112
H	-3.321536	3.458642	-1.198856
C	-1.247183	3.974325	-0.870813
H	-2.972127	3.876613	0.467452
H	-2.525220	0.312319	1.389970
C	-1.877202	2.121275	2.453478
C	-0.052026	-3.501847	0.700098
C	0.477749	-1.532046	2.017907
H	4.168228	-1.413996	-2.846516
C	5.687343	-0.886976	-1.393316
H	7.042168	-0.242888	0.172886
H	-4.862928	-1.275581	-3.147162
C	-5.107563	-2.249380	-1.229904
H	-5.053720	-3.140925	0.738915
H	-1.118136	3.790901	-1.948841
H	-1.288767	5.073377	-0.756367
C	-0.026730	3.428024	-0.167852
C	-0.989993	3.361424	2.220826
H	-2.821191	2.413815	2.950033
H	-1.370840	1.438731	3.154665
H	-0.477532	-3.960517	-0.196164
C	0.480128	-4.312681	1.702138
H	0.476989	-0.445606	2.133853
C	1.011784	-2.348898	3.018578
H	6.476245	-1.428705	-1.919252
H	-6.152538	-2.511963	-1.407575
H	0.904271	3.480202	-0.742514
C	0.085734	3.125859	1.178393
H	-1.603510	4.225987	1.933950
H	-0.506487	3.634156	3.170260
H	0.477638	-5.398130	1.581347
C	1.013584	-3.736529	2.861370
H	1.426968	-1.898199	3.922467
H	1.090845	2.905746	1.551967
H	1.430013	-4.374476	3.644039

Rh-2

Rh	0.197065	-1.198297	0.030975
P	0.889887	0.961047	0.512647
N	-1.635709	-0.515657	0.837583
C	1.707588	-1.543854	-1.465724
H	2.075465	-0.562915	-1.788651
C	1.083971	-2.363523	-2.571599
C	-0.047389	2.205210	-0.436820
C	-0.006215	3.568396	-0.096952
C	0.449052	1.295788	2.298675
H	0.020584	2.304958	2.377935
N	-1.704830	0.105141	2.024549
C	-2.861864	-0.329344	0.286622
C	3.084682	2.157635	-0.766404
H	2.364365	2.527233	-1.498488
C	2.644599	1.428162	0.349755
C	2.198159	-2.026393	-0.246587
H	1.875073	-2.762797	-3.233309
C	0.177374	-3.507756	-2.072621
H	0.491179	-1.677228	-3.197765
C	-0.827478	1.787159	-1.525582
H	0.613976	3.913209	0.734249
C	-0.748325	4.494188	-0.830103
H	1.370859	1.278703	2.896381
C	-0.523557	0.257014	2.866770
C	-3.626278	0.420681	1.232542
C	-3.406438	-0.695476	-0.964864
C	4.445676	2.423750	-0.936783
C	3.586657	0.957453	1.281286
C	2.274046	-3.491179	0.158649
H	2.879877	-1.362468	0.291575
H	-0.516281	-3.787437	-2.879020
H	0.771194	-4.407488	-1.862851
C	-0.629739	-3.127111	-0.845404
H	-0.851501	0.728623	-1.793470
C	-1.570791	2.716821	-2.256842
C	-1.534172	4.068594	-1.907586
H	-0.713823	5.552243	-0.561819
H	-0.044546	-0.729439	2.940821
H	-0.893721	0.556462	3.854739
C	-4.964481	0.795324	0.963533
N	-2.847370	0.673486	2.308119
H	-2.820295	-1.236924	-1.706162
C	-4.710674	-0.312856	-1.209346
C	5.375602	1.964814	-0.000644
H	4.778987	2.997611	-1.804117
C	4.944103	1.231188	1.109084
H	3.267603	0.371636	2.147331
H	2.422628	-4.116467	-0.732395
C	1.037616	-3.956956	0.950524
H	3.169394	-3.639089	0.779765
C	-0.256429	-3.345443	0.470005
H	-1.684611	-2.897954	-1.017259
H	-2.181145	2.382822	-3.098395
H	-2.116646	4.796086	-2.477116
C	-5.484003	0.418273	-0.256629
H	-5.541514	1.361720	1.695197
H	-5.171548	-0.572910	-2.164689
H	6.438114	2.178488	-0.134483
H	5.667669	0.869710	1.842853
H	0.959542	-5.059837	0.930652
H	1.161151	-3.685364	2.010640
H	-1.049745	-3.284107	1.223219
H	-6.510870	0.686419	-0.512426

Ir-1

C	-3.963276	-0.005517	0.013389
C	-0.268942	1.869255	-1.734629
H	-0.784634	2.736236	-1.296905
H	0.361793	2.237647	-2.556223
C	2.520906	1.241619	-1.073657
C	2.041777	-1.986077	-0.066509
H	2.626273	-1.398935	-0.780029
N	-1.871569	-0.404722	-0.267956
N	-2.877520	-0.672966	0.500843
P	0.824034	1.117450	-0.415728
Ir	0.088608	-1.028776	0.123359
C	-3.568315	0.712512	-1.143519
C	-5.294606	0.041372	0.475400
C	-1.288900	0.871520	-2.286594
C	2.781187	0.813227	-2.386955
C	3.589000	1.627963	-0.249896
C	1.943028	-3.472412	-0.391627

C	1.904765	-1.437537	1.226028
N	-2.240231	0.424260	-1.279366
C	0.733019	2.289627	0.975122
C	-4.472099	1.494665	-1.882137
C	-6.183806	0.813916	-0.250335
H	-5.593959	-0.513161	1.365367
H	-1.879411	1.327564	-3.092677
H	-0.790637	-0.019306	-2.698102
C	4.088091	0.793147	-2.874398
H	1.965170	0.479676	-3.033357
C	4.897304	1.600507	-0.741236
H	3.401945	1.959083	0.773065
H	2.638171	-3.698920	-1.212824
C	0.523671	-3.908360	-0.805482
H	2.293867	-4.060274	0.468244
H	2.435114	-0.497260	1.416557
C	1.555527	-2.199603	2.485559
C	1.103864	3.635738	0.809105
C	0.261332	1.845444	2.219800
H	-4.174326	2.048629	-2.773174
C	-5.776156	1.528393	-1.410239
H	-7.225779	0.881094	0.067765
H	4.280954	0.465450	-3.898181
C	5.148692	1.187165	-2.051411
H	5.722819	1.907715	-0.095635
H	0.387914	-3.730397	-1.883313
H	0.397950	-4.996913	-0.659118
C	-0.572444	-3.162096	-0.081297
C	0.459203	-3.266911	2.283924
H	2.465365	-2.661411	2.911030
H	1.214655	-1.463721	3.231049
H	1.483638	3.989166	-0.152884
C	1.002019	4.523484	1.879862
H	-0.028737	0.798482	2.340267
C	0.161260	2.739614	3.289312
H	-6.517722	2.124034	-1.946669
H	6.171683	1.169924	-2.433146
H	-1.526879	-3.107480	-0.616024
C	-0.596759	-2.846880	1.276783
H	0.904575	-4.222862	1.976289
H	-0.032797	-3.458089	3.248489
H	1.292982	5.568027	1.749312
C	0.531060	4.075824	3.120079
H	-0.206528	2.390303	4.256386
H	-1.557892	-2.517680	1.683751
H	0.453936	4.773767	3.956680

Ir-2

Ir	0.138006	-1.059568	0.012052
P	0.895402	1.093600	0.507696
N	-1.684275	-0.347599	0.832551
C	1.631270	-1.455066	-1.489405
H	2.049956	-0.500135	-1.829258
C	1.017593	-2.283760	-2.596253
C	0.037921	2.372915	-0.467746
C	0.171091	3.736366	-0.152620
C	0.421876	1.456802	2.280754
H	-0.014979	2.464278	2.330084
N	-1.740517	0.272779	2.023403
C	-2.918120	-0.164658	0.294086
C	3.178749	2.160080	-0.728363
H	2.497439	2.556034	-1.483603
C	2.673915	1.469360	0.384645
C	2.109780	-1.947867	-0.256430
H	1.814698	-2.729769	-3.218869
C	0.056071	-3.380362	-2.090674
H	0.468636	-1.594911	-3.257965
C	-0.774719	1.986489	-1.544468
H	0.817109	4.053616	0.669972
C	-0.511736	4.695668	-0.899877
H	1.333336	1.459721	2.894430
C	-0.552971	0.425268	2.858129
C	-3.673140	0.578809	1.252010
C	-3.473650	-0.525152	-0.953654
C	4.555800	2.352237	-0.866512
C	3.565128	0.961530	1.345842
C	2.159640	-3.419179	0.136507
H	2.831475	-1.312129	0.263608
H	-0.650064	-3.632811	-2.894893
H	0.605642	-4.306394	-1.873225
C	-0.733931	-2.952836	-0.864853
H	-0.867997	0.926744	-1.792629
C	-1.458938	2.950477	-2.289040
C	-1.329973	4.303095	-1.965879
H	-0.405513	5.753931	-0.652003
H	-0.077928	-0.562565	2.937451
H	-0.919680	0.734637	3.844333
C	-5.016054	0.948759	1.000708
N	-2.883288	0.831701	2.319398
H	-2.892282	-1.058301	-1.704434
C	-4.782582	-0.146943	-1.180516
C	5.436077	1.857333	0.098806
H	4.940886	2.896062	-1.731736
C	4.938990	1.161260	1.205343
H	3.193141	0.401710	2.207873
H	2.298445	-4.040148	-0.759318
C	0.909986	-3.863465	0.920604
H	3.050101	-3.588888	0.758682
C	-0.358897	-3.185763	0.459926
H	-1.792105	-2.736788	-1.033362
H	-2.094116	2.642722	-3.122225
H	-1.865350	5.057093	-2.547055
C	-5.548333	0.574977	-0.214920
H	-5.586136	1.510021	1.741703
H	-5.252735	-0.402908	-2.132415
H	6.511432	2.013154	-0.010080
H	5.624056	0.771049	1.961014
H	0.787809	-4.960817	0.865607
H	1.044442	-3.627233	1.987593
H	-1.156485	-3.140554	1.210323
H	-6.579242	0.839899	-0.457427
